# Identification of SRS transcription factor family in *Solanum lycopersicum*, and functional characterization of their responses to hormones and abiotic stresses

**DOI:** 10.1186/s12870-023-04506-2

**Published:** 2023-10-14

**Authors:** Wang Lu, Yan Wang, Yuan Shi, Qin Liang, Xiangyin Lu, Deding Su, Xin Xu, Julien Pirrello, Ying Gao, Baowen Huang, Zhengguo Li

**Affiliations:** 1 Key Laboratory of Plant Hormones and Development Regulation of Chongqing, School of Life Sciences, Chongqing University, Chongqing, 401331 China; 2 Center of Plant Functional Genomics, Institute of Advanced Interdisciplinary Studies, Chongqing University, Chongqing, 401331 China; 3 Laboratory of Plant Science Research, Fruit Genomics and Biotechnology, UMR5546, University of Toulouse, CNRS, UPS, Toulouse-NP, Toulouse, France

**Keywords:** SRS family, *Solanum lycopersicum*, Genome-wide analysis, Gene expression analysis, Gene evolutionary analysis

## Abstract

The SHI RELATED SEQUENCE (SRS) family plays a vital role in the development of multiple plant organs such as floral meristem determinacy, organ morphogenesis, and signal transduction. Nevertheless, there is little understanding of the biological significance of tomato SRS family at this point. Our research identified eight SlSRS family members and classified them into three subfamilies based on phylogenetics, conserved motifs, and characteristic domain analysis. The intraspecies and interspecies collinearity analysis revealed clues of SRS family evolution. Many *cis*-elements related to hormones, stresses, and plant development can be found in the promoter region of *SlSRS* genes. All of eight SlSRS proteins were located in the nucleus and possessed transcriptional activity, half of which were transcriptional activators, and the other half were transcriptional repressors. Except for *SlSRS1*, which showed high transcript accumulation in vegetative organs, most *SlSRS* genes expressed ubiquitously in all flower organs. In addition, all *SlSRS* genes could significantly respond to at least four different plant hormones. Further, expression of *SlSRS* genes were regulated by various abiotic stress conditions. In summary, we systematically analyzed and characterized the SlSRS family, reviewed the expression patterns and preliminarily investigated the protein function, and provided essential information for further functional research of the tomato *SRS* genes in the determination of reproductive floral organs and the development of plants, and possibly other plants.

**Supplementary Information:**

The online version contains supplementary material available at 10.1186/s12870-023-04506-2.

## Background

SRS proteins belong to a plant-specific family of transcription factors contain a cysteine-rich RING-like zinc finger domain and distribute in many plant species and have an important role in the development of organs such as flowers, leaves and roots [1–6]. The first SRS transcription factor (TF) Lateral root primordium1 (LRP1) was identified from *Arabidopsis thaliana* [7]. Since Fridborg et al. identified SHORT INTERNODES (SHI) protein from an *Arabidopsis thaliana* mutant *shi* with gibberellic acid (GA) biosynthesis defect [1], this family was officially named SRS family in further research [2]. SRS family genes contain a cysteine-rich RING-like zinc finger domain (CX_2_CX_7_CX_4_CX_2_C_2_X_6_C, where X denotes variable amino acids) which belongs to C3HC3H type ring domain for binding to RNA, protein, and lipid substrates [1, 2]. SRS is a small plant-specific family with a small number of members, usually less than 10, whereas there are 21 members in soybean. In addition, most SRS TFs contain a highly conserved IXGH domain at the C-terminal with transcriptional activity [2]. There is evidence that the IXGH domain could mediate intrafamily homo-/heterodimerization [8]. Whether this is the primary mode of SRS TFs to function needs further investigation. The *Arabidopsis* SRS protein STYLISH1 (AtSTY1) specifically binds to the ACTCTAC element, in vitro [8]. This element may be the specific binding site of SRS TF, but more evidence is required.

At present, SRS TFs have been reported to play a critical role in many physiological and biochemical processes, including hormone biosynthesis, signal transduction, multiple plant organs growth and development, photomorphogenesis, and abiotic stress responses. Some of the 11 *SRS* genes in *Arabidopsis thaliana* have been intensively studied. Members of the *Arabidopsis thaliana* SRS family are functionally conservative and pleiotropic [3, 9, 10]. *AtLRP1* expression is affected by auxin and histone deacetylation and it acts downstream of lateral root (LR) forming auxin response modules to negatively regulate LRP development by modulating auxin homeostasis [7, 11, 12]. AtSHI, as a negative regulator in GA signaling pathway, regulates pistil morphology and promotes flowering [1, 2]. AtSTY1 and AtSTY2 redundantly regulate the pistil development in *Arabidopsis thaliana* [13]. AtSTY1 can regulate auxin biosynthesis by directly binding to the *YUCCA4* (*AtYUC4*) and *AtYUC8* promoters to influence plant development, including cell expansion, stamen and leaf development, and flowering time regulation [8, 14–16]. AtSRS5 has multiple effects, participating in defense response [17], promoting photomorphogenesis [18], and negatively regulating LR formation and being inhibited by auxin [19]. AtSRS7 mutation leads to plant dwarfing and another dehiscence disruption, which may be related to jasmonic acid (JA) signalling pathway obstruction [20].

In other species, there are several enlightening researches on the function of SRS TFs. In Populus, two SHI-like proteins have redundant functions on tree growth, form and wood properties [21]. In barley, a grass-specific SRS protein short awn2 (HvLks2) can regulate awn elongation and pistil morphology [4]. Another barley grass-specific SRS protein Six-rowed spike2 (HvVRS2) is involved in the regulation of inflorescence pattern and plant architecture by maintaining hormonal homeostasis and gradients [5]. ROOTLESS WITH UNDETECTABLE MERISTEM1 (ZmRUM1) protein of maize Aux/IAA-ARF module can directly bind to *ZmLRP1* promoter and inhibit its expression [22]. In rice, another important monocotyledon crop, OsSHI1 regulates plant architecture by modulating the transcriptional activity of *IDEAL PLANT ARCHITECTURE1* (*OsIPA1*) which acts as a key TF regulating tiller outgrowth and panicle branching [6].

Recently, several studies indicate that *SRS* family genes may be involved in abiotic stress tolerance. In the forage species alfalfa (*Medicago sativa*), expression of *MeSRS* was induced by cold and salt, suggesting that *SRS* genes may play important roles in tissue-dependent signaling pathway [23]. In *Melilotus albus*, after undergoing several stresses and hormone treatments, including salinity, low temperature, salicylic acid (SA), and methyl jasmonate (MeJA), expression of *MaSRS* genes was induced [24]. *GmSRS18* of Soybean (*Glycine max*) has been shown to reduce drought and salt tolerance, as well as stress-related genes expression and physiological indicators such as chlorophyll, proline, and relative electrolyte leakage [25]. In *Gossypium hirsutum*, GhSRS21 negatively regulates salt tolerance in a manner dependent on reactive oxygen species metabolic process [26].

*Solanum lycopersicum* L. is an excellent model plant to study fruit development and ripening, quality formation and postharvest preservation technology of fleshy fruits. *Expression of Terpenoids 1* (*SlEOT1*) is the only reported tomato *SRS* gene [27], which is homologous to *Arabidopsis thaliana AtSTY1*. *SlEOT1* is specifically expressed in glandular trichomes, and the protein directly activates *TERPENE SYNTHASE5* (*SlTPS5*) transcription and regulates terpene biosynthesis. So far, the SRS TFs have been reported to be involved in the biosynthesis and signal transduction of various phytohormones, suggesting the potential ability to regulate the development of various organs and many physiological and biochemical processes in plants. However, little is known about the molecular mechanism underlying the role of SlSRS TFs in tomato fruit development and quality. Our aim was to investigate SlSRS family’s evolution and function by a systematic comprehensive characterization basedon genome-wide identification, including their phylogenetic relationship, conserved amino acid residues within the RING-like zinc finger domain and IXGH domain, chromosomal distribution and collinearity analysis, and putative *cis*-elements. Our study also examined their subcellular localization, transcriptional activity, and spatiotemporal expression patterns. Moreover, we detailed the response of *SlSRS* genes to nine key plant hormones and to various abiotic stresses. Using our results, we provide valuable information about the functional and mechanism analysis of tomato *SRS* genes. In addition, this study may provide a foundation for future research into plant hormone signaling and stress tolerance.

## Results

### Identification and phylogenetic analysis of SlSRS family members

We obtained 8 candidate proteins using HMMER search and 32 candidate proteins using BLASTP tools. After combining results from HMMER search and BLASTP, a total of 32 candidate SlSRS family members in *Solanum lycopersicum* genome were verified by NCBI-CDD. Finally, 8 *SlSRS* genes were identified in the *Solanum lycopersicum* genome and named as *SlSRS1* to *SlSRS8* according to their physical position on tomato chromosomes, among which *SlSRS2* was reported and named *SlEOT1* [27]. Their basic information as well as protein physical and chemical parameters have been shown in Table 1. The length of SlSRS proteins ranged from 187 aa (SlSRS7) to 380 aa (SlSRS5). Their molecular weight ranged from 21404.15 Da (SlSRS7) to 41444.32 Da (SlSRS6). The pI (isoelectric point) values of five of the eight SlSRS proteins were greater than 7, which indicates that they are basic proteins, and the remaining three were acidic. The aliphatic index values usually characterize the global protein thermostability [28]. These values of SlSRS proteins ranged from 48.00 (SlSRS1) to 65.76 (SlSRS6), showing strong thermostability. The GRAVY value of SlSRS proteins ranged from − 0.993 (SlSRS4) to -0.694 (SlSRS8), showing strong hydrophilicity.

**Table 1:** Detailed information of 8 *SlSRS* genes and their encoding proteins

Gene name	Gene ID	AA	MW (Da)	pI	A.I	GRAVY	Best Hit in Arabidopsis
*SlSRS1*	*Solyc01g110140*	215	24228.94	8.62	48.00	-0.825	*AtSRS5*
*SlSRS2*	*Solyc02g062400*	350	38363.98	7.36	49.66	-0.820	*AtSTY1*
*SlSRS3*	*Solyc02g084680*	345	37112.79	6.40	49.57	-0.740	*AtSTY1*
*SlSRS4*	*Solyc03g033680*	321	36412.66	6.25	48.04	-0.993	*AtSRS5*
*SlSRS5*	*Solyc04g080970*	380	40790.65	8.65	55.21	-0.703	*AtSTY1*
*SlSRS6*	*Solyc08g077450*	373	41444.32	5.72	65.76	-0.797	*AtSRS11*
*SlSRS7*	*Solyc10g054070*	187	21404.15	9.07	51.55	-0.810	*AtSRS3*
*SlSRS8*	*Solyc11g064800*	350	37470.82	8.40	53.20	-0.694	*AtLRP1*

Note: AA, number of amino acids; MW, molecular weight, Da; pI, isoelectric point; A.I. aliphatic index; GRAVY, grand average of hydropathicity score

**Fig. 1:**
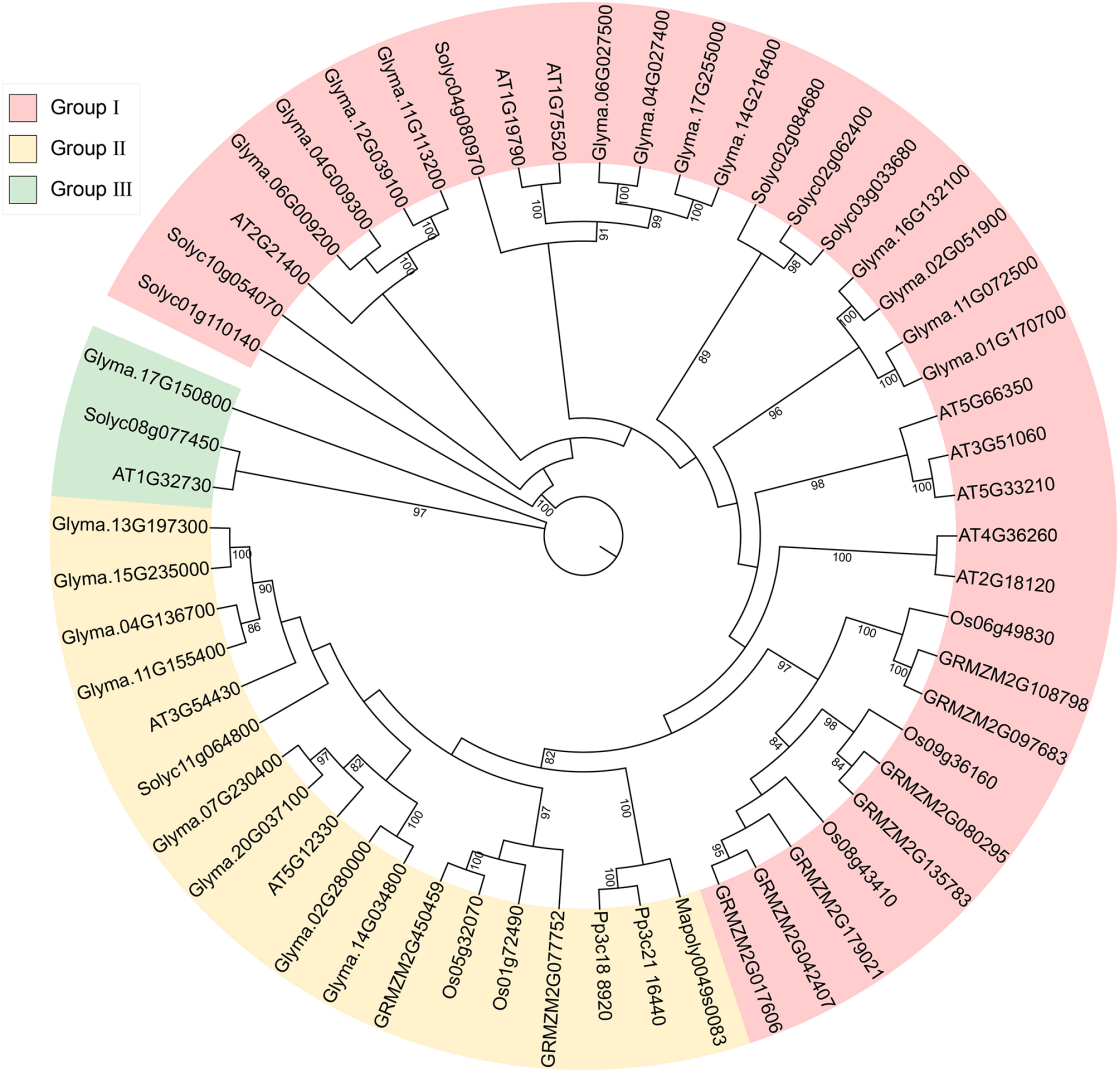
Phylogenetic tree of 57 SRS proteins within *Solanum lycopersicum* (8), *Arabidopsis thaliana* (11), *Glycine max* (21), *Oryza sativa* (5), *Zea mays* (9), *Marchantia polymorpha* (1) and *Physcomitrella patens* (2). Bootstrap values greater than 80 are presented at the corresponding node. Colored ranges represent corresponding subfamily

We selected dicotyledons *Arabidopsis thaliana* [3] and *Glycine max* [25], monocots *Oryza sativa* [29] and *Zea mays*, and primitive plants *Marchantia polymorpha* and *Physcomitrella patens*, together with *Solanum lycopersicum*, for phylogenetic analysis. The selected species contain 11, 21, 5, 9, 1, and 2 SRS family members, respectively. As shown in Fig. 1, a total of 57 genes can be clearly divided into three subfamilies (Group I, II and III), and more than half of them belong to Group I. Interestingly, only dicotyledons contain Group III members. In tomato, there are 6 members in Group I, 1 member in Group II and 1 member in Group III.

### Motif prediction and conserved domain analysis of SlSRS proteins

**Fig. 2:**
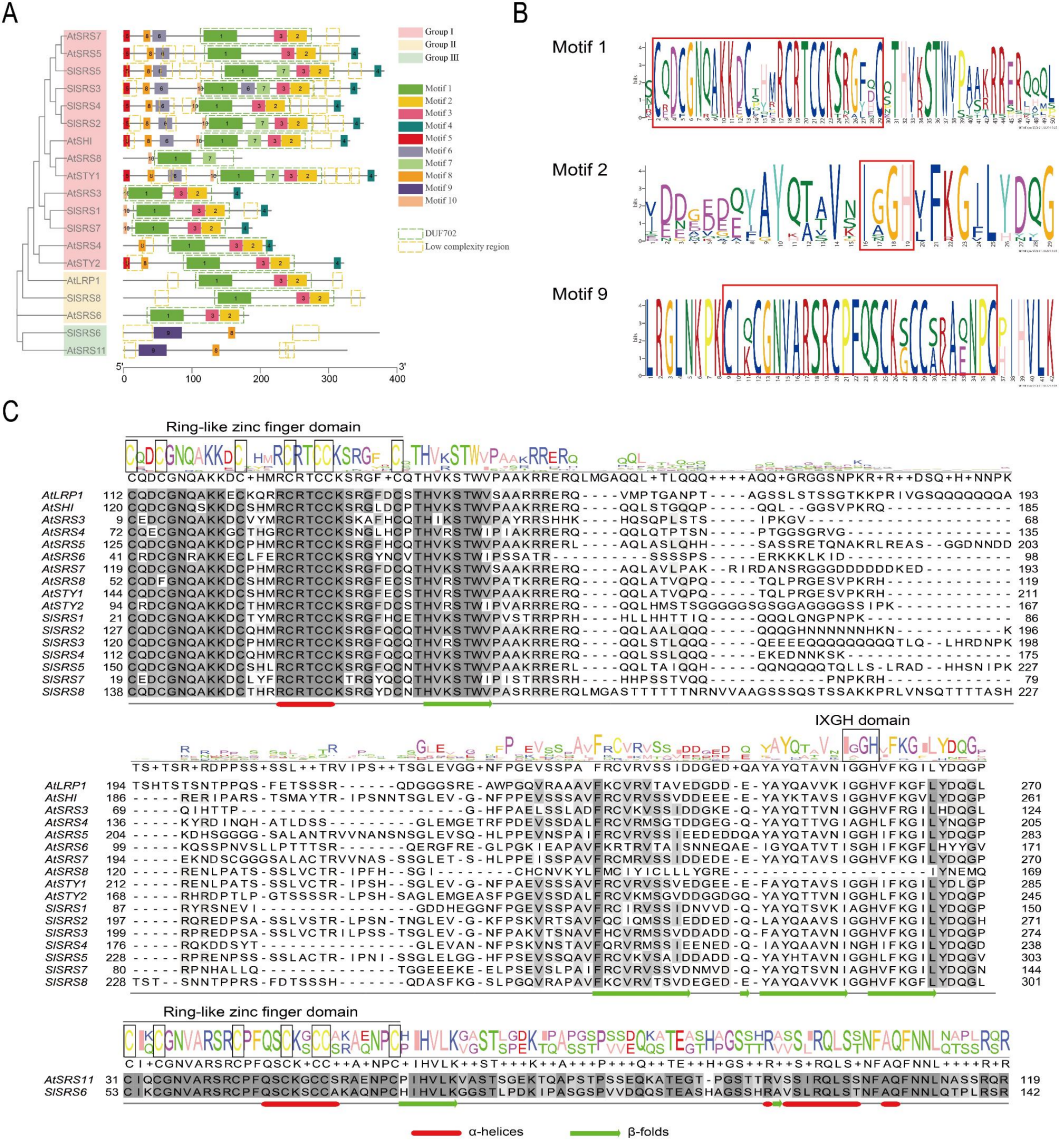
Protein structure of AtSRS and SlSRS proteins. **A**, the phylogenetic tree, conserved motifs and conserved domains. Different colored boxes indicate different subfamilies, motifs, and domains. **B**, sequence logos of amino acid residues of Motif 1, Motif 2, and Motif 9. Red boxes indicate the RING-like zinc finger domain and the IXGH domain. **C**, Alignment, sequence logos of amino acid residues, and protein secondary structure of AtSRS and SlSRS proteins. The RING-like zinc finger domain and the IXGH domain are marked on sequence logos of amino acid residues

Based on *Arabidopsis thaliana* SRS family, we predicted conserved motifs and analyzed characteristic domains of proteins of SlSRS family members using SMART analysis(Fig. 2A). Eleven AtSRS proteins and 8 SlSRS proteins were significantly divided into three subfamilies, and there were obvious differences in protein structure among members of different subfamilies. Group I members contain five to nine conserved motifs, while Group II members only contained three conserved motifs, i.e., Motif 1, Motif 2, and Motif 3. Besides, Motif 8 and Motif 9 were only found in Group III members. The result of SMART analysis showed that SRS family contains a conserved domain, tentatively named Domain of unknown function 702 (DUF702), as well as several low complexity regions. Except for AtSRS8, all members of Group I and Group II contained DUF702 domain composed by Motif 1, Motif 2, and Motif 3, and a few of them also contained Motifs 6 and Motif 7. Neither *Arabidopsis thaliana* nor tomato Group III members contain the typical DUF702 domain .

In order to explain the protein structural differences among subfamilies, their protein sequences were further analyzed. In order to display conserved domains, multiple sequence alignment of the SRS protein sequences of *Arabidopsis thaliana* and tomato was performed (Fig. 2C). In contrast to the SMART analysis, multiple sequence alignment revealed all SRS proteins contained the RING-like zinc finger domain (CX_2_CX_7_CX_4_CX_2_C_2_X_6_C), which is characterized by en enrichment in cysteine residues. Furthermore, they all formed an α-helix at the same position, inside the RING-like zinc finger domain. However, in terms of specific types of amino acid residues, members of Group III were distinctly different from members of Group I and Group II. Looking back at motif analysis, we could find that Motif 1 and Motif 9 both contain the RING-like zinc finger domain, and Motif 2 contains the IXGH domain (Fig. 2B). In summary, although there are great differences in amino acid residues between members of Group III and members of Group I and Group II, they all have a conserved RING-like zinc finger domain.

### Collinearity analysis and duplication events among *SlSRS* genes

**Fig. 3:**
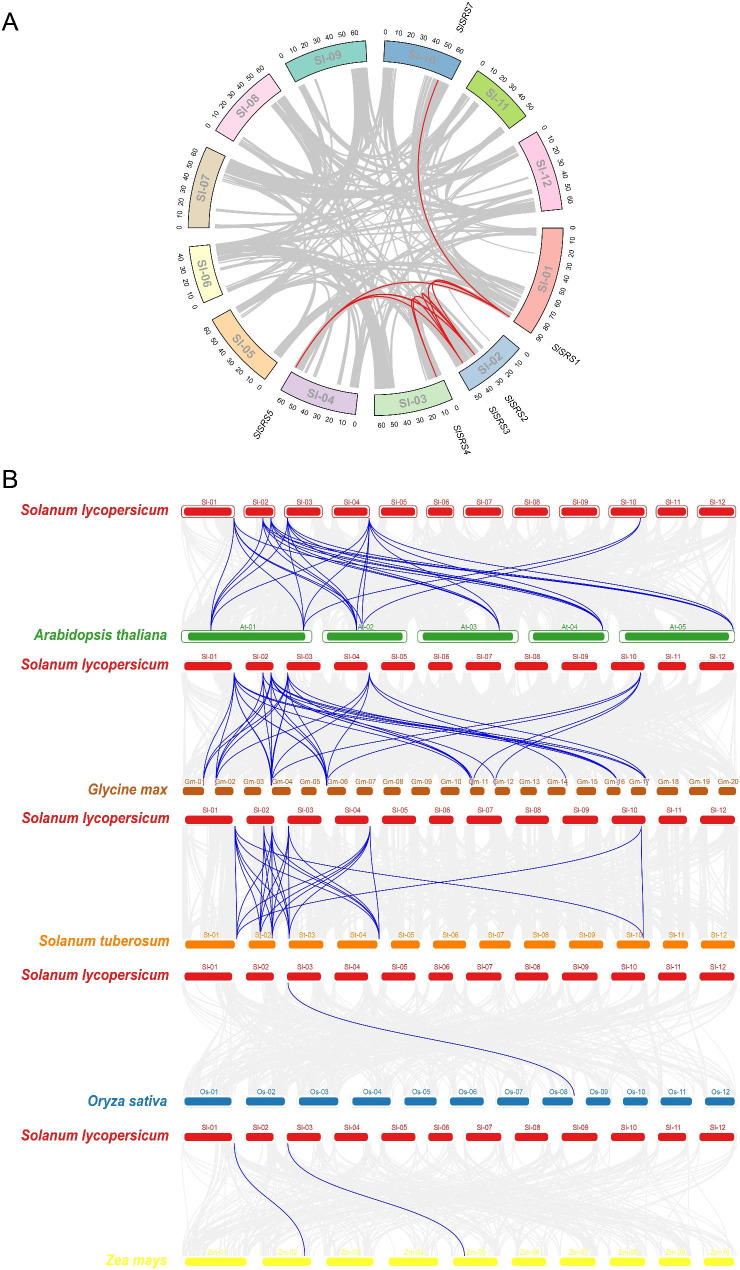
Collinearity analysis of *SlSRS* genes. **A**, Chromosomal localization and intra-species collinearity analysis of *SRS* genes in *Solanum lycopersicum* genome. Grgy lines indicate collinearity relationships. Red lines indicate collinearity relationships among *SRS* genes. **B**, Interspecies collinearity analysis of *SRS* genes among *Solanum lycopersicum*, *Arabidopsis thaliana*, *Glycine max*, *Solanum tuberosum*, *Oryza sativa*, and *Zea mays*. Grgy lines indicate collinearity relationships. Blue lines indicate collinearity relationships among *SRS* genes

Collinearity analysis is helpful to reveal the clues of *SRS* gene family evolution along with the process of species differentiation and formation. Gene duplication events lead to the expansion of the genome, and the subsequent functional differentiation of duplicates is regarded as the accelerator of evolution, which promotes the differentiation and formation of species. In *Solanum lycopersicum* genome, eight *SlSRS* genes were distributed on seven of twelve chromosomes, among which *SlSRS2* and *SlSRS3* were located together on chromosome 2 (Fig. S1). As shown in Fig. 3A, a total of nine *SlSRS* gene pairs with collinear relationships have been identified. Interestingly, these collinearity relationships only existed among the members of Group I, and all six members of Group I were generated from whole genome or segmental duplication events. In opposite, *SlSRS6* (Group III) and *SlSRS8* (Group II) are singleton genes. Subsequently, we calculated the non-synonymous (Ka) and synonymous (Ks) substitution of the collinear *SlSRS* gene pairs and used their ratios to evaluate the selection pressure during genome evolution. As shown in Table 2, there are nine gene pairs with collinearity relationships among 6 *SlSRS* genes belonging to Group I. The Ka//Ks ratios between them are all much less than 1, indicating that they have undergone purifying selection during their evolutionary history.

**Table 2:** Ks, Ka, and Ka/Ks values calculated for *SRS* gene pairs in the *Solanum lycopersicum* genome

Gene pairs	Ka	Ks	Ka/Ks radio
*SlSRS1-SlSRS3*	0.32	1.84	0.17
*SlSRS1-SlSRS2*	0.35	1.14	0.30
*SlSRS1-SlSRS5*	0.36	1.61	0.22
*SlSRS1-SlSRS7*	0.20	0.60	0.34
*SlSRS2-SlSRS3*	0.20	0.99	0.20
*SlSRS2-SlSRS4*	0.27	0.76	0.36
*SlSRS3-SlSRS4*	0.24	0.93	0.26
*SlSRS3-SlSRS5*	0.34	2.49	0.14
*SlSRS2-SlSRS5*	0.33	1.94	0.17

To further investigate the homologous genes of *SlSRS* genes in other species, collinearity analysis was performed between *Solanum lycopersicum* and dicotyledons *Arabidopsis thaliana* and *Glycine max*, *Solanaceous* plant *Solanum tuberosum*, monocots *Oryza sativa* and *Zea mays*, and primitive plants *Marchantia polymorpha* and *Physcomitrella patens*. It deserves mentioning that primitive plants and tomato do not share any collinear genes, probably due to their distant genetic relationships. Collinear *SRS* gene pairs were found among tomato, dicotyledons and monocotyledons, but they were all members of Group I. The results of collinearity analysis between tomato and dicotyledons are significantly different to and collinearity analysis between tomato and monocotyledons. In tomato, 30, 38 and 26 collinear gene pairs are found with *Arabidopsis thaliana*, *Glycine max* and *Solanum tuberosum*, respectively, and all six members of Group I have collinear relationships with multiple *SRS* genes from other species. In contrast, there are only one or two collinear gene pairs among tomato with *Oryza sativa* and *Zea mays*. Interestingly, these few collinearity pairs still exist only among members of Group I (Fig. 3B).

### *Cis*-acting elements in the promoter region of *SlSRS* genes

**Fig. 4:**
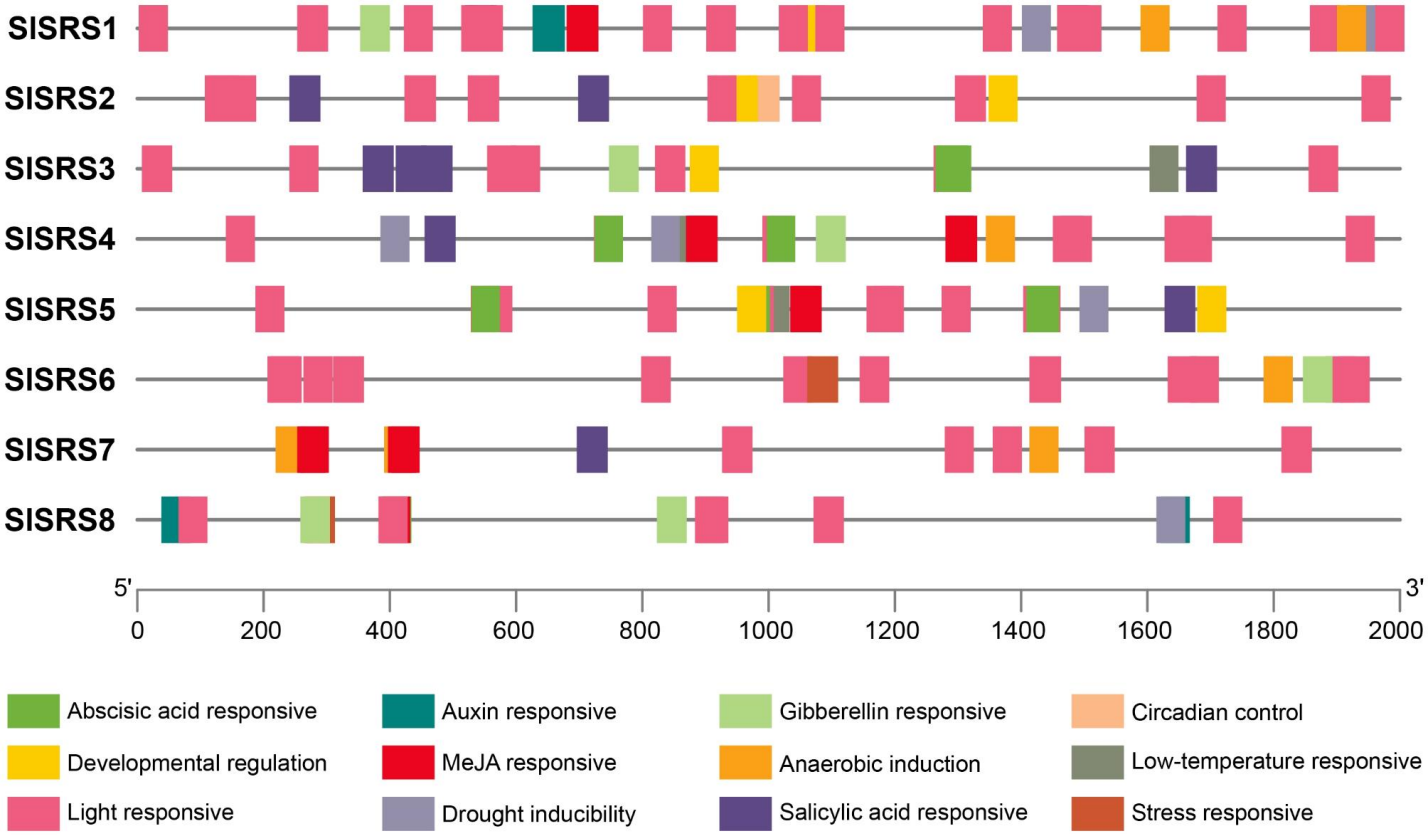
The *cis*-elements distributed in the promoters of *SlSRS* genes. Rectangles of different colors represent *cis*-elements with different functions

*Cis*-acting elements interact with TFs to activate gene expression in specific time, space, and conditions. Therefore, predicting *cis*-acting elements is instructive and enlightening for the study of gene function. In this study, the prediction of *cis*-acting elements was carried out in the promoter region of 8 *SlSRS* genes (2000 bp upstream of the 5’ UTR), and 34 *cis*-acting elements were identified. Their position and other detailed information were displayed in Fig. 4 and Table S1. As conserved promoter transcription regulatory elements, CAAT-box and TATA-box were also found in the core promoter areas of *SlSRS* genes. The other 32 elements could be divided into four categories according to their putative function: light responsive, stress responsive, hormone responsive and developmental regulation. Most of them were considered to be involved in light response, including 3-AF1 binding site, ACE, AE-box, ATCT-motif, Box 4, Box II, chs-CMA1a, circadian, GA-motif, GATA-motif, G-Box, GT1-motif, I-box, LAMP-element, MRE, TCCC-motif, and TCT-motif. In addition, ARE, LTR, MBS, and TC-rich repeats might be involved in responding to drought stress and low temperature stress. The response of auxin, gibberellin, MeJA and other hormones include ABRE, AuxRE, CGTCA-motif, GARE-motif, P-box, TATC-box, TCA-element, TGACG-motif, and TGA-element. Only two *cis*-acting elements were involved in the developmental regulation of plant tissues and organs: CAT-box, HD-Zip 1.

### Subcellular localization and transactivation activity analysis of SlSRS proteins

**Fig. 5:**
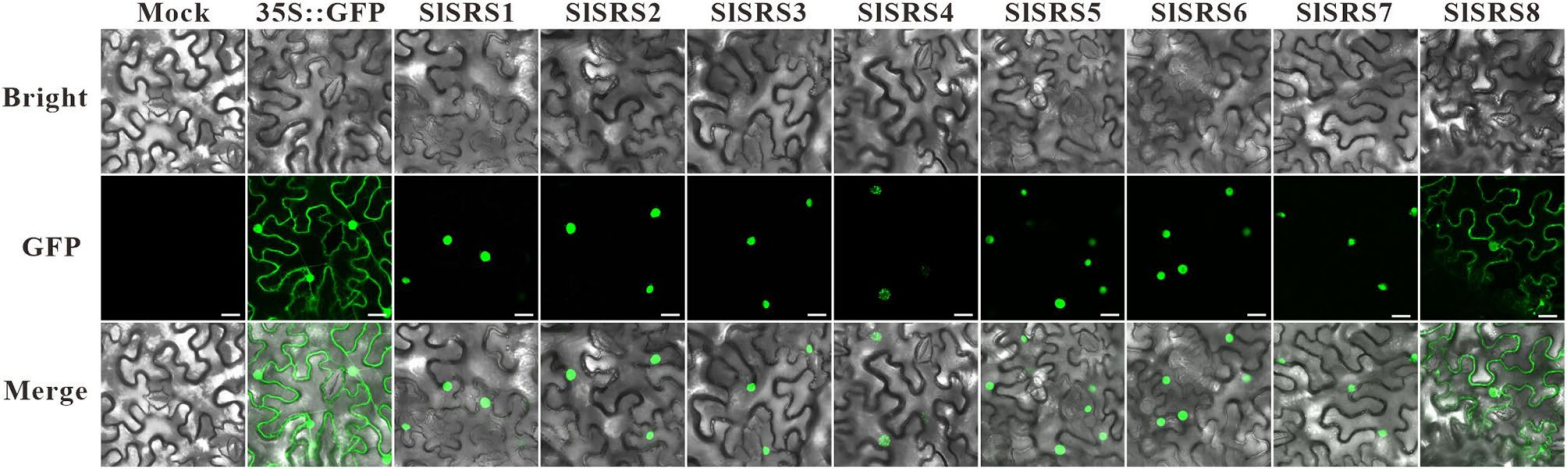
Subcellular localization analysis of SlSRS proteins. The SlSRS proteins fused with GFP was transiently expressed in tobacco (*Nicotiana benthamiana*) leaf cells to observe the subcellular localization through the laser scanning confocal microscope. Bars = 25 μm

Because nuclear import of transcription factors is instrumental to their transcriptional activity, we investigated the subcellular localization of SlSRS proteins. The SlSRS proteins fused with green fluorescent protein (GFP) was transiently expressed in tobacco (*Nicotiana benthamiana*) leaf cells to determine the subcellular localization. As shown in Fig. 5, except for SlSRS8, the fluorescent signal of other SlSRS fusion proteins is restricted to the nucleus, implying that 7 SlSRS proteins are all located in the nucleus. As for SlSRS8, the fluorescence signal is found mainly in the nucleus but also extends to the membrane. This phenomenon is not uncommon for transcription factors [30], and such results suggest that SlSRS8 might undergo important regulation at the post-translational level.

**Fig. 6:**
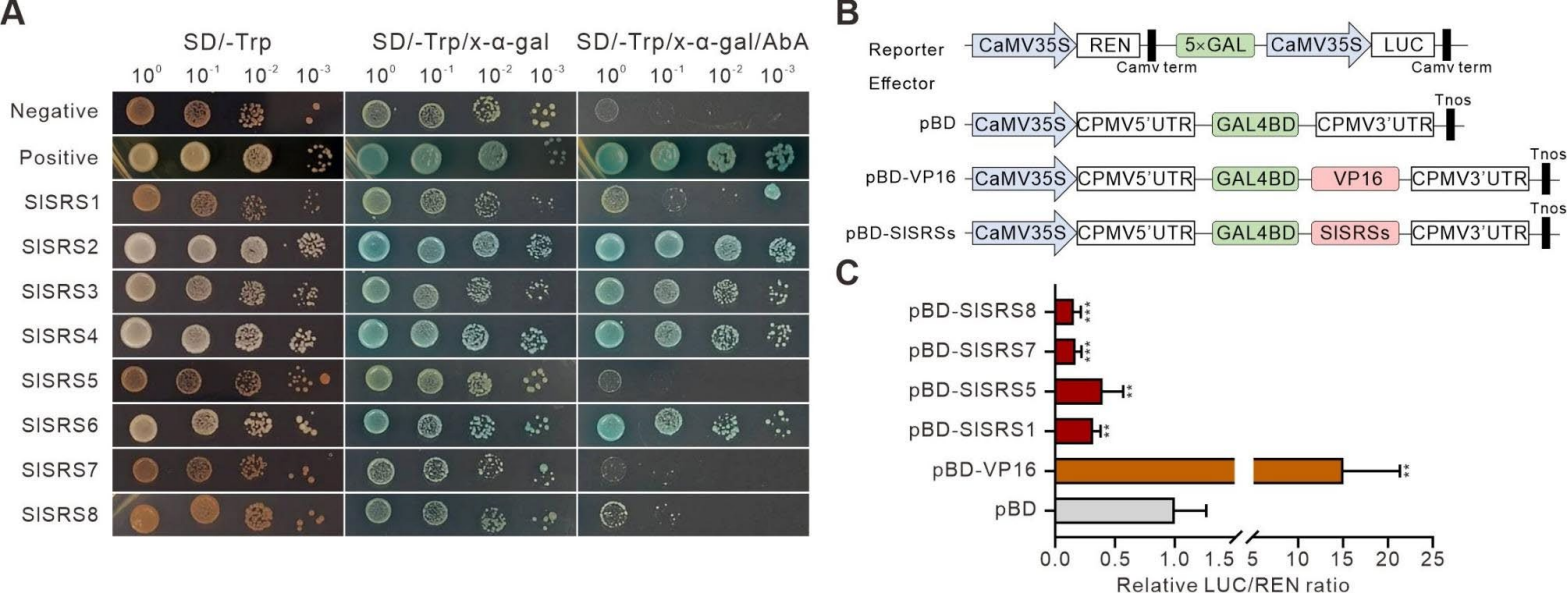
Transactivation activity analysis of SlSRS proteins. **A**, Transcriptional activation analysis of SlSRS proteins using yeast expression system. *SlSRS* genes coding sequence were inserted into pGBKT7 vector and then transformed into Y2H Gold yeast cells. The transformed yeast cells were spread on SD/-Trp, SD/-Trp/X-α-Gal or SD/-Trp/X-α-Gal/AbA medium to screen positive transformants. **B**, Structural schematic diagram of reporter and effectors vectors used for the dual luciferase assay. *SlSRS* genes coding sequence were amplified and cloned into the GAL4BD vector to generate the effectors. VP16 is used as the positive control. **C**, Transcriptional activation activity analysis of SlSRS1, SlSRS5, SlSRS7 and SlSRS8 by the dual luciferase assay. The LUC/REN ratio of the empty pBD vector was regarded as calibrator (set as 1). Each column represented the mean values of at least six biological replicates, and error bars represent the standard error values. The significant differences were indicated by asterisk (**p < 0.01, ***p < 0.001)

To further understand the functional characteristics of SlSRS proteins, the GAL4-responsive reporter system in yeast was performed to test the transcriptional activation activity of SlSRS proteins and results are shown in Fig. 6A. The full-length coding sequence of the *SlSRS* genes was inserted into the pGBKT7 vector and then transformed into Y2H Gold yeast cells. The transformed yeast cells were spread on SD/-Trp, SD/-Trp/X-α-Gal or SD/-Trp/X-α-Gal/AbA medium to screen positive transformants. Yeast cells harboring pGBKT7-SlSRS2, pGBKT7-SlSRS3, pGBKT7-SlSRS4, pGBKT7-SlSRS6 or the positive control (pGBKT7-53) hydrolyzed colorless X-α-Gal to the blue end product and survived from AbA screening, whereas yeast with pGBKT7-SlSRS1, pGBKT7-SlSRS5, pGBKT7-SlSRS7, pGBKT7-SlSRS8 and negative control (empty vector pGBKT7) did not, indicating that SlSRS2, SlSRS3, SlSRS4 and SlSRS6 proteins had transcriptional activation activity. The rest of four SlSRS proteins, SlSRS1, SlSRS5, SlSRS7 and SlSRS8, showed no transcriptional activation activity and were further tested for potential to act as transcriptional repressors using the dual-luciferase system. The full length of coding sequences of these four genes was amplified and cloned into the GAL4BD vector to generate the effectors (Fig. 6B). VP16 (a strong transcriptional activator) was used as the positive control. As expected, after transient co-expression of effectors and reporters in tobacco, the LUC/REN ratio of pBD-SlSRS1, pBD-SlSRS5, pBD-SlSRS7 and pBD-SlSRS8 were significantly lower than the pBD alone (negative control) (Fig. 6C). Considering that these SlSRS proteins show no transcriptional activation activity in yeast, we demonstrated that SlSRS1, SlSRS5, SlSRS7 and SlSRS8 function as the transcription repressors.

### Expression profiles of *SlSRS* genes in various tomato organs of different development stages

**Fig. 7:**
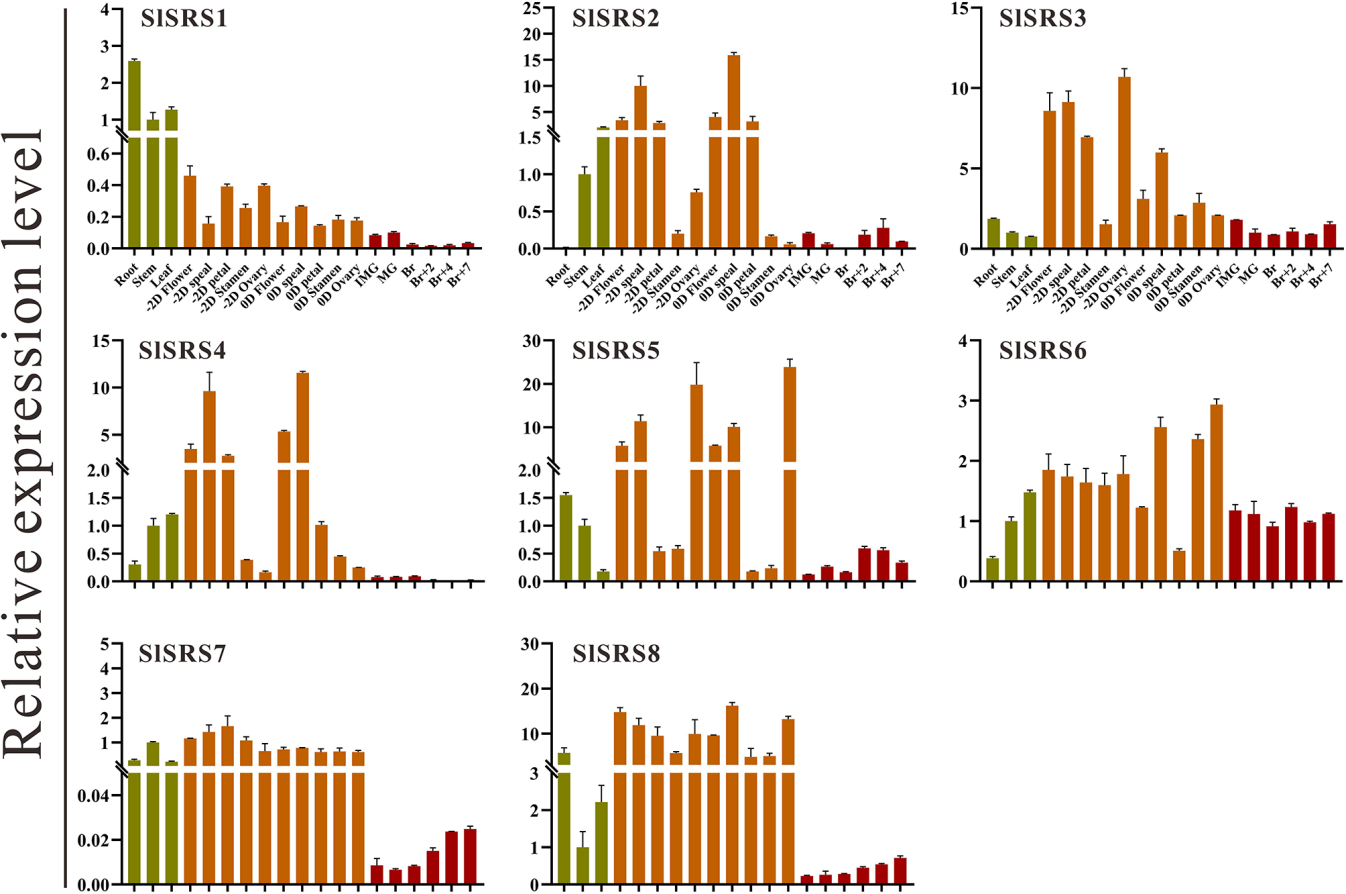
Expression profiles of *SlSRS* genes in various tomato organs. The expression levels of *SlSRS* genes in in vegetative organs, flowers organs at anthesis and 2 days before anthesis and fruit at different development stages was analyzed by qRT-PCR. -2D and 0D represent 2 days before anthesis and 0 day after anthesis, respectively. IMG: immature green fruit, MG: mature green. Br: breaker. Br2/4/7, 2/4/7 days after breaker. The expression level of *SlSRS* genes in stem was set as 1. Each column represented the mean values of three independent biological replicates, and error bars represent the standard error values

The spatiotemporal expression profiles study contributes to investigating the potential function of the *SlSRS* genes during the different developmental processes of tomato. Therefore, the expression patterns of *SlSRS* genes in vegetative organs (root, stem, leaf), flowerorgans at anthesis and 2 days before anthesis (sepal, petal, stamen and ovary) and fruit at different development stages (immature green, mature green, breaker, 2 days, 4 days and 7 days after breaker) were detected by qRT-PCR. As shown in Fig. 7, in general, except for *SlSRS1*, which shows high transcript accumulation in vegetative organs, most *SlSRS* genes expressed ubiquitously in all examined flower organs, implying that *SlSRS* genes likely have important functions in the regulation of tomato flower organs development. The relatively lower expression level of the *SlSRS* genes was observed in fruits compared to the vegetative organs and the flower organs. However, the expression level of *SlSRS5*, *SlSRS7*, and *SlSRS8* were gradually increased during fruit ripening, suggesting that they might play a role in fruit ripening. The highest transcript accumulation of *SlSRS5* was observed in ovary at anthesis and 2 days before anthesis, suggesting that *SlSRS5* might be involved in regulating ovary development or fruit set. Interestingly, *SlSRS7* and *SlSRS8* showed similar spatial-temporal expression patterns, implying that *SlSRS7* and *SlSRS8* might have functional redundancy or synergistic effects in regulating tomato plant development.

### Expression profiles of *SlSRS* genes in response to plant hormone

**Fig. 8:**
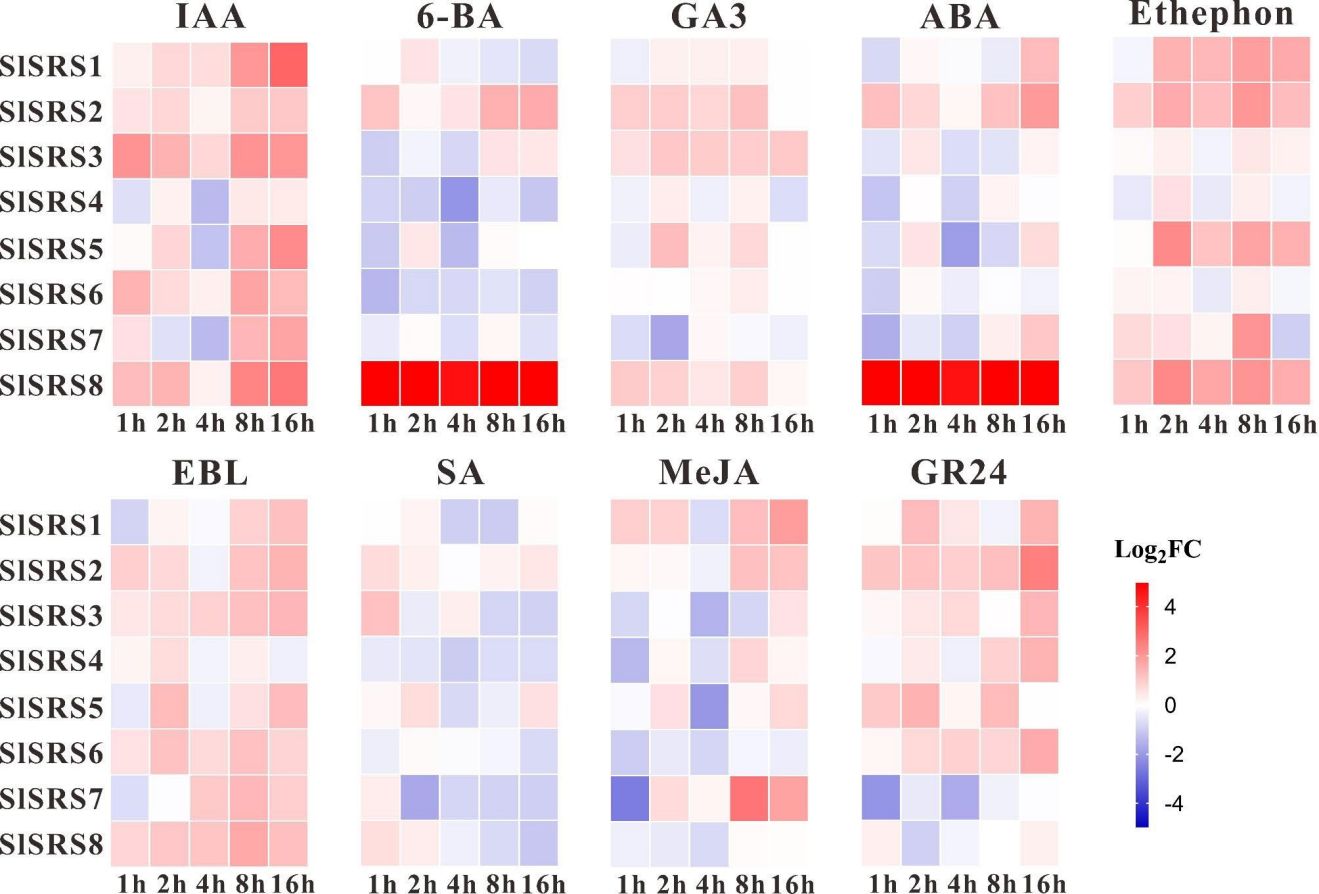
Expression profiles of *SlSRS* genes under various hormone treatments. The expression level of *SlSRS* genes under IAA, GA3, 6-BA, ethephon, EBL, SA, MeJA, ABA and GR24 treatments was analyzed by qRT-PCR. Tomato seedlings were treated with different hormones and sampled at 1 h, 2 h, 4 h, 8 and 16 h to test the responsiveness of *SlSRS* genes to different hormones. The data were converted to log2FC (FC, fold change) and the heat map was used to represent the responsiveness of the *SlSRS* genes. Blue and red represent downregulated and upregulated genes under different hormone treatments, respectively. Each time point represented the mean values of three independent biological replicates

The important role of plant hormones in the regulation of plant growth, development, and environmental adaptation has gradually attracted attention over the past decades. Understanding expression profiles of *SlSRS* genes under different plant hormone treatments could contribute to revealing the potential function of these genes. In this study, we analyzed the responsiveness of *SlSRS* genes to nine kinds of plant hormones or their analogues by qRT-PCR, including indole-3-acetic acid (IAA), Gibberellin A3 (GA3), 6-Benzylaminopurine (6-BA), ethephon, epi-brassinolide (EBL), salicylic acid (SA), methyl jasmonate (MeJA), Abscisic Acid (ABA) and strigolactone (GR24). Overall, the responsiveness of *SlSRS* genes to different hormones varied greatly, but all *SlSRS* genes could respond to at least four kinds of plant hormones. It is noteworthy, *SlSRS2*, *SlSRS5* and *SlSRS7* can respond to eight kinds of plant hormones (Fig. 8). All *SlSRS* genes could respond to auxin, of which *SlSRS1*, *SlSRS3*, *SlSRS5* and *SlSRS8* were strongly induced by auxin, suggesting that there was a potential connection between *SlSRS* genes and auxin signalling. In addition, ethephon, GR24, and EBL also could affect the expression of most *SlSRS* genes, while some *SlSRS* genes showed relative mild responsiveness to SA. Notably, *SlSRS* genes exhibited an opposite trend in response to some plant hormones. For example, 6-BA induced the expression of *SlSRS2* and *SlSRS8* while inhibiting *SlSRS4*, *SlSRS5* and *SlSRS6*. The expression of *SlSRS2* could be significantly induced by all plant hormones or their analogues except SA, suggesting that *SlSRS2* might play potential functions in multiple plant hormone signalling pathways. Interestingly, the transcript accumulation of *SlSRS8* was significantly increased under 6-BA and ABA treatment, indicating that *SlSRS8* might be involved in cytokinin and/or ABA mediated growth, development, and stress response of tomato plants. In conclusion, our results support the notion that *SlSRS* genes could respond to multiple hormonal signals. The analysis of hormone responsiveness of *SlSRS* genes provides a good theoretical reference for studying gene function.

### Expression profiles of *SlSRS* genes in response to stresses

**Fig. 9:**
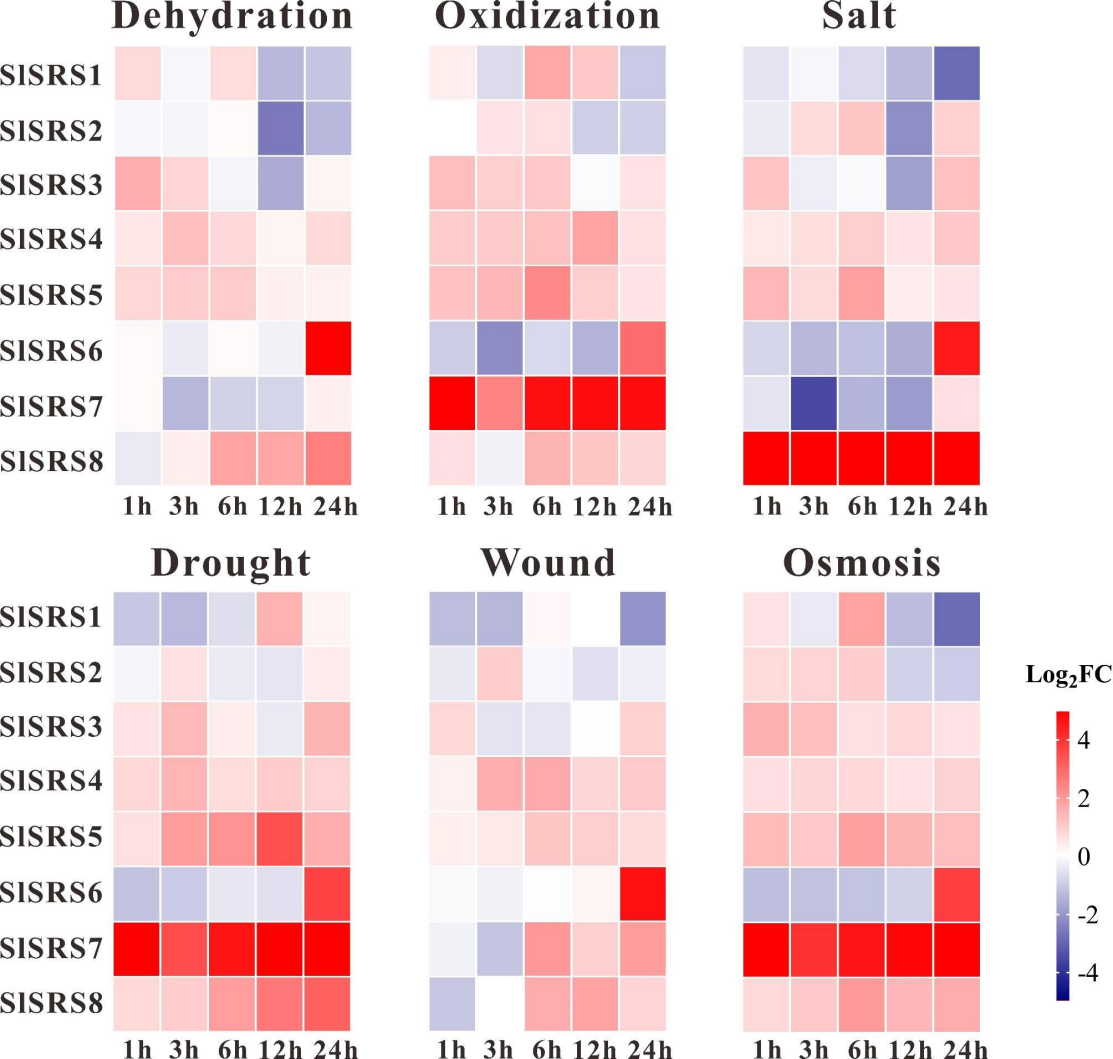
Expression profiles of *SlSRS* genes under various sterss treatments. The expression level of *SlSRS* genes under dehydrated (Dehydration), oxidative (MV), salt (NaCl), Droughty (PEG6000), injured (Wound) and osmotic (Mannitol)treatments was analyzed by qRT-PCR. One-month-old tomato plants were treated with different hormones and sampled at 1 h, 3 h, 6 h, 12 and 24 h to test the responsiveness of *SlSRS* genes to different stresses. The data were converted to log2FC (FC, fold change) and the heat map was used to represent the responsiveness of the *SlSRS* genes. Blue and red represent downregulated and upregulated genes under different stress treatments, respectively. Each time point represented the mean values of three independent biological replicates

To further investigate the potential responsiveness of *SlSRS* genes to various biotic and abiotic stresses, the transcriptional changes of the *SlSRS* genes under dehydrated (Dehydration), oxidative (MV), salt (NaCl), droughty (PEG6000), injured (Wound) and osmotic (Mannitol) stresses were assessed by qRT-PCR. In general, the expression of most *SlSRS* genes could be induced by these six stress treatments. Except for *SlSRS2*, the other *SlSRS* genes could respond to at least five kinds of stresses, of which *SlSRS1*, *SlSRS5*, *SlSRS6*, *SlSRS7* and *SlSRS8* could respond to all six kinds of stresses (Fig. 9). The transcript level of *SlSRS1* is inhibited under various stress treatments, indicating that *SlSRS1* might play a negative regulator of abiotic stress response. Besides, all the *SlSRS* genes were sensitive to dehydration and salt stresses, suggesting that *SlSRS* genes might contribute to plant adaptation to environmental stress. Notably, *SlSRS6* exhibited a unique expression pattern, and the expression of *SlSRS6* reached its highest peak at 24 h after various stress treatments. *SlSRS7* showed strong responsiveness to oxidation, drought and osmotic stress, and the transcript accumulation of *SlSRS8* was significantly increased under dehydration, salt, drought and osmotic treatment, implying that *SlSRS7* and *SlSRS8* genes could be used as the candidate genes to improve the stress resistance of tomato plant. Our results indicated that the *SlSRS* genes exhibited different responses and regulatory mechanisms under different abiotic stresses.

## Discussion

SRS is a small plant-specific TF family. Among dicotyledonous, *Arabidopsis thaliana* has 11 SRS TFs [2, 25], *Gossypium hirsutum* has 26 members [26], and *Glycine max* has 21 members [25]. Among monocotyledonous plants, *Oryza sativa* has 9 [29], and *Zea mays* has 5 members. As far, only *Arabidopsis* SRS TFs have been comprehensively and deeply studied. In addition, only a few species’ SRS families have been completed at genome-wide identification, and data on functional and molecular mechanism analysis are still sparsed. As a model plant for studying fleshy fruits, it is necessary to identify and characterize the SlSRS family of tomato. This helps to uncover potential TFs related to fruit development and quality control, which is important for breeding. In this study, 8 SRS TFs were identified from tomato, which could be divided into three subfamilies with different characteristics.

All SRS TFs contain an extremely conservative RING-like zinc finger domain (CX_2_CX_7_CX_4_CX_2_C_2_X_6_C), which is the structural basis for their transcriptional functions. This is a RING domain rich in acidic Cys and His residues with transcriptional activity [2]. The RING domain can form two cross-brace finger loops to couple two zinc atoms [31]. The IXGH domain, which also have transcriptional activity, is only present in members of Group I and Group II, which is one of the characteristics that distinguishes Group III from the other two subfamilies [8, 25]. Eklund et al. reported that IXGH domain of AtSTY1 could mediate intrafamily homo-/heterodimerization [8]. In *Arabidopsis thaliana*, AtLRP1 has been reported to interact with AtSHI, AtSTY1, AtSRS3, AtSRS6 and AtSRS7 proteins of the SRS family [12]. Further studies and additional evidence are required to determine whether homo-/heterodimerizations are formed among SRS family members. In tomato, all 8 SRS TFs contain RING-like zinc finger domain. However, motif prediction and sequence analysis showed that SlSRS6 in Group III is different from other members. The RING-like zinc finger domain of SlSRS6 contains conserved cysteine residues, but the other amino acid residues that constitute the domain are different from other members, which also leads to the prediction of two kinds of RING-like zinc finger domain as Motif 1 and Motif 9, respectively. In addition, SlSRS6, like *Arabidopsis thaliana* AtSRS11, has no IXGH domain that is present in most SRS proteins, suggesting that SlSRS6 has different functions from other members.

Gene duplication events play an important role in genomic expansion and gene functional diversity [32]. Among *SlSRS* genes, tandem duplication events were not found, but 6 segmental duplications were detected in the tomato chromosomes (Fig. 3A). There are 9 pairs of collinearity relationships among 6 Group I members with segmental duplication. The results mean that those *SlSRS g*enes might be generated by gene duplication events. *SlSRS* has more homologous genes in dicotyledon than monocotyledon. SRS TF family has distinct differentiation between dicotyledon and monocotyledon. This differentiation is not only observed in quantity, but also in sequence structure. Several grass-specific SRS proteins have been identified in monocotyledon, such as HvLks2 [4] and HvVRS2 [5] in barley. These grass-specific SRS proteins have no homologous genes in *Arabidopsis thaliana* [4]. This indicates that SRS TF family evolved separately in dicotyledon and monocotyledon.

SRS TFs regulate various plant hormones biosynthesis and signal transduction, and directly participate in the development of multiple plant organs. *AtLRP1*, the first reported SRS gene, is specifically expressed in *Arabidopsis thaliana* LRs [7]. It has been reported that auxin and histone deacetylation influence *AtLRP1* expression to regulate root development [12]. The maize homologous gene *ZmLRP1* also has a similar mechanism [22]. The inhibition of *ZmLRP1* expression is directly regulated by ZmRUM1. AtSTY1 and AtSTY2 function are redundant, and promote style and stigma formation and influence vascular development during *Arabidopsis* gynoecium development [13]. Furthermore, AtSTY1 could activate *AtYUC4* expression and induce the transcription of other auxin-related genes to regulate auxin levels and auxin biosynthesis rates [8]. In this manner, AtSTY1 is involved in leaf and flower development [14–16]. Another SRS TF AtSRS5 acts downstream of auxin [19]. AtSRS5 is a negative regulator for LR formation by repressing the expression of *LBD16/29*. AtARF7/19 could directly bind to the promoter of *AtSRS5* and inhibit its expression, which is induced by auxin. In tomato, *SlSRS1*, as homologous gene of *AtSRS5*, also contains an AuxRE element in its promoter that is specifically bound by ARF TF (Fig. 4). *SlSRS1* expression was up-regulated after IAA treatment, and was specifically expressed in roots (Fig. 7). Notably, SlSRS1 displays a transcriptional inhibitory activity like AtSRS5, which means it may be a negative regulator (Fig. 6). This indicates that SlSRS1 may be induced by auxin and regulate root development. *SlSRS8* is homologous to *AtLRP1* and contains two auxin-responsive elements (TGA-elements) in its promoter (Fig. 4). Similarly, *SlSRS8* expression was up-regulated by auxin and mainly expressed in floral organs. Interestingly, the expression of *SlSRS8* was up-regulated during fruit development (Fig. 8). *SlSRS8* may be involved in tomato fruit development and ripening.

In addition to auxin, SRS TFs are also involved in the regulation of metabolism and response of multiple plant hormones. SHI acts as a negative regulator of GA responses by specifically suppressing expression of a GA induced gene [1, 2]. In *Populus*, PtSHI regulate GA metabolism and ⁄or signalling and indirectly influences cytokinin metabolism [21]. Grass-specific SRS TFs of HvVRS2 was reported to regulate inflorescence patterning during spike development by maintaining multiple hormonal homeostasis and gradients, including auxin, CK and GA [5]. In this study, the *cis*-acting elements production revealed that several *SlSRS* genes contain plant hormone responsive elements on their promoters, such as abscisic acid, auxin, GA, MeJA, and salicylic acid (Fig. 4). All *SlSRSs* could respond to at least four kinds of plant hormone, indicating their pleiotropic effects in hormone regulation (Fig. 8).

The whole life cycle of plants is accompanied by interaction with external environmental factors, which can be biotic and/or abiotic. In terms of biotic factors, the expression of *AtSRS5* is significantly increased due to pathogen induction [17], suggesting a potential role for AtSRS5 in the regulation of plant immune responses. On the other hand, the expression of many *SRS* genes in different species are induced by abiotic factors. GmSRS18 of *Glycine max* and GhSRS21 of *Gossypium hirsutum* negatively regulate salt tolerance [25, 26]. Several *SRS* genes in *Medicago sativa* and *Melilotus albus* can respond to low temperature and salt stress [23, 24]. In this study, except for *SlSRS2*, other *SlSRSs* contain at least one kind of stress response element in their promoter (Fig. 4). Notably, all *SlSRSs* contain several light response elements in their promoter. Consistently, except for *SlSRS2*, other *SlSRS* members could respond to at least five kinds of stresses (Fig. 9), implying that SlSRS TFs may be also involved in the response of plants against abiotic stress in tomato. Furthermore, *SlSRS7* and *SlSRS8* have strong responses to various of hormones and stresses, which deserve further study.

## Conclusions

In summary, eight SlSRS TF family members were identified in *Solanum lycopersicum*. Their phylogenetic relationship, conserved motifs, conserved amino acid residues within characteristic domains, chromosomal location, gene duplication events, evolutional relationships and *cis*-elements were systematically analyzed. Moreover, the subcellular localization and transcriptional activity of SlSRS proteins were further investigated. In addition, the expression profiles of *SlSRS* genes in different tissues showed putative important function in tomato floral organ and fruit development. Furthermore, the critical regulatory roles were implied by the expression patterns of *SlSRS* genes in response to plant hormones and stresses. Overall, our results lay an important foundation for further functional research of these *SlSRS* genes.

## Materials and methods

### Plant materials, hormone and stress treatments

In this study, tomato plants (*Solanum lycopersicum* cv. Micro-Tom) and tobacco plants (*Nicotiana benthamiana* L.) were cultured under the growth conditions of 16/8 h light/dark cycle, 25/20°C day/night temperature and 60% relative humidity. Various organs of tomato plants at different developmental stages were collected in three biological replicates.

The hormone and stress treatments were carried out as described by Su et al. [33]. For hormone treatments, 12-day-old tomato seedlings were soaked into liquid MS/2 medium containing 20 µM IAA, 10 µM 6-BA, 20 µM GA3, 100 µM ABA, 20 µM ethephon, 0.5 µM EBL,20 µM SA, 50 µM MeJA and 5 µM GR24 respectively, together with liquid MS/2 medium without any hormones as control, and then incubated in the dark at 25 °C. After 1 h, 2 h, 4 h, 8 and 16 h respectively, samples were collected for subsequent test.

For stress treatments, one-month-old tomato plants were subjected to the droughty, osmotic, oxidative, salt, dehydrated, and injured stress. Tomato plants were soaked into solutions containing 20% (m/v) PEG6000, 100 mM mannitol, 150 µM methyl viologen (MV) and 200 mM NaCl, respectively, for droughty, osmotic, oxidative and salt stress treatments, and then cultured at standard conditions. Tomato plants with dehydrated stress treatment were removed the soil and cleaned by water, then placed on the filter papers and naturally dried at room temperature. Clean tweezers were used to pierce tomato leaves at the same position for injured stress treatment. The control were well-watered tomato plants. After for 1 h, 3 h, 6 h, 12 and 24 h, leaves at the same position of three individual plants were harvested as one sample. All treated samples were set up in three biological replicates and immediately frozen with liquid nitrogen after collection and stored at −80 °C.

### Identification and phylogenetic analysis of SRS TF in *Solanum lycopersicum*

Genomic data were obtained from Phytozome (https://phytozome-next.jgi.doe.gov/) [34], including *Solanum lycopersicum* ITAG4.0 [35], *Arabidopsis thaliana* TAIR10 [36], *Glycine max* Wm82.a4.v1 [37], *Solanum tuberosum* v3.0 [38], *Oryza sativa* v7.0 [39], *Zea mays* Zm-B73-REFERENCE-NAM-5.0 [40], *Marchantia polymorpha* v3.1 [41] and *Physcomitrella patens* v3 [42]. The hidden Markov model (HHM) of Domain of Unknown Function 702 (DUF702) (PF01542) was downloaded from Pfam database (InterPro, https://www.ebi.ac.uk/interpro/) [43], which was used to search for the putative tomato SRS proteins on HMMER web server (http://hmmer.org/) [44]. *Arabidopsis* and rice SRS protein sequences were used as queries to carry out BLASTP search against tomato proteins. The sequences with *E*-value < 1e^10^ were selected for further analysis. Subsequently, all the candidate protein sequences were further examined by Conserved Domain Database (NCBI-CDD) (https://www.ncbi.nlm.nih.gov/Structure/cdd/wrpsb.cgi/) [45]. In addition, ProtParam tool of Expasy web server (https://web.expasy.org/protparam/) [28] was used to analyze the physical and chemical parameters of the tomato SRS proteins.

MUSCLE was used for multiple sequence alignment of the given protein sequences. After being processed by trimAL, the alignment was used to construct the phylogenetic tree by MEGA X [46] with the neighbor-joining (NJ) method with the bootstrap test replicated 1,000 times, the Poisson model and pairwise deletion. Finally, Interactive Life Tree (iTOL, https://itol.embl.de/index.shtml/) [47] was used for the phylogenetic tree visualization.

### Motif prediction, conserved domains, and secondary structure prediction

SRS protein sequences from tomato and *Arabidopsis thaliana* were used for subsequent analysis. Their motif structures were predicted using Motif-based sequence analysis tools (MEME) web server (https://meme-suite.org/meme/tools/meme) [48], using the default parameter, except that the maximum number of motifs was set to 10. Simple Modular Architecture Research Tool (SMART) (https://smart.embl.de) [49] was used to analyze conserved domains of sequences. JPred4 of Jalview [50] was used to predict the protein secondary structure of these sequences.

### Chromosomal location, collinearity analysis and selective pressure analysis

Advanced Circos of TBtools [51] was used for chromosomal localization and intraspecies collinearity analysis. One Step MCScanX of TBtools was used for interspecies collinearity analysis. The non-synonymous (Ka) and synonymous (Ks) substitution ratios between collinear gene pairs were calculated by the Simple Ka/Ks Calculator of TBtools.

### Prediction of *cis*-acting elements in promoter region

The 2000-bp putative promoter regions of the *SlSRS* genes were obtained by GXF Sequences Extract of TBtools. Next, these sequences were uploaded to the PlantCARE web server (https://bioinformatics.psb.ugent.be/webtools/plantcare/html/) [52] for analysis.

### Subcellular localization of SlSRS proteins

The full-length coding sequence of *SlSRS* genes without stop codon was fused into pCXDG-GFP vector. Then the fusion plasmid was transformed into *Agrobacterium tumefaciens* (GV3101). Transient expression of fusion SlSRS-GFP protein was carried out in leaves of one-month-old tobacco. Three days after infection, the green fluorescence was observed by laser scanning confocal microscope (Leica TCS SP8, Germany).

### Transactivation activity analysis in yeast

The transcriptional activity of SlSRS proteins were analyzed by the GAL4-responsive reporter system in yeast. The open reading frame (ORFs) of *SlSRS* genes were amplified and ligated into pGBKT7-GAL4BD plasmid which were subsequently transformed into Y2H Gold yeast cells. The yeast was cultured in the SD/Trp medium plate. X-α-gal was used for the identification of the α -galactosidase activity of the transformant, and Aureobasidin A (AbA, Clontech, USA) was used for expression screening.

### Dual-luciferase assay

The ORFs of *SlSRS1*, *SlSRS5*, *SlSRS7* and *SlSRS8* were amplified and ligated into pEAQ-GAL4BD plasmid as effector plasmid, with VP16 as a positive control. The double-reporter vector pGreenII 0800-LUC was used as the reporter. The renilla luciferase (REN) driven by CaMV35S was used as the internal control. *Agrobacterium tumefaciens* (GV3101) with the effector and reporter respectively were co-infected the one-month-old tobacco leaf with the ratio of effector: reporter = 9:1. After 3 days, the activities of LUC and REN were detected by Dual-Luciferase Reporter Assay System (Promega, USA). Each combination contained six biological replicates. Finally, the transcriptional activation activity was evaluated by LUC/REN ratio.

### RNA isolation, cDNA synthesis and quantitative real-time PCR analysis

Total RNA was extracted using RNAprep Pure Plant Kit (Tiangen Biotech, China) following the manufacturer’s instructions. The first strand of cDNA was synthesized by PrimeScript™ RT reagent Kit with gDNA Eraser (Perfect Real Time) (Takara, Japan). TB Green® Premix Ex Taq™ II (Tli RNaseH Plus) (Takara, Japan) was used to perform qRT-PCR on the CFX96 Touch™ Real-Time PCR Detection System (BIO-RAD, USA). Two microliter 5-fold diluted cDNA was used in each reaction. Finally, the relative expression was calculated by the 2^−ΔΔCt^ method.

## Electronic supplementary material

Below is the link to the electronic supplementary material.

Supplementary Material 1

## Data Availability

Genomic data were collected from Phytozome (https://phytozome-next.jgi.doe.gov/), including *Solanum lycopersicum* ITAG4.0, *Arabidopsis thaliana* TAIR10, *Glycine max* Wm82.a4.v1, *Solanum tuberosum* v3.0, *Oryza sativa* v7.0, *Zea mays* Zm-B73-REFERENCE-NAM-5.0. *Cis*-elements were obtained from PlantCARE database (http://bioinformatics.psb.ugent.be/webtools/plantcare/html/). The SRS family expression data were generated by qRT-PCR and were available from the corresponding authors when needed. All other data supporting the results are included within the article and its Additional files.

## Abbreviations

ABA: Abscisic Acid
AbA: Aureobasidin A
BA: Benzylaminopurine
CDD: Conserved Domain Database
DUF702: Domain of Unknown Function702
EBL: Epi-brassinolide
EOT1: Expression of Terpenoids1
GA: Gibberellic acid
GFP: Green fluorescent protein
HHM: Hidden Markov model
IAA: Indole-3-acetic acid
IPA1: IDEAL PLANT ARCHITECTURE1
JA: Jasmonic acid
Ka: Non-synonymous
Ks: Synonymous
Lks2: Short awn2
LR: Lateral root
LRP1: Lateral root primordium1
MeJA: Methyl jasmonate
MEME: Motif-based sequence analysis tools
NJ: Neighbor-joining
ORF: Open reading frame
REN: Renilla luciferase
RUM1: ROOTLESS WITH UNDETECTABLE MERISTEM1
SA: Salicylic acid
SHI: SHORT INTERNODES
SMART: Simple Modular Architecture Research Tool
SRS: SHI RELATED SEQUENCE
STY1: STYLISH1
TF: Transcription factor
TGA-elements: Two auxin-responsive elements
TPS5: TERPENE SYNTHASE5
VRS2: Six-rowed spike2
YUC4: YUCCA4
Y2H: Yeast Two-Hybrid
