# Correction: Identification of SRS transcription factor family in *Solanum lycopersicum*, and functional characterization of their responses to hormones and abiotic stresses

**DOI:** 10.1186/s12870-025-06778-2

**Published:** 2025-05-31

**Authors:** Wang Lu, Yan Wang, Yuan Shi, Qin Liang, Xiangyin Lu, Deding Su, Xin Xu, Julien Pirrello, Ying Gao, Baowen Huang, Zhengguo Li

**Affiliations:** 1 Key Laboratory of Plant Hormones and Development Regulation of Chongqing, School of Life Sciences, Chongqing University, Chongqing, 401331 China; 2 Center of Plant Functional Genomics, Institute of Advanced Interdisciplinary Studies, Chongqing University, Chongqing, 401331 China; 3 Laboratory of Plant Science Research, Fruit Genomics and Biotechnology, UMR5546, University of Toulouse, CNRS, UPS, Toulouse-NP, Toulouse, France

Correction: *BMC Plant Biol* **23**, 495 (2023)

10.1186/s12870-023-04506-2.

Following publication of the original article [1], the authors found an error in the published version of Fig. 5 where the graph of subcellular localization of SlSRS7 is not assembled correctly. The correct figure is presented below:

**Incorrect Fig.** 5

**Fig. 5:**
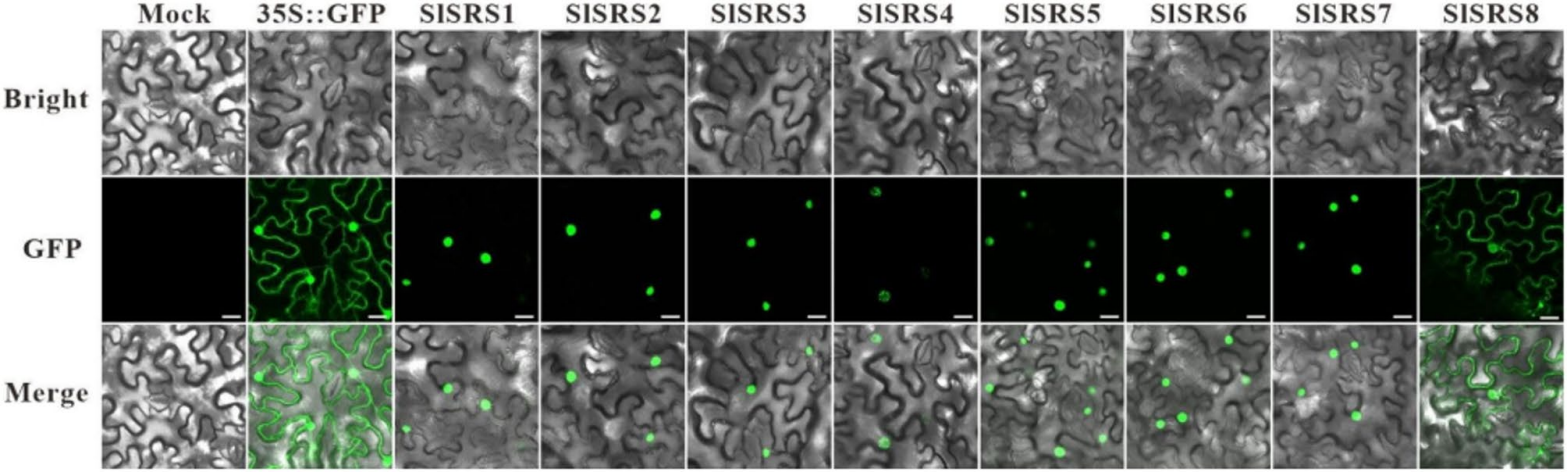
Subcellular localization analysis of SlSRS proteins. The SlSRS proteins fused with GFP was transiently expressed in tobacco (*Nicotiana benthamiana*) leaf cells to observe the subcellular localization through the laser scanning confocal microscope. Bars = 25 μm

**Correct Fig.** 5

**Fig. 5:**
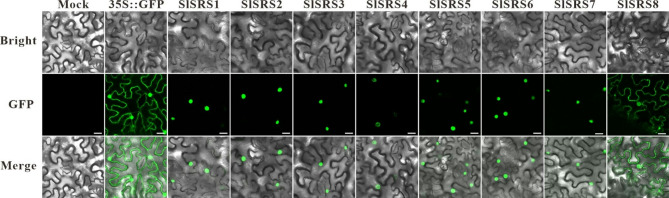
Subcellular localization analysis of SlSRS proteins. The SlSRS proteins fused with GFP was transiently expressed in tobacco (*Nicotiana benthamiana*) leaf cells to observe the subcellular localization through the laser scanning confocal microscope. Bars = 25 μm

The correction does not compromise the validity of the conclusions and the overall content of the article. The original article [1] has been updated.

## References

[1] Lu W, Wang Y, Shi Y, et al. Identification of SRS transcription factor family in *Solanum lycopersicum*, and functional characterization of their responses to hormones and abiotic stresses. BMC Plant Biol. 2023;23:495. 10.1186/s12870-023-04506-2. 37833639 10.1186/s12870-023-04506-2 PMC10576376

