# Supplementary material for: Identification of SRS transcription factor family in Solanum lycopersicum, and functional characterization of their responses to hormones and abiotic stresses
Source: BMC Plant Biol. 2023 Oct 14;23:495. doi: 10.1186/s12870-023-04506-2 (PMC10576376; doi:10.1186/s12870-023-04506-2)
Supplement: Supplementary file 1 — Supplementary Material 1 [file 12870_2023_4506_MOESM1_ESM.docx]

**Title:** Identification of SRS transcription factor family in *Solanum lycopersicum*, and functional characterization of their responses to hormones and abiotic stresses

Authors: Wang Lu^1, 2, †^, Yan Wang^1, 2, †^, Yuan Shi^1, 2, †^, Qin Liang^1, 2^, Xiangyin Lu^1, 2^, Deding Su^1, 2^, Xin Xu^1, 2^, Julien Pirrello^3^, Ying Gao^1, 2^, Baowen Huang^1, 2^ *, Zhengguo Li^1, 2^ *

^1^ Key Laboratory of Plant Hormones and Development Regulation of Chongqing, School of Life Sciences, Chongqing University, 401331 Chongqing, China.

^2^ Center of Plant Functional Genomics, Institute of Advanced Interdisciplinary Studies, Chongqing University, 401331 Chongqing, China.

^3^ 1Laboratoire de Recherche en Sciences Végétales — Génomique et Biotechnologie des Fruits —UMR5546, Université de Toulouse, CNRS, UPS, Toulouse-INP, Toulouse, France.

† These authors contributed equally to this work.

* **Corresponding authors:**

Baowen Huang; email, huangbaowen2009@163.com;

Zhengguo Li; email, [zhengguoli@cqu.edu.cn](mailto:zhengguoli@cqu.edu.cn); Telephone and fax, +86-23-65678902

**Authors’ email addresses:**

Wang Lu, [lwang4901@163.com](mailto:lwang4901@163.com)

Yan Wang, w120566y@163.com

Yuan Shi, [18306012148@163.com](mailto:18306012148@163.com)

Qin Liang, [19115612840@163.com](mailto:19115612840@163.com)

Xiangyin Lu, xiangyinlu0416@163.com

Deding Su, [dedingsu@163.com](mailto:dedingsu@163.com)

Xin Xu, [Xuxin13271817790@163.com](mailto:Xuxin13271817790@163.com)

Julien Pirrello, julien.pirrello@toulouse-inp.fr

Ying Gao, yinggao@cqu.edu.cn

Baowen Huang, huangbaowen2009@163.com;

Zhengguo Li, [zhengguoli@cqu.edu.cn](mailto:zhengguoli@cqu.edu.cn)


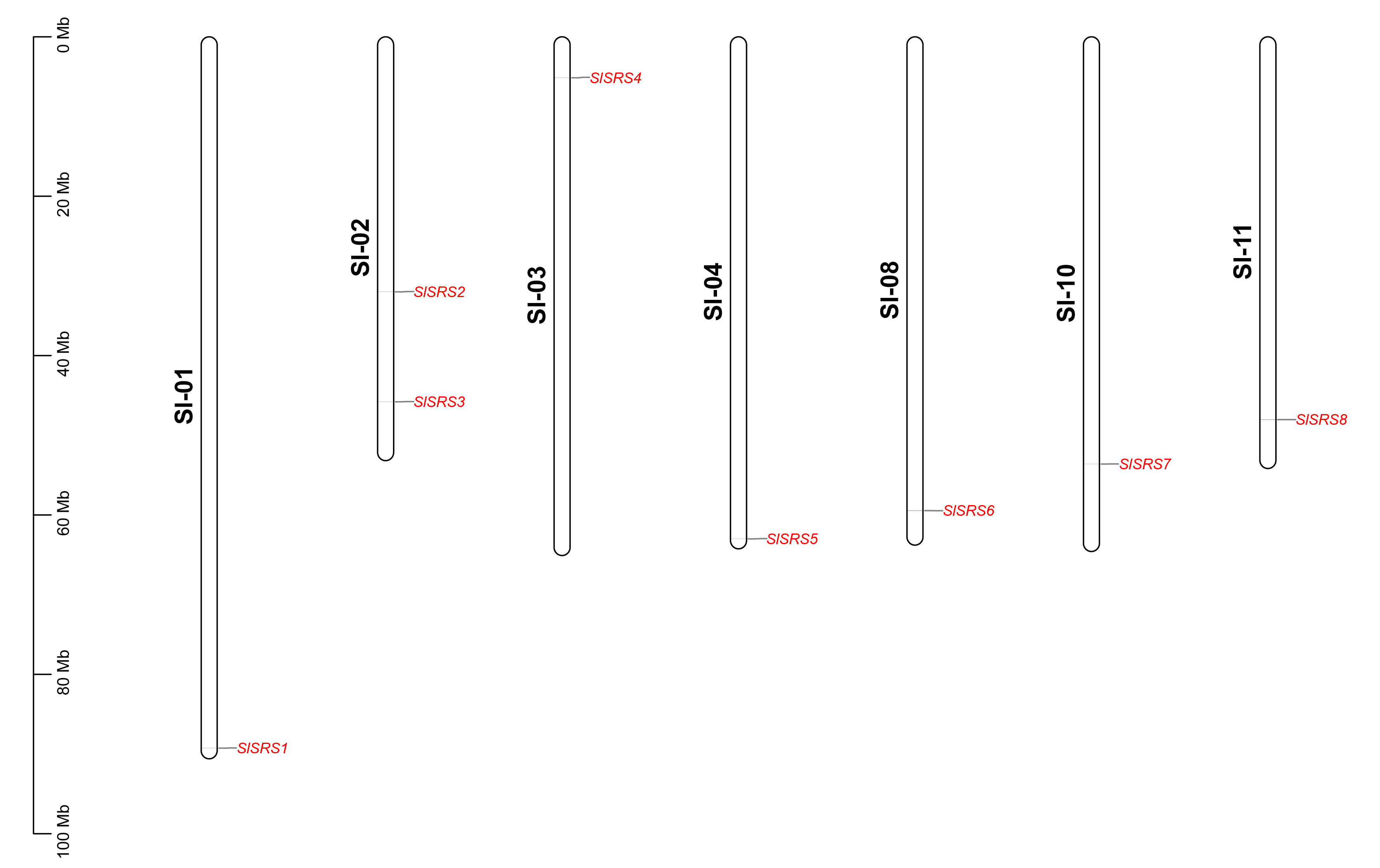
**Figure S1.** Distribution of *SRS* genes on the tomato chromosomes. The chromosome numbers are indicated at the left of each bar.

**Table S1.**  Detailed information the *cis*-elements of *SlSRS* genes

| Gene ID | *Cis*-elements | Sequences | Location | Base number | Functions |
| --- | --- | --- | --- | --- | --- |
| *Solyc01g110140* | ABRE | ACGTG | 28 | 5 | cis-acting element involved in the abscisic acid responsiveness |
| *Solyc01g110140* | ABRE | CACGTG | 1483 | 6 | cis-acting element involved in the abscisic acid responsiveness |
| *Solyc01g110140* | ABRE | ACGTG | 1484 | 5 | cis-acting element involved in the abscisic acid responsiveness |
| *Solyc01g110140* | CAT-box | GCCACT | 1071 | 6 | cis-acting regulatory element related to meristem expression |
| *Solyc01g110140* | AE-box | AGAAACTT | 1877 | 8 | part of a module for light response |
| *Solyc01g110140* | AuxRE | TGTCTCAATAAG | 646 | 11 | part of an auxin-responsive element |
| *Solyc01g110140* | TGACG-motif | TGACG | 544 | 5 | cis-acting regulatory element involved in the MeJA-responsiveness |
| *Solyc01g110140* | TGACG-motif | TGACG | 705 | 5 | cis-acting regulatory element involved in the MeJA-responsiveness |
| *Solyc01g110140* | CGTCA-motif | CGTCA | 544 | 5 | cis-acting regulatory element involved in the MeJA-responsiveness |
| *Solyc01g110140* | CGTCA-motif | CGTCA | 705 | 5 | cis-acting regulatory element involved in the MeJA-responsiveness |
| *Solyc01g110140* | TATA-box | TATACA | 8 | 6 | core promoter element around -30 of transcription start |
| *Solyc01g110140* | TATA-box | TATA | 10 | 4 | core promoter element around -30 of transcription start |
| *Solyc01g110140* | TATA-box | TATA | 32 | 4 | core promoter element around -30 of transcription start |
| *Solyc01g110140* | TATA-box | ATTATA | 65 | 6 | core promoter element around -30 of transcription start |
| *Solyc01g110140* | TATA-box | TATAA | 66 | 5 | core promoter element around -30 of transcription start |
| *Solyc01g110140* | TATA-box | TATA | 67 | 4 | core promoter element around -30 of transcription start |
| *Solyc01g110140* | TATA-box | TATACA | 72 | 6 | core promoter element around -30 of transcription start |
| *Solyc01g110140* | TATA-box | TATA | 74 | 4 | core promoter element around -30 of transcription start |
| *Solyc01g110140* | TATA-box | TATACA | 82 | 6 | core promoter element around -30 of transcription start |
| *Solyc01g110140* | TATA-box | TATA | 84 | 4 | core promoter element around -30 of transcription start |
| *Solyc01g110140* | TATA-box | TATAAAA | 110 | 7 | core promoter element around -30 of transcription start |
| *Solyc01g110140* | TATA-box | TATAAA | 111 | 6 | core promoter element around -30 of transcription start |
| *Solyc01g110140* | TATA-box | TATATAA | 112 | 7 | core promoter element around -30 of transcription start |
| *Solyc01g110140* | TATA-box | TATATA | 113 | 6 | core promoter element around -30 of transcription start |
| *Solyc01g110140* | TATA-box | ATATAA | 114 | 6 | core promoter element around -30 of transcription start |
| *Solyc01g110140* | TATA-box | TATA | 115 | 4 | core promoter element around -30 of transcription start |
| *Solyc01g110140* | TATA-box | TATATA | 131 | 6 | core promoter element around -30 of transcription start |
| *Solyc01g110140* | TATA-box | ATATAT | 132 | 6 | core promoter element around -30 of transcription start |
| *Solyc01g110140* | TATA-box | TATA | 133 | 4 | core promoter element around -30 of transcription start |
| *Solyc01g110140* | TATA-box | TATACA | 149 | 6 | core promoter element around -30 of transcription start |
| *Solyc01g110140* | TATA-box | TATATA | 151 | 6 | core promoter element around -30 of transcription start |
| *Solyc01g110140* | TATA-box | ATATAT | 152 | 6 | core promoter element around -30 of transcription start |
| *Solyc01g110140* | TATA-box | TATATA | 153 | 6 | core promoter element around -30 of transcription start |
| *Solyc01g110140* | TATA-box | ATATAT | 154 | 6 | core promoter element around -30 of transcription start |
| *Solyc01g110140* | TATA-box | TATATA | 155 | 6 | core promoter element around -30 of transcription start |
| *Solyc01g110140* | TATA-box | ATATAA | 156 | 6 | core promoter element around -30 of transcription start |
| *Solyc01g110140* | TATA-box | TATA | 157 | 4 | core promoter element around -30 of transcription start |
| *Solyc01g110140* | TATA-box | TATAAAA | 180 | 7 | core promoter element around -30 of transcription start |
| *Solyc01g110140* | TATA-box | TATAAA | 181 | 6 | core promoter element around -30 of transcription start |
| *Solyc01g110140* | TATA-box | TATAA | 182 | 5 | core promoter element around -30 of transcription start |
| *Solyc01g110140* | TATA-box | TATA | 183 | 4 | core promoter element around -30 of transcription start |
| *Solyc01g110140* | TATA-box | TATAAAA | 196 | 7 | core promoter element around -30 of transcription start |
| *Solyc01g110140* | TATA-box | TATAAA | 197 | 6 | core promoter element around -30 of transcription start |
| *Solyc01g110140* | TATA-box | TATAA | 198 | 5 | core promoter element around -30 of transcription start |
| *Solyc01g110140* | TATA-box | TATA | 199 | 4 | core promoter element around -30 of transcription start |
| *Solyc01g110140* | TATA-box | TATTTAAA | 201 | 8 | core promoter element around -30 of transcription start |
| *Solyc01g110140* | TATA-box | ATATAT | 236 | 6 | core promoter element around -30 of transcription start |
| *Solyc01g110140* | TATA-box | TATA | 237 | 4 | core promoter element around -30 of transcription start |
| *Solyc01g110140* | TATA-box | TATA | 291 | 4 | core promoter element around -30 of transcription start |
| *Solyc01g110140* | TATA-box | TATTTAAA | 421 | 8 | core promoter element around -30 of transcription start |
| *Solyc01g110140* | TATA-box | TATTTAAA | 434 | 8 | core promoter element around -30 of transcription start |
| *Solyc01g110140* | TATA-box | TATAA | 461 | 5 | core promoter element around -30 of transcription start |
| *Solyc01g110140* | TATA-box | TATA | 462 | 4 | core promoter element around -30 of transcription start |
| *Solyc01g110140* | TATA-box | TATA | 480 | 4 | core promoter element around -30 of transcription start |
| *Solyc01g110140* | TATA-box | TATAAAT | 484 | 7 | core promoter element around -30 of transcription start |
| *Solyc01g110140* | TATA-box | taTATAAAtc | 485 | 9 | core promoter element around -30 of transcription start |
| *Solyc01g110140* | TATA-box | TATAA | 486 | 5 | core promoter element around -30 of transcription start |
| *Solyc01g110140* | TATA-box | TATA | 487 | 4 | core promoter element around -30 of transcription start |
| *Solyc01g110140* | TATA-box | TATA | 618 | 4 | core promoter element around -30 of transcription start |
| *Solyc01g110140* | TATA-box | ATATAT | 757 | 6 | core promoter element around -30 of transcription start |
| *Solyc01g110140* | TATA-box | TATA | 758 | 4 | core promoter element around -30 of transcription start |
| *Solyc01g110140* | TATA-box | TATAAATA | 788 | 8 | core promoter element around -30 of transcription start |
| *Solyc01g110140* | TATA-box | TATAAAT | 789 | 7 | core promoter element around -30 of transcription start |
| *Solyc01g110140* | TATA-box | TATAAA | 790 | 6 | core promoter element around -30 of transcription start |
| *Solyc01g110140* | TATA-box | TATAA | 791 | 5 | core promoter element around -30 of transcription start |
| *Solyc01g110140* | TATA-box | TATA | 792 | 4 | core promoter element around -30 of transcription start |
| *Solyc01g110140* | TATA-box | ATTATA | 798 | 6 | core promoter element around -30 of transcription start |
| *Solyc01g110140* | TATA-box | TATAA | 799 | 5 | core promoter element around -30 of transcription start |
| *Solyc01g110140* | TATA-box | TATA | 800 | 4 | core promoter element around -30 of transcription start |
| *Solyc01g110140* | TATA-box | TATA | 985 | 4 | core promoter element around -30 of transcription start |
| *Solyc01g110140* | TATA-box | ATTATA | 1028 | 6 | core promoter element around -30 of transcription start |
| *Solyc01g110140* | TATA-box | TATAA | 1029 | 5 | core promoter element around -30 of transcription start |
| *Solyc01g110140* | TATA-box | TATA | 1030 | 4 | core promoter element around -30 of transcription start |
| *Solyc01g110140* | TATA-box | taTATAAAtc | 1086 | 9 | core promoter element around -30 of transcription start |
| *Solyc01g110140* | TATA-box | TATAAAT | 1087 | 7 | core promoter element around -30 of transcription start |
| *Solyc01g110140* | TATA-box | TATAAA | 1088 | 6 | core promoter element around -30 of transcription start |
| *Solyc01g110140* | TATA-box | TATAA | 1089 | 5 | core promoter element around -30 of transcription start |
| *Solyc01g110140* | TATA-box | TATA | 1090 | 4 | core promoter element around -30 of transcription start |
| *Solyc01g110140* | TATA-box | ATTATA | 1095 | 6 | core promoter element around -30 of transcription start |
| *Solyc01g110140* | TATA-box | TATAA | 1096 | 5 | core promoter element around -30 of transcription start |
| *Solyc01g110140* | TATA-box | TATA | 1097 | 4 | core promoter element around -30 of transcription start |
| *Solyc01g110140* | TATA-box | ATTATA | 1113 | 6 | core promoter element around -30 of transcription start |
| *Solyc01g110140* | TATA-box | TATAA | 1114 | 5 | core promoter element around -30 of transcription start |
| *Solyc01g110140* | TATA-box | TATA | 1115 | 4 | core promoter element around -30 of transcription start |
| *Solyc01g110140* | TATA-box | TATTTAAA | 1151 | 8 | core promoter element around -30 of transcription start |
| *Solyc01g110140* | TATA-box | TATAAAT | 1269 | 7 | core promoter element around -30 of transcription start |
| *Solyc01g110140* | TATA-box | TATAAA | 1270 | 6 | core promoter element around -30 of transcription start |
| *Solyc01g110140* | TATA-box | TATAA | 1271 | 5 | core promoter element around -30 of transcription start |
| *Solyc01g110140* | TATA-box | TATA | 1272 | 4 | core promoter element around -30 of transcription start |
| *Solyc01g110140* | TATA-box | TATA | 1411 | 4 | core promoter element around -30 of transcription start |
| *Solyc01g110140* | TATA-box | TATAAAA | 1578 | 7 | core promoter element around -30 of transcription start |
| *Solyc01g110140* | TATA-box | TATAAA | 1579 | 6 | core promoter element around -30 of transcription start |
| *Solyc01g110140* | TATA-box | TATAA | 1580 | 5 | core promoter element around -30 of transcription start |
| *Solyc01g110140* | TATA-box | TATA | 1581 | 4 | core promoter element around -30 of transcription start |
| *Solyc01g110140* | TATA-box | TATAAATA | 1633 | 8 | core promoter element around -30 of transcription start |
| *Solyc01g110140* | TATA-box | TATAAAT | 1634 | 7 | core promoter element around -30 of transcription start |
| *Solyc01g110140* | TATA-box | TATAAA | 1635 | 6 | core promoter element around -30 of transcription start |
| *Solyc01g110140* | TATA-box | TATAA | 1636 | 5 | core promoter element around -30 of transcription start |
| *Solyc01g110140* | TATA-box | TATA | 1637 | 4 | core promoter element around -30 of transcription start |
| *Solyc01g110140* | TATA-box | ATTATA | 1652 | 6 | core promoter element around -30 of transcription start |
| *Solyc01g110140* | TATA-box | TATAA | 1653 | 5 | core promoter element around -30 of transcription start |
| *Solyc01g110140* | TATA-box | TATA | 1654 | 4 | core promoter element around -30 of transcription start |
| *Solyc01g110140* | TATA-box | ATTATA | 1703 | 6 | core promoter element around -30 of transcription start |
| *Solyc01g110140* | TATA-box | TATAA | 1704 | 5 | core promoter element around -30 of transcription start |
| *Solyc01g110140* | TATA-box | TATA | 1705 | 4 | core promoter element around -30 of transcription start |
| *Solyc01g110140* | TATA-box | TATA | 1805 | 4 | core promoter element around -30 of transcription start |
| *Solyc01g110140* | TATA-box | ATTATA | 1975 | 6 | core promoter element around -30 of transcription start |
| *Solyc01g110140* | TATA-box | TATAA | 1976 | 5 | core promoter element around -30 of transcription start |
| *Solyc01g110140* | TATA-box | TATA | 1977 | 4 | core promoter element around -30 of transcription start |
| *Solyc01g110140* | MBS | CAACTG | 1421 | 6 | MYB binding site involved in drought-inducibility |
| *Solyc01g110140* | MBS | CAACTG | 1937 | 6 | MYB binding site involved in drought-inducibility |
| *Solyc01g110140* | P-box | CCTTTTG | 373 | 7 | gibberellin-responsive element |
| *Solyc01g110140* | ARE | AAACCA | 1615 | 6 | cis-acting regulatory element essential for the anaerobic induction |
| *Solyc01g110140* | ARE | AAACCA | 1920 | 6 | cis-acting regulatory element essential for the anaerobic induction |
| *Solyc01g110140* | MRE | AACCTAA | 921 | 7 | MYB binding site involved in light responsiveness |
| *Solyc01g110140* | Box 4 | ATTAAT | 533 | 6 | part of a conserved DNA module involved in light responsiveness |
| *Solyc01g110140* | Box 4 | ATTAAT | 553 | 6 | part of a conserved DNA module involved in light responsiveness |
| *Solyc01g110140* | Box 4 | ATTAAT | 821 | 6 | part of a conserved DNA module involved in light responsiveness |
| *Solyc01g110140* | Box 4 | ATTAAT | 1042 | 6 | part of a conserved DNA module involved in light responsiveness |
| *Solyc01g110140* | Box 4 | ATTAAT | 1100 | 6 | part of a conserved DNA module involved in light responsiveness |
| *Solyc01g110140* | Box 4 | ATTAAT | 1365 | 6 | part of a conserved DNA module involved in light responsiveness |
| *Solyc01g110140* | Box 4 | ATTAAT | 1507 | 6 | part of a conserved DNA module involved in light responsiveness |
| *Solyc01g110140* | Box 4 | ATTAAT | 1737 | 6 | part of a conserved DNA module involved in light responsiveness |
| *Solyc01g110140* | Box 4 | ATTAAT | 1987 | 6 | part of a conserved DNA module involved in light responsiveness |
| *Solyc01g110140* | ACE | CTAACGTATT | 273 | 9 | cis-acting element involved in light responsiveness |
| *Solyc01g110140* | G-Box | CACGTG | 1483 | 6 | cis-acting regulatory element involved in light responsiveness |
| *Solyc01g110140* | CAAT-box | CAAAT | 49 | 5 | common cis-acting element in promoter and enhancer regions |
| *Solyc01g110140* | CAAT-box | CAAAT | 161 | 5 | common cis-acting element in promoter and enhancer regions |
| *Solyc01g110140* | CAAT-box | CCAAT | 314 | 5 | common cis-acting element in promoter and enhancer regions |
| *Solyc01g110140* | CAAT-box | CCAAT | 345 | 5 | common cis-acting element in promoter and enhancer regions |
| *Solyc01g110140* | CAAT-box | CAAAT | 530 | 5 | common cis-acting element in promoter and enhancer regions |
| *Solyc01g110140* | CAAT-box | CCAAT | 580 | 5 | common cis-acting element in promoter and enhancer regions |
| *Solyc01g110140* | CAAT-box | CAAAT | 600 | 5 | common cis-acting element in promoter and enhancer regions |
| *Solyc01g110140* | CAAT-box | CAAAT | 777 | 5 | common cis-acting element in promoter and enhancer regions |
| *Solyc01g110140* | CAAT-box | CCAAT | 933 | 5 | common cis-acting element in promoter and enhancer regions |
| *Solyc01g110140* | CAAT-box | CCAAT | 1034 | 5 | common cis-acting element in promoter and enhancer regions |
| *Solyc01g110140* | CAAT-box | CCAAT | 1046 | 5 | common cis-acting element in promoter and enhancer regions |
| *Solyc01g110140* | CAAT-box | CCAAT | 1120 | 5 | common cis-acting element in promoter and enhancer regions |
| *Solyc01g110140* | CAAT-box | CAAAT | 1203 | 5 | common cis-acting element in promoter and enhancer regions |
| *Solyc01g110140* | CAAT-box | CCAAT | 1249 | 5 | common cis-acting element in promoter and enhancer regions |
| *Solyc01g110140* | CAAT-box | CAAAT | 1344 | 5 | common cis-acting element in promoter and enhancer regions |
| *Solyc01g110140* | CAAT-box | CAAAT | 1350 | 5 | common cis-acting element in promoter and enhancer regions |
| *Solyc01g110140* | CAAT-box | CAAAT | 1459 | 5 | common cis-acting element in promoter and enhancer regions |
| *Solyc01g110140* | CAAT-box | CAAAT | 1629 | 5 | common cis-acting element in promoter and enhancer regions |
| *Solyc01g110140* | CAAT-box | CCAAT | 1748 | 5 | common cis-acting element in promoter and enhancer regions |
| *Solyc01g110140* | CAAT-box | CAAAT | 1854 | 5 | common cis-acting element in promoter and enhancer regions |
| *Solyc01g110140* | CAAT-box | CAAAT | 1862 | 5 | common cis-acting element in promoter and enhancer regions |
| *Solyc01g110140* | CAAT-box | CAAAT | 1924 | 5 | common cis-acting element in promoter and enhancer regions |
| *Solyc01g110140* | G-box | TACGTG | 28 | 6 | cis-acting regulatory element involved in light responsiveness |
| *Solyc01g110140* | G-box | CACGAC | 442 | 6 | cis-acting regulatory element involved in light responsiveness |
| *Solyc01g110140* | G-box | CACGTG | 1483 | 6 | cis-acting regulatory element involved in light responsiveness |
| *Solyc02g062400* | TCA-element | TCAGAAGAGG | 261 | 9 | cis-acting element involved in salicylic acid responsiveness |
| *Solyc02g062400* | TCA-element | CCATCTTTTT | 718 | 9 | cis-acting element involved in salicylic acid responsiveness |
| *Solyc02g062400* | HD-Zip 1 | CAAT(A/T)ATTG | 957 | 8.5 | element involved in differentiation of the palisade mesophyll cells |
| *Solyc02g062400* | HD-Zip 1 | CAAT(A/T)ATTG | 1351 | 8 | element involved in differentiation of the palisade mesophyll cells |
| *Solyc02g062400* | circadian | CAAAGATATC | 12 | 9 | cis-acting regulatory element involved in circadian control |
| *Solyc02g062400* | circadian | CAAAGATATC | 988 | 9 | cis-acting regulatory element involved in circadian control |
| *Solyc02g062400* | GATA-motif | AAGATAAGATT | 453 | 10 | part of a light responsive element |
| *Solyc02g062400* | GATA-motif | AAGATAAGATT | 553 | 10 | part of a light responsive element |
| *Solyc02g062400* | GA-motif | ATAGATAA | 141 | 8 | part of a light responsive element |
| *Solyc02g062400* | CAAT-box | CAAAT | 244 | 5 | common cis-acting element in promoter and enhancer regions |
| *Solyc02g062400* | CAAT-box | CCAAT | 247 | 5 | common cis-acting element in promoter and enhancer regions |
| *Solyc02g062400* | CAAT-box | CAAAT | 334 | 5 | common cis-acting element in promoter and enhancer regions |
| *Solyc02g062400* | CAAT-box | CAAAT | 426 | 5 | common cis-acting element in promoter and enhancer regions |
| *Solyc02g062400* | CAAT-box | CAAAT | 526 | 5 | common cis-acting element in promoter and enhancer regions |
| *Solyc02g062400* | CAAT-box | CAAAT | 867 | 5 | common cis-acting element in promoter and enhancer regions |
| *Solyc02g062400* | CAAT-box | CAAAT | 1001 | 5 | common cis-acting element in promoter and enhancer regions |
| *Solyc02g062400* | CAAT-box | CCAAT | 1324 | 5 | common cis-acting element in promoter and enhancer regions |
| *Solyc02g062400* | CAAT-box | CAAAT | 1427 | 5 | common cis-acting element in promoter and enhancer regions |
| *Solyc02g062400* | CAAT-box | CAAAT | 1524 | 5 | common cis-acting element in promoter and enhancer regions |
| *Solyc02g062400* | CAAT-box | CAAAT | 1923 | 5 | common cis-acting element in promoter and enhancer regions |
| *Solyc02g062400* | Box 4 | ATTAAT | 127 | 6 | part of a conserved DNA module involved in light responsiveness |
| *Solyc02g062400* | Box 4 | ATTAAT | 162 | 6 | part of a conserved DNA module involved in light responsiveness |
| *Solyc02g062400* | Box 4 | ATTAAT | 923 | 6 | part of a conserved DNA module involved in light responsiveness |
| *Solyc02g062400* | Box 4 | ATTAAT | 1063 | 6 | part of a conserved DNA module involved in light responsiveness |
| *Solyc02g062400* | Box 4 | ATTAAT | 1704 | 6 | part of a conserved DNA module involved in light responsiveness |
| *Solyc02g062400* | Box 4 | ATTAAT | 1965 | 6 | part of a conserved DNA module involved in light responsiveness |
| *Solyc02g062400* | ACE | GCGACGTACC | 1315 | 9 | cis-acting element involved in light responsiveness |
| *Solyc02g062400* | TATA-box | TATACA | 47 | 6 | core promoter element around -30 of transcription start |
| *Solyc02g062400* | TATA-box | TATA | 49 | 4 | core promoter element around -30 of transcription start |
| *Solyc02g062400* | TATA-box | TATAAA | 80 | 6 | core promoter element around -30 of transcription start |
| *Solyc02g062400* | TATA-box | TATATAA | 81 | 7 | core promoter element around -30 of transcription start |
| *Solyc02g062400* | TATA-box | TATATA | 82 | 6 | core promoter element around -30 of transcription start |
| *Solyc02g062400* | TATA-box | ATATAT | 83 | 6 | core promoter element around -30 of transcription start |
| *Solyc02g062400* | TATA-box | TATATA | 84 | 6 | core promoter element around -30 of transcription start |
| *Solyc02g062400* | TATA-box | TATA | 86 | 4 | core promoter element around -30 of transcription start |
| *Solyc02g062400* | TATA-box | TATTTAAA | 132 | 8 | core promoter element around -30 of transcription start |
| *Solyc02g062400* | TATA-box | TATA | 153 | 4 | core promoter element around -30 of transcription start |
| *Solyc02g062400* | TATA-box | ATATAA | 212 | 6 | core promoter element around -30 of transcription start |
| *Solyc02g062400* | TATA-box | TATA | 213 | 4 | core promoter element around -30 of transcription start |
| *Solyc02g062400* | TATA-box | TATA | 256 | 4 | core promoter element around -30 of transcription start |
| *Solyc02g062400* | TATA-box | TATAAAA | 369 | 7 | core promoter element around -30 of transcription start |
| *Solyc02g062400* | TATA-box | TATAAA | 370 | 6 | core promoter element around -30 of transcription start |
| *Solyc02g062400* | TATA-box | TATAA | 371 | 5 | core promoter element around -30 of transcription start |
| *Solyc02g062400* | TATA-box | TATATTTATATTT | 372 | 12 | core promoter element around -30 of transcription start |
| *Solyc02g062400* | TATA-box | TATAAATA | 374 | 8 | core promoter element around -30 of transcription start |
| *Solyc02g062400* | TATA-box | TATAAAT | 375 | 7 | core promoter element around -30 of transcription start |
| *Solyc02g062400* | TATA-box | TATAAA | 376 | 6 | core promoter element around -30 of transcription start |
| *Solyc02g062400* | TATA-box | TATAA | 377 | 5 | core promoter element around -30 of transcription start |
| *Solyc02g062400* | TATA-box | TATA | 378 | 4 | core promoter element around -30 of transcription start |
| *Solyc02g062400* | TATA-box | TATATA | 401 | 6 | core promoter element around -30 of transcription start |
| *Solyc02g062400* | TATA-box | ATATAT | 402 | 6 | core promoter element around -30 of transcription start |
| *Solyc02g062400* | TATA-box | TATA | 403 | 4 | core promoter element around -30 of transcription start |
| *Solyc02g062400* | TATA-box | ATTATA | 411 | 6 | core promoter element around -30 of transcription start |
| *Solyc02g062400* | TATA-box | TATAA | 412 | 5 | core promoter element around -30 of transcription start |
| *Solyc02g062400* | TATA-box | TATA | 413 | 4 | core promoter element around -30 of transcription start |
| *Solyc02g062400* | TATA-box | TATAAAA | 474 | 7 | core promoter element around -30 of transcription start |
| *Solyc02g062400* | TATA-box | TATAAA | 475 | 6 | core promoter element around -30 of transcription start |
| *Solyc02g062400* | TATA-box | TATATAA | 476 | 7 | core promoter element around -30 of transcription start |
| *Solyc02g062400* | TATA-box | TATATA | 477 | 6 | core promoter element around -30 of transcription start |
| *Solyc02g062400* | TATA-box | ATATAT | 478 | 6 | core promoter element around -30 of transcription start |
| *Solyc02g062400* | TATA-box | TATATA | 479 | 6 | core promoter element around -30 of transcription start |
| *Solyc02g062400* | TATA-box | ATATAT | 480 | 6 | core promoter element around -30 of transcription start |
| *Solyc02g062400* | TATA-box | TATATA | 481 | 6 | core promoter element around -30 of transcription start |
| *Solyc02g062400* | TATA-box | ATATAT | 482 | 6 | core promoter element around -30 of transcription start |
| *Solyc02g062400* | TATA-box | TATA | 483 | 4 | core promoter element around -30 of transcription start |
| *Solyc02g062400* | TATA-box | TATATA | 501 | 6 | core promoter element around -30 of transcription start |
| *Solyc02g062400* | TATA-box | ATATAT | 502 | 6 | core promoter element around -30 of transcription start |
| *Solyc02g062400* | TATA-box | TATA | 503 | 4 | core promoter element around -30 of transcription start |
| *Solyc02g062400* | TATA-box | ATTATA | 511 | 6 | core promoter element around -30 of transcription start |
| *Solyc02g062400* | TATA-box | TATAA | 512 | 5 | core promoter element around -30 of transcription start |
| *Solyc02g062400* | TATA-box | TATA | 513 | 4 | core promoter element around -30 of transcription start |
| *Solyc02g062400* | TATA-box | TATAAAA | 574 | 7 | core promoter element around -30 of transcription start |
| *Solyc02g062400* | TATA-box | TATAAA | 575 | 6 | core promoter element around -30 of transcription start |
| *Solyc02g062400* | TATA-box | TATATAA | 576 | 7 | core promoter element around -30 of transcription start |
| *Solyc02g062400* | TATA-box | TATATA | 577 | 6 | core promoter element around -30 of transcription start |
| *Solyc02g062400* | TATA-box | ATATAT | 578 | 6 | core promoter element around -30 of transcription start |
| *Solyc02g062400* | TATA-box | TATATA | 579 | 6 | core promoter element around -30 of transcription start |
| *Solyc02g062400* | TATA-box | ATATAT | 580 | 6 | core promoter element around -30 of transcription start |
| *Solyc02g062400* | TATA-box | TATATA | 581 | 6 | core promoter element around -30 of transcription start |
| *Solyc02g062400* | TATA-box | ATATAT | 582 | 6 | core promoter element around -30 of transcription start |
| *Solyc02g062400* | TATA-box | TATATA | 583 | 6 | core promoter element around -30 of transcription start |
| *Solyc02g062400* | TATA-box | ATATAT | 584 | 6 | core promoter element around -30 of transcription start |
| *Solyc02g062400* | TATA-box | TATATA | 585 | 6 | core promoter element around -30 of transcription start |
| *Solyc02g062400* | TATA-box | ATATAT | 586 | 6 | core promoter element around -30 of transcription start |
| *Solyc02g062400* | TATA-box | TATATA | 587 | 6 | core promoter element around -30 of transcription start |
| *Solyc02g062400* | TATA-box | ATATAT | 588 | 6 | core promoter element around -30 of transcription start |
| *Solyc02g062400* | TATA-box | TATATA | 589 | 6 | core promoter element around -30 of transcription start |
| *Solyc02g062400* | TATA-box | ATATAT | 590 | 6 | core promoter element around -30 of transcription start |
| *Solyc02g062400* | TATA-box | TATATA | 591 | 6 | core promoter element around -30 of transcription start |
| *Solyc02g062400* | TATA-box | ATATAT | 592 | 6 | core promoter element around -30 of transcription start |
| *Solyc02g062400* | TATA-box | TATATA | 593 | 6 | core promoter element around -30 of transcription start |
| *Solyc02g062400* | TATA-box | ATATAA | 594 | 6 | core promoter element around -30 of transcription start |
| *Solyc02g062400* | TATA-box | TATA | 595 | 4 | core promoter element around -30 of transcription start |
| *Solyc02g062400* | TATA-box | ATATAT | 608 | 6 | core promoter element around -30 of transcription start |
| *Solyc02g062400* | TATA-box | TATA | 609 | 4 | core promoter element around -30 of transcription start |
| *Solyc02g062400* | TATA-box | TATAAAA | 632 | 7 | core promoter element around -30 of transcription start |
| *Solyc02g062400* | TATA-box | TATAAA | 633 | 6 | core promoter element around -30 of transcription start |
| *Solyc02g062400* | TATA-box | TATAA | 634 | 5 | core promoter element around -30 of transcription start |
| *Solyc02g062400* | TATA-box | TATA | 635 | 4 | core promoter element around -30 of transcription start |
| *Solyc02g062400* | TATA-box | TACAAAA | 678 | 7 | core promoter element around -30 of transcription start |
| *Solyc02g062400* | TATA-box | ATATAT | 747 | 6 | core promoter element around -30 of transcription start |
| *Solyc02g062400* | TATA-box | TATA | 748 | 4 | core promoter element around -30 of transcription start |
| *Solyc02g062400* | TATA-box | TATA | 769 | 4 | core promoter element around -30 of transcription start |
| *Solyc02g062400* | TATA-box | taTATAAAtc | 914 | 9 | core promoter element around -30 of transcription start |
| *Solyc02g062400* | TATA-box | TATAAAT | 915 | 7 | core promoter element around -30 of transcription start |
| *Solyc02g062400* | TATA-box | TATAAA | 916 | 6 | core promoter element around -30 of transcription start |
| *Solyc02g062400* | TATA-box | TATATAA | 917 | 7 | core promoter element around -30 of transcription start |
| *Solyc02g062400* | TATA-box | TATATA | 918 | 6 | core promoter element around -30 of transcription start |
| *Solyc02g062400* | TATA-box | ATATAT | 919 | 6 | core promoter element around -30 of transcription start |
| *Solyc02g062400* | TATA-box | TATA | 920 | 4 | core promoter element around -30 of transcription start |
| *Solyc02g062400* | TATA-box | TATA | 948 | 4 | core promoter element around -30 of transcription start |
| *Solyc02g062400* | TATA-box | TATTTAAA | 977 | 8 | core promoter element around -30 of transcription start |
| *Solyc02g062400* | TATA-box | TATA | 994 | 4 | core promoter element around -30 of transcription start |
| *Solyc02g062400* | TATA-box | ATATAT | 1070 | 6 | core promoter element around -30 of transcription start |
| *Solyc02g062400* | TATA-box | TATA | 1071 | 4 | core promoter element around -30 of transcription start |
| *Solyc02g062400* | TATA-box | ATTATA | 1074 | 6 | core promoter element around -30 of transcription start |
| *Solyc02g062400* | TATA-box | TATAA | 1075 | 5 | core promoter element around -30 of transcription start |
| *Solyc02g062400* | TATA-box | TATA | 1076 | 4 | core promoter element around -30 of transcription start |
| *Solyc02g062400* | TATA-box | ATTATA | 1168 | 6 | core promoter element around -30 of transcription start |
| *Solyc02g062400* | TATA-box | TATAA | 1169 | 5 | core promoter element around -30 of transcription start |
| *Solyc02g062400* | TATA-box | TATA | 1170 | 4 | core promoter element around -30 of transcription start |
| *Solyc02g062400* | TATA-box | TATA | 1308 | 4 | core promoter element around -30 of transcription start |
| *Solyc02g062400* | TATA-box | ATTATA | 1331 | 6 | core promoter element around -30 of transcription start |
| *Solyc02g062400* | TATA-box | TATAA | 1332 | 5 | core promoter element around -30 of transcription start |
| *Solyc02g062400* | TATA-box | TATA | 1333 | 4 | core promoter element around -30 of transcription start |
| *Solyc02g062400* | TATA-box | TATA | 1420 | 4 | core promoter element around -30 of transcription start |
| *Solyc02g062400* | TATA-box | TATA | 1445 | 4 | core promoter element around -30 of transcription start |
| *Solyc02g062400* | TATA-box | ATATAT | 1466 | 6 | core promoter element around -30 of transcription start |
| *Solyc02g062400* | TATA-box | TATA | 1467 | 4 | core promoter element around -30 of transcription start |
| *Solyc02g062400* | TATA-box | TATTTAAA | 1469 | 8 | core promoter element around -30 of transcription start |
| *Solyc02g062400* | TATA-box | ATTATA | 1480 | 6 | core promoter element around -30 of transcription start |
| *Solyc02g062400* | TATA-box | TATAA | 1481 | 5 | core promoter element around -30 of transcription start |
| *Solyc02g062400* | TATA-box | TATA | 1482 | 4 | core promoter element around -30 of transcription start |
| *Solyc02g062400* | TATA-box | TATAAAA | 1555 | 7 | core promoter element around -30 of transcription start |
| *Solyc02g062400* | TATA-box | TATAAA | 1556 | 6 | core promoter element around -30 of transcription start |
| *Solyc02g062400* | TATA-box | TATAA | 1557 | 5 | core promoter element around -30 of transcription start |
| *Solyc02g062400* | TATA-box | TATA | 1558 | 4 | core promoter element around -30 of transcription start |
| *Solyc02g062400* | TATA-box | ATATAT | 1682 | 6 | core promoter element around -30 of transcription start |
| *Solyc02g062400* | TATA-box | TATATA | 1683 | 6 | core promoter element around -30 of transcription start |
| *Solyc02g062400* | TATA-box | ATATAT | 1684 | 6 | core promoter element around -30 of transcription start |
| *Solyc02g062400* | TATA-box | TATATA | 1685 | 6 | core promoter element around -30 of transcription start |
| *Solyc02g062400* | TATA-box | ATATAA | 1686 | 6 | core promoter element around -30 of transcription start |
| *Solyc02g062400* | TATA-box | TATA | 1687 | 4 | core promoter element around -30 of transcription start |
| *Solyc02g062400* | TATA-box | TATA | 1692 | 4 | core promoter element around -30 of transcription start |
| *Solyc02g062400* | TATA-box | TATAAA | 1744 | 6 | core promoter element around -30 of transcription start |
| *Solyc02g062400* | TATA-box | TATAA | 1745 | 5 | core promoter element around -30 of transcription start |
| *Solyc02g062400* | TATA-box | TATA | 1746 | 4 | core promoter element around -30 of transcription start |
| *Solyc02g062400* | TATA-box | ATTATA | 1780 | 6 | core promoter element around -30 of transcription start |
| *Solyc02g062400* | TATA-box | TATAA | 1781 | 5 | core promoter element around -30 of transcription start |
| *Solyc02g062400* | TATA-box | TATA | 1782 | 4 | core promoter element around -30 of transcription start |
| *Solyc02g062400* | TATA-box | taTATAAAg | 1800 | 9 | core promoter element around -30 of transcription start |
| *Solyc02g062400* | TATA-box | TATAAA | 1801 | 6 | core promoter element around -30 of transcription start |
| *Solyc02g062400* | TATA-box | TATATAA | 1802 | 7 | core promoter element around -30 of transcription start |
| *Solyc02g062400* | TATA-box | TATATA | 1803 | 6 | core promoter element around -30 of transcription start |
| *Solyc02g062400* | TATA-box | ATATAT | 1804 | 6 | core promoter element around -30 of transcription start |
| *Solyc02g062400* | TATA-box | TATATA | 1805 | 6 | core promoter element around -30 of transcription start |
| *Solyc02g062400* | TATA-box | TATA | 1807 | 4 | core promoter element around -30 of transcription start |
| *Solyc02g062400* | TATA-box | TATA | 1819 | 4 | core promoter element around -30 of transcription start |
| *Solyc02g062400* | TATA-box | TACAAAA | 1843 | 7 | core promoter element around -30 of transcription start |
| *Solyc02g062400* | TATA-box | TATAA | 1930 | 5 | core promoter element around -30 of transcription start |
| *Solyc02g062400* | TATA-box | TATA | 1931 | 4 | core promoter element around -30 of transcription start |
| *Solyc02g084680* | CAAT-box | CAAAT | 75 | 5 | common cis-acting element in promoter and enhancer regions |
| *Solyc02g084680* | CAAT-box | CAAAT | 657 | 5 | common cis-acting element in promoter and enhancer regions |
| *Solyc02g084680* | CAAT-box | CAAAT | 703 | 5 | common cis-acting element in promoter and enhancer regions |
| *Solyc02g084680* | CAAT-box | CAAAT | 998 | 5 | common cis-acting element in promoter and enhancer regions |
| *Solyc02g084680* | CAAT-box | CAAAT | 1100 | 5 | common cis-acting element in promoter and enhancer regions |
| *Solyc02g084680* | CAAT-box | CCAAT | 1320 | 5 | common cis-acting element in promoter and enhancer regions |
| *Solyc02g084680* | CAAT-box | CAAAT | 1365 | 5 | common cis-acting element in promoter and enhancer regions |
| *Solyc02g084680* | CAAT-box | TGCAAATCT | 1523 | 9 | common cis-acting element in promoter and enhancer regions |
| *Solyc02g084680* | CAAT-box | CAAAT | 1525 | 5 | common cis-acting element in promoter and enhancer regions |
| *Solyc02g084680* | CAAT-box | CAAAT | 1659 | 5 | common cis-acting element in promoter and enhancer regions |
| *Solyc02g084680* | CAAT-box | CAAAT | 1780 | 5 | common cis-acting element in promoter and enhancer regions |
| *Solyc02g084680* | CAAT-box | CAAAT | 1864 | 5 | common cis-acting element in promoter and enhancer regions |
| *Solyc02g084680* | G-Box | CACGTG | 1295 | 6 | cis-acting regulatory element involved in light responsiveness |
| *Solyc02g084680* | G-box | tgACACGTGGCA | 1292 | 11 | cis-acting regulatory element involved in light responsiveness |
| *Solyc02g084680* | G-box | ACACGTGT | 1294 | 8 | cis-acting regulatory element involved in light responsiveness |
| *Solyc02g084680* | G-box | CACGTG | 1295 | 6 | cis-acting regulatory element involved in light responsiveness |
| *Solyc02g084680* | Box 4 | ATTAAT | 261 | 6 | part of a conserved DNA module involved in light responsiveness |
| *Solyc02g084680* | Box 4 | ATTAAT | 574 | 6 | part of a conserved DNA module involved in light responsiveness |
| *Solyc02g084680* | Box 4 | ATTAAT | 612 | 6 | part of a conserved DNA module involved in light responsiveness |
| *Solyc02g084680* | GA-motif | ATAGATAA | 27 | 8 | part of a light responsive element |
| *Solyc02g084680* | GA-motif | ATAGATAA | 848 | 8 | part of a light responsive element |
| *Solyc02g084680* | TATA-box | ATTATA | 12 | 6 | core promoter element around -30 of transcription start |
| *Solyc02g084680* | TATA-box | TATAA | 13 | 5 | core promoter element around -30 of transcription start |
| *Solyc02g084680* | TATA-box | TATA | 14 | 4 | core promoter element around -30 of transcription start |
| *Solyc02g084680* | TATA-box | TATAAAT | 34 | 7 | core promoter element around -30 of transcription start |
| *Solyc02g084680* | TATA-box | TATAAA | 35 | 6 | core promoter element around -30 of transcription start |
| *Solyc02g084680* | TATA-box | TATAA | 36 | 5 | core promoter element around -30 of transcription start |
| *Solyc02g084680* | TATA-box | TATA | 37 | 4 | core promoter element around -30 of transcription start |
| *Solyc02g084680* | TATA-box | ATATAA | 107 | 6 | core promoter element around -30 of transcription start |
| *Solyc02g084680* | TATA-box | TATA | 108 | 4 | core promoter element around -30 of transcription start |
| *Solyc02g084680* | TATA-box | TATAA | 155 | 5 | core promoter element around -30 of transcription start |
| *Solyc02g084680* | TATA-box | TATA | 156 | 4 | core promoter element around -30 of transcription start |
| *Solyc02g084680* | TATA-box | TATAAAA | 225 | 7 | core promoter element around -30 of transcription start |
| *Solyc02g084680* | TATA-box | TATAAA | 226 | 6 | core promoter element around -30 of transcription start |
| *Solyc02g084680* | TATA-box | TATATAA | 227 | 7 | core promoter element around -30 of transcription start |
| *Solyc02g084680* | TATA-box | TATATA | 228 | 6 | core promoter element around -30 of transcription start |
| *Solyc02g084680* | TATA-box | ATATAT | 229 | 6 | core promoter element around -30 of transcription start |
| *Solyc02g084680* | TATA-box | TATA | 230 | 4 | core promoter element around -30 of transcription start |
| *Solyc02g084680* | TATA-box | TATAAAA | 242 | 7 | core promoter element around -30 of transcription start |
| *Solyc02g084680* | TATA-box | TATAAA | 243 | 6 | core promoter element around -30 of transcription start |
| *Solyc02g084680* | TATA-box | TATAA | 244 | 5 | core promoter element around -30 of transcription start |
| *Solyc02g084680* | TATA-box | TATA | 245 | 4 | core promoter element around -30 of transcription start |
| *Solyc02g084680* | TATA-box | TACAAAA | 280 | 7 | core promoter element around -30 of transcription start |
| *Solyc02g084680* | TATA-box | TATAAAT | 330 | 7 | core promoter element around -30 of transcription start |
| *Solyc02g084680* | TATA-box | TATAAA | 331 | 6 | core promoter element around -30 of transcription start |
| *Solyc02g084680* | TATA-box | TATAA | 332 | 5 | core promoter element around -30 of transcription start |
| *Solyc02g084680* | TATA-box | TATA | 333 | 4 | core promoter element around -30 of transcription start |
| *Solyc02g084680* | TATA-box | TACAAAA | 339 | 7 | core promoter element around -30 of transcription start |
| *Solyc02g084680* | TATA-box | ATATAA | 367 | 6 | core promoter element around -30 of transcription start |
| *Solyc02g084680* | TATA-box | TATA | 368 | 4 | core promoter element around -30 of transcription start |
| *Solyc02g084680* | TATA-box | ATATAT | 452 | 6 | core promoter element around -30 of transcription start |
| *Solyc02g084680* | TATA-box | TATA | 453 | 4 | core promoter element around -30 of transcription start |
| *Solyc02g084680* | TATA-box | ATATAT | 537 | 6 | core promoter element around -30 of transcription start |
| *Solyc02g084680* | TATA-box | TATA | 538 | 4 | core promoter element around -30 of transcription start |
| *Solyc02g084680* | TATA-box | ATATAT | 587 | 6 | core promoter element around -30 of transcription start |
| *Solyc02g084680* | TATA-box | TATA | 588 | 4 | core promoter element around -30 of transcription start |
| *Solyc02g084680* | TATA-box | TATAA | 668 | 5 | core promoter element around -30 of transcription start |
| *Solyc02g084680* | TATA-box | TATA | 669 | 4 | core promoter element around -30 of transcription start |
| *Solyc02g084680* | TATA-box | TATAAA | 737 | 6 | core promoter element around -30 of transcription start |
| *Solyc02g084680* | TATA-box | TATAA | 738 | 5 | core promoter element around -30 of transcription start |
| *Solyc02g084680* | TATA-box | TATA | 739 | 4 | core promoter element around -30 of transcription start |
| *Solyc02g084680* | TATA-box | TAAAGATT | 741 | 8 | core promoter element around -30 of transcription start |
| *Solyc02g084680* | TATA-box | TATATA | 760 | 6 | core promoter element around -30 of transcription start |
| *Solyc02g084680* | TATA-box | ATATAT | 761 | 6 | core promoter element around -30 of transcription start |
| *Solyc02g084680* | TATA-box | TATA | 762 | 4 | core promoter element around -30 of transcription start |
| *Solyc02g084680* | TATA-box | TATA | 853 | 4 | core promoter element around -30 of transcription start |
| *Solyc02g084680* | TATA-box | ATATAT | 1043 | 6 | core promoter element around -30 of transcription start |
| *Solyc02g084680* | TATA-box | TATA | 1044 | 4 | core promoter element around -30 of transcription start |
| *Solyc02g084680* | TATA-box | TATAAA | 1444 | 6 | core promoter element around -30 of transcription start |
| *Solyc02g084680* | TATA-box | TATAA | 1445 | 5 | core promoter element around -30 of transcription start |
| *Solyc02g084680* | TATA-box | TATA | 1446 | 4 | core promoter element around -30 of transcription start |
| *Solyc02g084680* | TATA-box | TATACA | 1486 | 6 | core promoter element around -30 of transcription start |
| *Solyc02g084680* | TATA-box | TATA | 1488 | 4 | core promoter element around -30 of transcription start |
| *Solyc02g084680* | TATA-box | TACAAAA | 1711 | 7 | core promoter element around -30 of transcription start |
| *Solyc02g084680* | TATA-box | TATATAA | 1795 | 7 | core promoter element around -30 of transcription start |
| *Solyc02g084680* | TATA-box | TATATA | 1796 | 6 | core promoter element around -30 of transcription start |
| *Solyc02g084680* | TATA-box | TATA | 1798 | 4 | core promoter element around -30 of transcription start |
| *Solyc02g084680* | TATA-box | ATTATA | 1854 | 6 | core promoter element around -30 of transcription start |
| *Solyc02g084680* | TATA-box | TATAA | 1855 | 5 | core promoter element around -30 of transcription start |
| *Solyc02g084680* | TATA-box | TATA | 1856 | 4 | core promoter element around -30 of transcription start |
| *Solyc02g084680* | TATC-box | TATCCCA | 767 | 7 | cis-acting element involved in gibberellin-responsiveness |
| *Solyc02g084680* | LTR | CCGAAA | 1623 | 6 | cis-acting element involved in low-temperature responsiveness |
| *Solyc02g084680* | TCCC-motif | TCTCCCT | 1882 | 7 | part of a light responsive element |
| *Solyc02g084680* | CAT-box | GCCACT | 901 | 6 | cis-acting regulatory element related to meristem expression |
| *Solyc02g084680* | ABRE | GACACGTGGC | 1293 | 9 | cis-acting element involved in the abscisic acid responsiveness |
| *Solyc02g084680* | ABRE | CACGTG | 1295 | 6 | cis-acting element involved in the abscisic acid responsiveness |
| *Solyc02g084680* | ABRE | ACGTG | 1296 | 5 | cis-acting element involved in the abscisic acid responsiveness |
| *Solyc02g084680* | TCA-element | CCATCTTTTT | 386 | 9 | cis-acting element involved in salicylic acid responsiveness |
| *Solyc02g084680* | TCA-element | CCATCTTTTT | 438 | 9 | cis-acting element involved in salicylic acid responsiveness |
| *Solyc02g084680* | TCA-element | TCAGAAGAGG | 470 | 9 | cis-acting element involved in salicylic acid responsiveness |
| *Solyc02g084680* | TCA-element | CCATCTTTTT | 1681 | 9 | cis-acting element involved in salicylic acid responsiveness |
| *Solyc03g033680* | G-box | TACGTG | 749 | 6 | cis-acting regulatory element involved in light responsiveness |
| *Solyc03g033680* | G-box | CACGAC | 1940 | 6 | cis-acting regulatory element involved in light responsiveness |
| *Solyc03g033680* | G-Box | CACGTT | 1016 | 6 | cis-acting regulatory element involved in light responsiveness |
| *Solyc03g033680* | CAAT-box | CAAAT | 11 | 5 | common cis-acting element in promoter and enhancer regions |
| *Solyc03g033680* | CAAT-box | CAAAT | 228 | 5 | common cis-acting element in promoter and enhancer regions |
| *Solyc03g033680* | CAAT-box | CAAAT | 235 | 5 | common cis-acting element in promoter and enhancer regions |
| *Solyc03g033680* | CAAT-box | CAAAT | 248 | 5 | common cis-acting element in promoter and enhancer regions |
| *Solyc03g033680* | CAAT-box | CAAAT | 306 | 5 | common cis-acting element in promoter and enhancer regions |
| *Solyc03g033680* | CAAT-box | CAAAT | 549 | 5 | common cis-acting element in promoter and enhancer regions |
| *Solyc03g033680* | CAAT-box | CAAAT | 561 | 5 | common cis-acting element in promoter and enhancer regions |
| *Solyc03g033680* | CAAT-box | CAAAT | 645 | 5 | common cis-acting element in promoter and enhancer regions |
| *Solyc03g033680* | CAAT-box | CAAAT | 850 | 5 | common cis-acting element in promoter and enhancer regions |
| *Solyc03g033680* | CAAT-box | CCAAT | 910 | 5 | common cis-acting element in promoter and enhancer regions |
| *Solyc03g033680* | CAAT-box | CAAAT | 1002 | 5 | common cis-acting element in promoter and enhancer regions |
| *Solyc03g033680* | CAAT-box | CCAAT | 1119 | 5 | common cis-acting element in promoter and enhancer regions |
| *Solyc03g033680* | CAAT-box | CAAAT | 1276 | 5 | common cis-acting element in promoter and enhancer regions |
| *Solyc03g033680* | CAAT-box | TGCCAAC | 1740 | 7 | common cis-acting element in promoter and enhancer regions |
| *Solyc03g033680* | Box 4 | ATTAAT | 160 | 6 | part of a conserved DNA module involved in light responsiveness |
| *Solyc03g033680* | GA-motif | ATAGATAA | 1470 | 8 | part of a light responsive element |
| *Solyc03g033680* | ARE | AAACCA | 1364 | 6 | cis-acting regulatory element essential for the anaerobic induction |
| *Solyc03g033680* | MBS | CAACTG | 411 | 6 | MYB binding site involved in drought-inducibility |
| *Solyc03g033680* | MBS | CAACTG | 840 | 6 | MYB binding site involved in drought-inducibility |
| *Solyc03g033680* | TATA-box | ATTATA | 85 | 6 | core promoter element around -30 of transcription start |
| *Solyc03g033680* | TATA-box | TATAA | 86 | 5 | core promoter element around -30 of transcription start |
| *Solyc03g033680* | TATA-box | TATA | 87 | 4 | core promoter element around -30 of transcription start |
| *Solyc03g033680* | TATA-box | TAAAGATT | 89 | 8 | core promoter element around -30 of transcription start |
| *Solyc03g033680* | TATA-box | ATTATA | 194 | 6 | core promoter element around -30 of transcription start |
| *Solyc03g033680* | TATA-box | TATAA | 195 | 5 | core promoter element around -30 of transcription start |
| *Solyc03g033680* | TATA-box | TATA | 196 | 4 | core promoter element around -30 of transcription start |
| *Solyc03g033680* | TATA-box | TATAA | 270 | 5 | core promoter element around -30 of transcription start |
| *Solyc03g033680* | TATA-box | TATA | 271 | 4 | core promoter element around -30 of transcription start |
| *Solyc03g033680* | TATA-box | TATTTAAA | 319 | 8 | core promoter element around -30 of transcription start |
| *Solyc03g033680* | TATA-box | TACAAAA | 376 | 7 | core promoter element around -30 of transcription start |
| *Solyc03g033680* | TATA-box | ATTATA | 392 | 6 | core promoter element around -30 of transcription start |
| *Solyc03g033680* | TATA-box | TATAA | 393 | 5 | core promoter element around -30 of transcription start |
| *Solyc03g033680* | TATA-box | TATA | 394 | 4 | core promoter element around -30 of transcription start |
| *Solyc03g033680* | TATA-box | TATA | 423 | 4 | core promoter element around -30 of transcription start |
| *Solyc03g033680* | TATA-box | TATAAAA | 506 | 7 | core promoter element around -30 of transcription start |
| *Solyc03g033680* | TATA-box | TATAAA | 507 | 6 | core promoter element around -30 of transcription start |
| *Solyc03g033680* | TATA-box | TATAA | 508 | 5 | core promoter element around -30 of transcription start |
| *Solyc03g033680* | TATA-box | TATA | 509 | 4 | core promoter element around -30 of transcription start |
| *Solyc03g033680* | TATA-box | ATATAA | 571 | 6 | core promoter element around -30 of transcription start |
| *Solyc03g033680* | TATA-box | TATA | 572 | 4 | core promoter element around -30 of transcription start |
| *Solyc03g033680* | TATA-box | TATAAAT | 638 | 7 | core promoter element around -30 of transcription start |
| *Solyc03g033680* | TATA-box | TATAAA | 639 | 6 | core promoter element around -30 of transcription start |
| *Solyc03g033680* | TATA-box | TATAA | 640 | 5 | core promoter element around -30 of transcription start |
| *Solyc03g033680* | TATA-box | TATA | 641 | 4 | core promoter element around -30 of transcription start |
| *Solyc03g033680* | TATA-box | TATAAAT | 660 | 7 | core promoter element around -30 of transcription start |
| *Solyc03g033680* | TATA-box | TATAAA | 661 | 6 | core promoter element around -30 of transcription start |
| *Solyc03g033680* | TATA-box | TATAA | 662 | 5 | core promoter element around -30 of transcription start |
| *Solyc03g033680* | TATA-box | TATA | 663 | 4 | core promoter element around -30 of transcription start |
| *Solyc03g033680* | TATA-box | TACAAAA | 665 | 7 | core promoter element around -30 of transcription start |
| *Solyc03g033680* | TATA-box | ATATAT | 679 | 6 | core promoter element around -30 of transcription start |
| *Solyc03g033680* | TATA-box | TATA | 680 | 4 | core promoter element around -30 of transcription start |
| *Solyc03g033680* | TATA-box | taTATAAAtc | 864 | 9 | core promoter element around -30 of transcription start |
| *Solyc03g033680* | TATA-box | TATAAAT | 865 | 7 | core promoter element around -30 of transcription start |
| *Solyc03g033680* | TATA-box | TATAAA | 866 | 6 | core promoter element around -30 of transcription start |
| *Solyc03g033680* | TATA-box | TATATAA | 867 | 7 | core promoter element around -30 of transcription start |
| *Solyc03g033680* | TATA-box | TATATA | 868 | 6 | core promoter element around -30 of transcription start |
| *Solyc03g033680* | TATA-box | ATATAT | 869 | 6 | core promoter element around -30 of transcription start |
| *Solyc03g033680* | TATA-box | TATA | 870 | 4 | core promoter element around -30 of transcription start |
| *Solyc03g033680* | TATA-box | ATTATA | 962 | 6 | core promoter element around -30 of transcription start |
| *Solyc03g033680* | TATA-box | TATAA | 963 | 5 | core promoter element around -30 of transcription start |
| *Solyc03g033680* | TATA-box | TATA | 964 | 4 | core promoter element around -30 of transcription start |
| *Solyc03g033680* | TATA-box | TACAAAA | 970 | 7 | core promoter element around -30 of transcription start |
| *Solyc03g033680* | TATA-box | ATTATA | 1011 | 6 | core promoter element around -30 of transcription start |
| *Solyc03g033680* | TATA-box | TATAA | 1012 | 5 | core promoter element around -30 of transcription start |
| *Solyc03g033680* | TATA-box | TATA | 1013 | 4 | core promoter element around -30 of transcription start |
| *Solyc03g033680* | TATA-box | TATAAAA | 1147 | 7 | core promoter element around -30 of transcription start |
| *Solyc03g033680* | TATA-box | TATAAA | 1148 | 6 | core promoter element around -30 of transcription start |
| *Solyc03g033680* | TATA-box | TATAA | 1149 | 5 | core promoter element around -30 of transcription start |
| *Solyc03g033680* | TATA-box | TATA | 1150 | 4 | core promoter element around -30 of transcription start |
| *Solyc03g033680* | TATA-box | ATTATA | 1179 | 6 | core promoter element around -30 of transcription start |
| *Solyc03g033680* | TATA-box | TATAA | 1180 | 5 | core promoter element around -30 of transcription start |
| *Solyc03g033680* | TATA-box | TATA | 1181 | 4 | core promoter element around -30 of transcription start |
| *Solyc03g033680* | TATA-box | TATA | 1210 | 4 | core promoter element around -30 of transcription start |
| *Solyc03g033680* | TATA-box | TATAAAT | 1298 | 7 | core promoter element around -30 of transcription start |
| *Solyc03g033680* | TATA-box | TATAAA | 1299 | 6 | core promoter element around -30 of transcription start |
| *Solyc03g033680* | TATA-box | TATAA | 1300 | 5 | core promoter element around -30 of transcription start |
| *Solyc03g033680* | TATA-box | TATA | 1301 | 4 | core promoter element around -30 of transcription start |
| *Solyc03g033680* | TATA-box | TATA | 1382 | 4 | core promoter element around -30 of transcription start |
| *Solyc03g033680* | TATA-box | ccTATAAAaa | 1446 | 9 | core promoter element around -30 of transcription start |
| *Solyc03g033680* | TATA-box | TATA | 1469 | 4 | core promoter element around -30 of transcription start |
| *Solyc03g033680* | TATA-box | TATATA | 1544 | 6 | core promoter element around -30 of transcription start |
| *Solyc03g033680* | TATA-box | ATATAA | 1545 | 6 | core promoter element around -30 of transcription start |
| *Solyc03g033680* | TATA-box | TATA | 1546 | 4 | core promoter element around -30 of transcription start |
| *Solyc03g033680* | TATA-box | ATTATA | 1573 | 6 | core promoter element around -30 of transcription start |
| *Solyc03g033680* | TATA-box | TATAA | 1574 | 5 | core promoter element around -30 of transcription start |
| *Solyc03g033680* | TATA-box | TATA | 1575 | 4 | core promoter element around -30 of transcription start |
| *Solyc03g033680* | TATA-box | TATATAA | 1774 | 7 | core promoter element around -30 of transcription start |
| *Solyc03g033680* | TATA-box | TATATA | 1775 | 6 | core promoter element around -30 of transcription start |
| *Solyc03g033680* | TATA-box | ATATAT | 1776 | 6 | core promoter element around -30 of transcription start |
| *Solyc03g033680* | TATA-box | TATATA | 1777 | 6 | core promoter element around -30 of transcription start |
| *Solyc03g033680* | TATA-box | ATATAT | 1778 | 6 | core promoter element around -30 of transcription start |
| *Solyc03g033680* | TATA-box | TATATA | 1779 | 6 | core promoter element around -30 of transcription start |
| *Solyc03g033680* | TATA-box | ATATAT | 1780 | 6 | core promoter element around -30 of transcription start |
| *Solyc03g033680* | TATA-box | TATATA | 1781 | 6 | core promoter element around -30 of transcription start |
| *Solyc03g033680* | TATA-box | ATATAT | 1782 | 6 | core promoter element around -30 of transcription start |
| *Solyc03g033680* | TATA-box | TATATA | 1783 | 6 | core promoter element around -30 of transcription start |
| *Solyc03g033680* | TATA-box | ATATAT | 1784 | 6 | core promoter element around -30 of transcription start |
| *Solyc03g033680* | TATA-box | TATATA | 1785 | 6 | core promoter element around -30 of transcription start |
| *Solyc03g033680* | TATA-box | ATATAT | 1786 | 6 | core promoter element around -30 of transcription start |
| *Solyc03g033680* | TATA-box | TATATA | 1787 | 6 | core promoter element around -30 of transcription start |
| *Solyc03g033680* | TATA-box | ATATAT | 1788 | 6 | core promoter element around -30 of transcription start |
| *Solyc03g033680* | TATA-box | TATATA | 1789 | 6 | core promoter element around -30 of transcription start |
| *Solyc03g033680* | TATA-box | ATATAA | 1790 | 6 | core promoter element around -30 of transcription start |
| *Solyc03g033680* | TATA-box | TATA | 1791 | 4 | core promoter element around -30 of transcription start |
| *Solyc03g033680* | TATA-box | TACAAAA | 1805 | 7 | core promoter element around -30 of transcription start |
| *Solyc03g033680* | TATA-box | TATA | 1934 | 4 | core promoter element around -30 of transcription start |
| *Solyc03g033680* | TATA-box | TATA | 1992 | 4 | core promoter element around -30 of transcription start |
| *Solyc03g033680* | LTR | CCGAAA | 879 | 6 | cis-acting element involved in low-temperature responsiveness |
| *Solyc03g033680* | GARE-motif | TCTGTTG | 1095 | 7 | gibberellin-responsive element |
| *Solyc03g033680* | TGACG-motif | TGACG | 894 | 5 | cis-acting regulatory element involved in the MeJA-responsiveness |
| *Solyc03g033680* | TGACG-motif | TGACG | 1305 | 5 | cis-acting regulatory element involved in the MeJA-responsiveness |
| *Solyc03g033680* | 3-AF1 binding site | TAAGAGAGGAA | 1657 | 10 | light responsive element |
| *Solyc03g033680* | TCCC-motif | TCTCCCT | 1682 | 7 | part of a light responsive element |
| *Solyc03g033680* | ABRE | ACGTG | 749 | 5 | cis-acting element involved in the abscisic acid responsiveness |
| *Solyc03g033680* | ABRE | ACGTG | 1017 | 5 | cis-acting element involved in the abscisic acid responsiveness |
| *Solyc03g033680* | I-box | TGATAATGT | 1483 | 9 | part of a light responsive element |
| *Solyc03g033680* | TCA-element | TCAGAAGAGG | 484 | 9 | cis-acting element involved in salicylic acid responsiveness |
| *Solyc03g033680* | CGTCA-motif | CGTCA | 894 | 5 | cis-acting regulatory element involved in the MeJA-responsiveness |
| *Solyc03g033680* | CGTCA-motif | CGTCA | 1305 | 5 | cis-acting regulatory element involved in the MeJA-responsiveness |
| *Solyc04g080970* | LTR | CCGAAA | 1007 | 6 | cis-acting element involved in low-temperature responsiveness |
| *Solyc04g080970* | TCT-motif | TCTTAC | 574 | 6 | part of a light responsive element |
| *Solyc04g080970* | TATA-box | ATATAT | 162 | 6 | core promoter element around -30 of transcription start |
| *Solyc04g080970* | TATA-box | TATATA | 163 | 6 | core promoter element around -30 of transcription start |
| *Solyc04g080970* | TATA-box | ATATAA | 164 | 6 | core promoter element around -30 of transcription start |
| *Solyc04g080970* | TATA-box | TATA | 165 | 4 | core promoter element around -30 of transcription start |
| *Solyc04g080970* | TATA-box | TATA | 177 | 4 | core promoter element around -30 of transcription start |
| *Solyc04g080970* | TATA-box | TATACA | 215 | 6 | core promoter element around -30 of transcription start |
| *Solyc04g080970* | TATA-box | TATA | 217 | 4 | core promoter element around -30 of transcription start |
| *Solyc04g080970* | TATA-box | TATA | 237 | 4 | core promoter element around -30 of transcription start |
| *Solyc04g080970* | TATA-box | TATA | 293 | 4 | core promoter element around -30 of transcription start |
| *Solyc04g080970* | TATA-box | TATAAAA | 339 | 7 | core promoter element around -30 of transcription start |
| *Solyc04g080970* | TATA-box | TATAAA | 340 | 6 | core promoter element around -30 of transcription start |
| *Solyc04g080970* | TATA-box | TATAA | 341 | 5 | core promoter element around -30 of transcription start |
| *Solyc04g080970* | TATA-box | TATA | 342 | 4 | core promoter element around -30 of transcription start |
| *Solyc04g080970* | TATA-box | TATA | 433 | 4 | core promoter element around -30 of transcription start |
| *Solyc04g080970* | TATA-box | TATAAAA | 582 | 7 | core promoter element around -30 of transcription start |
| *Solyc04g080970* | TATA-box | TATAAA | 583 | 6 | core promoter element around -30 of transcription start |
| *Solyc04g080970* | TATA-box | TATATAA | 584 | 7 | core promoter element around -30 of transcription start |
| *Solyc04g080970* | TATA-box | TATATA | 585 | 6 | core promoter element around -30 of transcription start |
| *Solyc04g080970* | TATA-box | TATA | 587 | 4 | core promoter element around -30 of transcription start |
| *Solyc04g080970* | TATA-box | ATTATA | 674 | 6 | core promoter element around -30 of transcription start |
| *Solyc04g080970* | TATA-box | TATAA | 675 | 5 | core promoter element around -30 of transcription start |
| *Solyc04g080970* | TATA-box | TATA | 676 | 4 | core promoter element around -30 of transcription start |
| *Solyc04g080970* | TATA-box | TATA | 689 | 4 | core promoter element around -30 of transcription start |
| *Solyc04g080970* | TATA-box | TATAA | 698 | 5 | core promoter element around -30 of transcription start |
| *Solyc04g080970* | TATA-box | TATA | 699 | 4 | core promoter element around -30 of transcription start |
| *Solyc04g080970* | TATA-box | TATACA | 708 | 6 | core promoter element around -30 of transcription start |
| *Solyc04g080970* | TATA-box | TATA | 710 | 4 | core promoter element around -30 of transcription start |
| *Solyc04g080970* | TATA-box | TATAAAA | 745 | 7 | core promoter element around -30 of transcription start |
| *Solyc04g080970* | TATA-box | TATAAA | 746 | 6 | core promoter element around -30 of transcription start |
| *Solyc04g080970* | TATA-box | TATAA | 747 | 5 | core promoter element around -30 of transcription start |
| *Solyc04g080970* | TATA-box | TATA | 748 | 4 | core promoter element around -30 of transcription start |
| *Solyc04g080970* | TATA-box | TATATA | 759 | 6 | core promoter element around -30 of transcription start |
| *Solyc04g080970* | TATA-box | TATA | 761 | 4 | core promoter element around -30 of transcription start |
| *Solyc04g080970* | TATA-box | TACAAAA | 763 | 7 | core promoter element around -30 of transcription start |
| *Solyc04g080970* | TATA-box | ATTATA | 783 | 6 | core promoter element around -30 of transcription start |
| *Solyc04g080970* | TATA-box | TATAA | 784 | 5 | core promoter element around -30 of transcription start |
| *Solyc04g080970* | TATA-box | TATA | 785 | 4 | core promoter element around -30 of transcription start |
| *Solyc04g080970* | TATA-box | ATTATA | 832 | 6 | core promoter element around -30 of transcription start |
| *Solyc04g080970* | TATA-box | TATAA | 833 | 5 | core promoter element around -30 of transcription start |
| *Solyc04g080970* | TATA-box | TATA | 834 | 4 | core promoter element around -30 of transcription start |
| *Solyc04g080970* | TATA-box | TATAAA | 930 | 6 | core promoter element around -30 of transcription start |
| *Solyc04g080970* | TATA-box | TATAA | 931 | 5 | core promoter element around -30 of transcription start |
| *Solyc04g080970* | TATA-box | TATA | 932 | 4 | core promoter element around -30 of transcription start |
| *Solyc04g080970* | TATA-box | ATATAT | 1065 | 6 | core promoter element around -30 of transcription start |
| *Solyc04g080970* | TATA-box | TATATA | 1066 | 6 | core promoter element around -30 of transcription start |
| *Solyc04g080970* | TATA-box | ATATAT | 1067 | 6 | core promoter element around -30 of transcription start |
| *Solyc04g080970* | TATA-box | TATA | 1068 | 4 | core promoter element around -30 of transcription start |
| *Solyc04g080970* | TATA-box | TATTTAAA | 1093 | 8 | core promoter element around -30 of transcription start |
| *Solyc04g080970* | TATA-box | ATTATA | 1280 | 6 | core promoter element around -30 of transcription start |
| *Solyc04g080970* | TATA-box | TATAA | 1281 | 5 | core promoter element around -30 of transcription start |
| *Solyc04g080970* | TATA-box | TATA | 1282 | 4 | core promoter element around -30 of transcription start |
| *Solyc04g080970* | TATA-box | TATA | 1845 | 4 | core promoter element around -30 of transcription start |
| *Solyc04g080970* | TATA-box | TATA | 1959 | 4 | core promoter element around -30 of transcription start |
| *Solyc04g080970* | MBS | CAACTG | 1512 | 6 | MYB binding site involved in drought-inducibility |
| *Solyc04g080970* | Box II | CCACGTGGC | 1432 | 9 | part of a light responsive element |
| *Solyc04g080970* | G-box | TACGTG | 548 | 6 | cis-acting regulatory element involved in light responsiveness |
| *Solyc04g080970* | G-box | CACGTC | 982 | 6 | cis-acting regulatory element involved in light responsiveness |
| *Solyc04g080970* | G-box | GCCACGTGGA | 1432 | 10 | cis-acting regulatory element involved in light responsiveness |
| *Solyc04g080970* | G-box | CACGTG | 1434 | 6 | cis-acting regulatory element involved in light responsiveness |
| *Solyc04g080970* | G-Box | CACGTG | 1434 | 6 | cis-acting regulatory element involved in light responsiveness |
| *Solyc04g080970* | CAAT-box | CAAAT | 85 | 5 | common cis-acting element in promoter and enhancer regions |
| *Solyc04g080970* | CAAT-box | CCAAT | 192 | 5 | common cis-acting element in promoter and enhancer regions |
| *Solyc04g080970* | CAAT-box | CAAAT | 448 | 5 | common cis-acting element in promoter and enhancer regions |
| *Solyc04g080970* | CAAT-box | CAAAT | 720 | 5 | common cis-acting element in promoter and enhancer regions |
| *Solyc04g080970* | CAAT-box | CAAAT | 867 | 5 | common cis-acting element in promoter and enhancer regions |
| *Solyc04g080970* | CAAT-box | CCAAT | 993 | 5 | common cis-acting element in promoter and enhancer regions |
| *Solyc04g080970* | CAAT-box | CAAAT | 1204 | 5 | common cis-acting element in promoter and enhancer regions |
| *Solyc04g080970* | CAAT-box | CAAAT | 1234 | 5 | common cis-acting element in promoter and enhancer regions |
| *Solyc04g080970* | CAAT-box | CCAAT | 1293 | 5 | common cis-acting element in promoter and enhancer regions |
| *Solyc04g080970* | CAAT-box | CAAAT | 1546 | 5 | common cis-acting element in promoter and enhancer regions |
| *Solyc04g080970* | CAAT-box | CAAAT | 1637 | 5 | common cis-acting element in promoter and enhancer regions |
| *Solyc04g080970* | CAAT-box | CAAAT | 1769 | 5 | common cis-acting element in promoter and enhancer regions |
| *Solyc04g080970* | CAAT-box | CAAAT | 1806 | 5 | common cis-acting element in promoter and enhancer regions |
| *Solyc04g080970* | CAAT-box | CCAAT | 1876 | 5 | common cis-acting element in promoter and enhancer regions |
| *Solyc04g080970* | CAAT-box | CAAAT | 1881 | 5 | common cis-acting element in promoter and enhancer regions |
| *Solyc04g080970* | CAAT-box | CAAAT | 1982 | 5 | common cis-acting element in promoter and enhancer regions |
| *Solyc04g080970* | Box 4 | ATTAAT | 207 | 6 | part of a conserved DNA module involved in light responsiveness |
| *Solyc04g080970* | Box 4 | ATTAAT | 828 | 6 | part of a conserved DNA module involved in light responsiveness |
| *Solyc04g080970* | Box 4 | ATTAAT | 1181 | 6 | part of a conserved DNA module involved in light responsiveness |
| *Solyc04g080970* | Box 4 | ATTAAT | 1194 | 6 | part of a conserved DNA module involved in light responsiveness |
| *Solyc04g080970* | Box 4 | ATTAAT | 1300 | 6 | part of a conserved DNA module involved in light responsiveness |
| *Solyc04g080970* | CGTCA-motif | CGTCA | 1059 | 5 | cis-acting regulatory element involved in the MeJA-responsiveness |
| *Solyc04g080970* | TCA-element | CCATCTTTTT | 1656 | 9 | cis-acting element involved in salicylic acid responsiveness |
| *Solyc04g080970* | ABRE | ACGTG | 549 | 5 | cis-acting element involved in the abscisic acid responsiveness |
| *Solyc04g080970* | ABRE | ACGTG | 982 | 5 | cis-acting element involved in the abscisic acid responsiveness |
| *Solyc04g080970* | ABRE | CACGTG | 1434 | 6 | cis-acting element involved in the abscisic acid responsiveness |
| *Solyc04g080970* | ABRE | ACGTG | 1435 | 5 | cis-acting element involved in the abscisic acid responsiveness |
| *Solyc04g080970* | AE-box | AGAAACAA | 27 | 8 | part of a module for light response |
| *Solyc04g080970* | CAT-box | GCCACT | 970 | 6 | cis-acting regulatory element related to meristem expression |
| *Solyc04g080970* | CAT-box | GCCACT | 1705 | 6 | cis-acting regulatory element related to meristem expression |
| *Solyc04g080970* | TGACG-motif | TGACG | 1059 | 5 | cis-acting regulatory element involved in the MeJA-responsiveness |
| *Solyc08g077450* | ATCT-motif | AATCTAATCC | 235 | 9 | part of a conserved DNA module involved in light responsiveness |
| *Solyc08g077450* | ATCT-motif | AATCTAATCC | 240 | 9 | part of a conserved DNA module involved in light responsiveness |
| *Solyc08g077450* | ATCT-motif | AATCTAATCC | 339 | 9 | part of a conserved DNA module involved in light responsiveness |
| *Solyc08g077450* | 3-AF1 binding site | TAAGAGAGGAA | 1433 | 10 | light responsive element |
| *Solyc08g077450* | TCCC-motif | TCTCCCT | 1925 | 7 | part of a light responsive element |
| *Solyc08g077450* | ABRE | ACGTG | 1688 | 5 | cis-acting element involved in the abscisic acid responsiveness |
| *Solyc08g077450* | TCT-motif | TCTTAC | 1652 | 6 | part of a light responsive element |
| *Solyc08g077450* | GT1-motif | GGTTAA | 283 | 6 | light responsive element |
| *Solyc08g077450* | GT1-motif | GGTTAA | 1908 | 6 | light responsive element |
| *Solyc08g077450* | G-box | TACGTG | 1687 | 6 | cis-acting regulatory element involved in light responsiveness |
| *Solyc08g077450* | CAAT-box | CAAAT | 169 | 5 | common cis-acting element in promoter and enhancer regions |
| *Solyc08g077450* | CAAT-box | CCAAT | 237 | 5 | common cis-acting element in promoter and enhancer regions |
| *Solyc08g077450* | CAAT-box | CAAAT | 247 | 5 | common cis-acting element in promoter and enhancer regions |
| *Solyc08g077450* | CAAT-box | CCAAT | 371 | 5 | common cis-acting element in promoter and enhancer regions |
| *Solyc08g077450* | CAAT-box | CAAAT | 517 | 5 | common cis-acting element in promoter and enhancer regions |
| *Solyc08g077450* | CAAT-box | CCCAATTT | 518 | 8 | common cis-acting element in promoter and enhancer regions |
| *Solyc08g077450* | CAAT-box | CCAAT | 520 | 5 | common cis-acting element in promoter and enhancer regions |
| *Solyc08g077450* | CAAT-box | CAAAT | 588 | 5 | common cis-acting element in promoter and enhancer regions |
| *Solyc08g077450* | CAAT-box | CCAAT | 629 | 5 | common cis-acting element in promoter and enhancer regions |
| *Solyc08g077450* | CAAT-box | CCAAT | 829 | 5 | common cis-acting element in promoter and enhancer regions |
| *Solyc08g077450* | CAAT-box | CAAAT | 901 | 5 | common cis-acting element in promoter and enhancer regions |
| *Solyc08g077450* | CAAT-box | CAAAT | 904 | 5 | common cis-acting element in promoter and enhancer regions |
| *Solyc08g077450* | CAAT-box | CAAAT | 910 | 5 | common cis-acting element in promoter and enhancer regions |
| *Solyc08g077450* | CAAT-box | CAAAT | 933 | 5 | common cis-acting element in promoter and enhancer regions |
| *Solyc08g077450* | CAAT-box | CAAAT | 1028 | 5 | common cis-acting element in promoter and enhancer regions |
| *Solyc08g077450* | CAAT-box | CCAAT | 1106 | 5 | common cis-acting element in promoter and enhancer regions |
| *Solyc08g077450* | CAAT-box | CAAAT | 1122 | 5 | common cis-acting element in promoter and enhancer regions |
| *Solyc08g077450* | CAAT-box | CAAAT | 1267 | 5 | common cis-acting element in promoter and enhancer regions |
| *Solyc08g077450* | CAAT-box | CAAAT | 1323 | 5 | common cis-acting element in promoter and enhancer regions |
| *Solyc08g077450* | CAAT-box | CAAAT | 1423 | 5 | common cis-acting element in promoter and enhancer regions |
| *Solyc08g077450* | CAAT-box | CCAAT | 1596 | 5 | common cis-acting element in promoter and enhancer regions |
| *Solyc08g077450* | CAAT-box | CCAAT | 1615 | 5 | common cis-acting element in promoter and enhancer regions |
| *Solyc08g077450* | CAAT-box | CAAAT | 1711 | 5 | common cis-acting element in promoter and enhancer regions |
| *Solyc08g077450* | CAAT-box | CCAAT | 1729 | 5 | common cis-acting element in promoter and enhancer regions |
| *Solyc08g077450* | CAAT-box | CCAAT | 1837 | 5 | common cis-acting element in promoter and enhancer regions |
| *Solyc08g077450* | CAAT-box | CAAAT | 1848 | 5 | common cis-acting element in promoter and enhancer regions |
| *Solyc08g077450* | Box 4 | ATTAAT | 1049 | 6 | part of a conserved DNA module involved in light responsiveness |
| *Solyc08g077450* | TC-rich repeats | GTTTTCTTAC | 1081 | 9 | cis-acting element involved in defense and stress responsiveness |
| *Solyc08g077450* | MRE | AACCTAA | 818 | 7 | MYB binding site involved in light responsiveness |
| *Solyc08g077450* | MRE | AACCTAA | 1171 | 7 | MYB binding site involved in light responsiveness |
| *Solyc08g077450* | ARE | AAACCA | 1804 | 6 | cis-acting regulatory element essential for the anaerobic induction |
| *Solyc08g077450* | P-box | CCTTTTG | 1873 | 7 | gibberellin-responsive element |
| *Solyc08g077450* | TATA-box | ATATAT | 52 | 6 | core promoter element around -30 of transcription start |
| *Solyc08g077450* | TATA-box | TATATA | 53 | 6 | core promoter element around -30 of transcription start |
| *Solyc08g077450* | TATA-box | ATATAT | 54 | 6 | core promoter element around -30 of transcription start |
| *Solyc08g077450* | TATA-box | TATA | 55 | 4 | core promoter element around -30 of transcription start |
| *Solyc08g077450* | TATA-box | TATTTAAA | 70 | 8 | core promoter element around -30 of transcription start |
| *Solyc08g077450* | TATA-box | TATATTTATATTT | 75 | 12 | core promoter element around -30 of transcription start |
| *Solyc08g077450* | TATA-box | ATATAA | 83 | 6 | core promoter element around -30 of transcription start |
| *Solyc08g077450* | TATA-box | TATA | 84 | 4 | core promoter element around -30 of transcription start |
| *Solyc08g077450* | TATA-box | TATAAA | 178 | 6 | core promoter element around -30 of transcription start |
| *Solyc08g077450* | TATA-box | TATAA | 179 | 5 | core promoter element around -30 of transcription start |
| *Solyc08g077450* | TATA-box | TATA | 180 | 4 | core promoter element around -30 of transcription start |
| *Solyc08g077450* | TATA-box | ATATAA | 189 | 6 | core promoter element around -30 of transcription start |
| *Solyc08g077450* | TATA-box | TATA | 190 | 4 | core promoter element around -30 of transcription start |
| *Solyc08g077450* | TATA-box | TATATAA | 202 | 7 | core promoter element around -30 of transcription start |
| *Solyc08g077450* | TATA-box | TATATA | 203 | 6 | core promoter element around -30 of transcription start |
| *Solyc08g077450* | TATA-box | ATATAT | 204 | 6 | core promoter element around -30 of transcription start |
| *Solyc08g077450* | TATA-box | TATATA | 205 | 6 | core promoter element around -30 of transcription start |
| *Solyc08g077450* | TATA-box | ATATAT | 206 | 6 | core promoter element around -30 of transcription start |
| *Solyc08g077450* | TATA-box | TATATA | 207 | 6 | core promoter element around -30 of transcription start |
| *Solyc08g077450* | TATA-box | ATATAT | 208 | 6 | core promoter element around -30 of transcription start |
| *Solyc08g077450* | TATA-box | TATATA | 209 | 6 | core promoter element around -30 of transcription start |
| *Solyc08g077450* | TATA-box | ATATAT | 210 | 6 | core promoter element around -30 of transcription start |
| *Solyc08g077450* | TATA-box | TATA | 211 | 4 | core promoter element around -30 of transcription start |
| *Solyc08g077450* | TATA-box | TATAAAA | 407 | 7 | core promoter element around -30 of transcription start |
| *Solyc08g077450* | TATA-box | TATAAA | 408 | 6 | core promoter element around -30 of transcription start |
| *Solyc08g077450* | TATA-box | TATAA | 409 | 5 | core promoter element around -30 of transcription start |
| *Solyc08g077450* | TATA-box | TATA | 410 | 4 | core promoter element around -30 of transcription start |
| *Solyc08g077450* | TATA-box | ATTATA | 445 | 6 | core promoter element around -30 of transcription start |
| *Solyc08g077450* | TATA-box | TATAA | 446 | 5 | core promoter element around -30 of transcription start |
| *Solyc08g077450* | TATA-box | TATA | 447 | 4 | core promoter element around -30 of transcription start |
| *Solyc08g077450* | TATA-box | TATA | 453 | 4 | core promoter element around -30 of transcription start |
| *Solyc08g077450* | TATA-box | ATATAA | 462 | 6 | core promoter element around -30 of transcription start |
| *Solyc08g077450* | TATA-box | TATA | 463 | 4 | core promoter element around -30 of transcription start |
| *Solyc08g077450* | TATA-box | ATATAT | 491 | 6 | core promoter element around -30 of transcription start |
| *Solyc08g077450* | TATA-box | TATA | 492 | 4 | core promoter element around -30 of transcription start |
| *Solyc08g077450* | TATA-box | TATAA | 568 | 5 | core promoter element around -30 of transcription start |
| *Solyc08g077450* | TATA-box | TATA | 569 | 4 | core promoter element around -30 of transcription start |
| *Solyc08g077450* | TATA-box | TATAA | 648 | 5 | core promoter element around -30 of transcription start |
| *Solyc08g077450* | TATA-box | TATA | 649 | 4 | core promoter element around -30 of transcription start |
| *Solyc08g077450* | TATA-box | TATAAAT | 674 | 7 | core promoter element around -30 of transcription start |
| *Solyc08g077450* | TATA-box | TATAAA | 675 | 6 | core promoter element around -30 of transcription start |
| *Solyc08g077450* | TATA-box | TATAA | 676 | 5 | core promoter element around -30 of transcription start |
| *Solyc08g077450* | TATA-box | TATA | 677 | 4 | core promoter element around -30 of transcription start |
| *Solyc08g077450* | TATA-box | TATTTAAA | 729 | 8 | core promoter element around -30 of transcription start |
| *Solyc08g077450* | TATA-box | ATATAT | 968 | 6 | core promoter element around -30 of transcription start |
| *Solyc08g077450* | TATA-box | TATATA | 969 | 6 | core promoter element around -30 of transcription start |
| *Solyc08g077450* | TATA-box | ATATAA | 970 | 6 | core promoter element around -30 of transcription start |
| *Solyc08g077450* | TATA-box | TATA | 971 | 4 | core promoter element around -30 of transcription start |
| *Solyc08g077450* | TATA-box | TATAAATA | 983 | 8 | core promoter element around -30 of transcription start |
| *Solyc08g077450* | TATA-box | TATAAAT | 984 | 7 | core promoter element around -30 of transcription start |
| *Solyc08g077450* | TATA-box | TATAAA | 985 | 6 | core promoter element around -30 of transcription start |
| *Solyc08g077450* | TATA-box | TATAA | 986 | 5 | core promoter element around -30 of transcription start |
| *Solyc08g077450* | TATA-box | TATA | 987 | 4 | core promoter element around -30 of transcription start |
| *Solyc08g077450* | TATA-box | TATA | 1110 | 4 | core promoter element around -30 of transcription start |
| *Solyc08g077450* | TATA-box | ATTATA | 1130 | 6 | core promoter element around -30 of transcription start |
| *Solyc08g077450* | TATA-box | TATAA | 1131 | 5 | core promoter element around -30 of transcription start |
| *Solyc08g077450* | TATA-box | TATA | 1132 | 4 | core promoter element around -30 of transcription start |
| *Solyc08g077450* | TATA-box | TATA | 1138 | 4 | core promoter element around -30 of transcription start |
| *Solyc08g077450* | TATA-box | taTATAAAtc | 1141 | 9 | core promoter element around -30 of transcription start |
| *Solyc08g077450* | TATA-box | TATAAAT | 1142 | 7 | core promoter element around -30 of transcription start |
| *Solyc08g077450* | TATA-box | TATAAA | 1143 | 6 | core promoter element around -30 of transcription start |
| *Solyc08g077450* | TATA-box | TATATAA | 1144 | 7 | core promoter element around -30 of transcription start |
| *Solyc08g077450* | TATA-box | TATATA | 1145 | 6 | core promoter element around -30 of transcription start |
| *Solyc08g077450* | TATA-box | ATATAA | 1146 | 6 | core promoter element around -30 of transcription start |
| *Solyc08g077450* | TATA-box | TATA | 1147 | 4 | core promoter element around -30 of transcription start |
| *Solyc08g077450* | TATA-box | ATATAT | 1200 | 6 | core promoter element around -30 of transcription start |
| *Solyc08g077450* | TATA-box | TATATA | 1201 | 6 | core promoter element around -30 of transcription start |
| *Solyc08g077450* | TATA-box | TATA | 1203 | 4 | core promoter element around -30 of transcription start |
| *Solyc08g077450* | TATA-box | TACAAAA | 1205 | 7 | core promoter element around -30 of transcription start |
| *Solyc08g077450* | TATA-box | TATACA | 1220 | 6 | core promoter element around -30 of transcription start |
| *Solyc08g077450* | TATA-box | TATA | 1222 | 4 | core promoter element around -30 of transcription start |
| *Solyc08g077450* | TATA-box | ATATAT | 1226 | 6 | core promoter element around -30 of transcription start |
| *Solyc08g077450* | TATA-box | TATA | 1227 | 4 | core promoter element around -30 of transcription start |
| *Solyc08g077450* | TATA-box | TATA | 1237 | 4 | core promoter element around -30 of transcription start |
| *Solyc08g077450* | TATA-box | TATA | 1243 | 4 | core promoter element around -30 of transcription start |
| *Solyc08g077450* | TATA-box | TACAAAA | 1245 | 7 | core promoter element around -30 of transcription start |
| *Solyc08g077450* | TATA-box | TATACA | 1261 | 6 | core promoter element around -30 of transcription start |
| *Solyc08g077450* | TATA-box | TATA | 1263 | 4 | core promoter element around -30 of transcription start |
| *Solyc08g077450* | TATA-box | TATAAAA | 1274 | 7 | core promoter element around -30 of transcription start |
| *Solyc08g077450* | TATA-box | TATAAA | 1275 | 6 | core promoter element around -30 of transcription start |
| *Solyc08g077450* | TATA-box | TATAA | 1276 | 5 | core promoter element around -30 of transcription start |
| *Solyc08g077450* | TATA-box | TATA | 1277 | 4 | core promoter element around -30 of transcription start |
| *Solyc08g077450* | TATA-box | TATACA | 1326 | 6 | core promoter element around -30 of transcription start |
| *Solyc08g077450* | TATA-box | TATA | 1328 | 4 | core promoter element around -30 of transcription start |
| *Solyc08g077450* | TATA-box | TATAAAT | 1362 | 7 | core promoter element around -30 of transcription start |
| *Solyc08g077450* | TATA-box | TATAAA | 1363 | 6 | core promoter element around -30 of transcription start |
| *Solyc08g077450* | TATA-box | TATAA | 1364 | 5 | core promoter element around -30 of transcription start |
| *Solyc08g077450* | TATA-box | TATA | 1365 | 4 | core promoter element around -30 of transcription start |
| *Solyc08g077450* | TATA-box | TATACA | 1371 | 6 | core promoter element around -30 of transcription start |
| *Solyc08g077450* | TATA-box | TATATA | 1373 | 6 | core promoter element around -30 of transcription start |
| *Solyc08g077450* | TATA-box | TATA | 1375 | 4 | core promoter element around -30 of transcription start |
| *Solyc08g077450* | TATA-box | ATATAT | 1413 | 6 | core promoter element around -30 of transcription start |
| *Solyc08g077450* | TATA-box | TATATTTATATTT | 1414 | 12 | core promoter element around -30 of transcription start |
| *Solyc08g077450* | TATA-box | ATATAT | 1415 | 6 | core promoter element around -30 of transcription start |
| *Solyc08g077450* | TATA-box | TATATA | 1416 | 6 | core promoter element around -30 of transcription start |
| *Solyc08g077450* | TATA-box | ATATAT | 1417 | 6 | core promoter element around -30 of transcription start |
| *Solyc08g077450* | TATA-box | TATATA | 1418 | 6 | core promoter element around -30 of transcription start |
| *Solyc08g077450* | TATA-box | ATATAT | 1419 | 6 | core promoter element around -30 of transcription start |
| *Solyc08g077450* | TATA-box | TATA | 1420 | 4 | core promoter element around -30 of transcription start |
| *Solyc08g077450* | TATA-box | ATATAT | 1460 | 6 | core promoter element around -30 of transcription start |
| *Solyc08g077450* | TATA-box | TATA | 1461 | 4 | core promoter element around -30 of transcription start |
| *Solyc08g077450* | TATA-box | TATACA | 1465 | 6 | core promoter element around -30 of transcription start |
| *Solyc08g077450* | TATA-box | TATATA | 1467 | 6 | core promoter element around -30 of transcription start |
| *Solyc08g077450* | TATA-box | ATATAT | 1468 | 6 | core promoter element around -30 of transcription start |
| *Solyc08g077450* | TATA-box | TATATA | 1469 | 6 | core promoter element around -30 of transcription start |
| *Solyc08g077450* | TATA-box | ATATAT | 1470 | 6 | core promoter element around -30 of transcription start |
| *Solyc08g077450* | TATA-box | TATATA | 1471 | 6 | core promoter element around -30 of transcription start |
| *Solyc08g077450* | TATA-box | ATATAA | 1472 | 6 | core promoter element around -30 of transcription start |
| *Solyc08g077450* | TATA-box | TATA | 1473 | 4 | core promoter element around -30 of transcription start |
| *Solyc08g077450* | TATA-box | TATAAAA | 1488 | 7 | core promoter element around -30 of transcription start |
| *Solyc08g077450* | TATA-box | TATAAA | 1489 | 6 | core promoter element around -30 of transcription start |
| *Solyc08g077450* | TATA-box | TATAA | 1490 | 5 | core promoter element around -30 of transcription start |
| *Solyc08g077450* | TATA-box | TATA | 1491 | 4 | core promoter element around -30 of transcription start |
| *Solyc08g077450* | TATA-box | TACAAAA | 1508 | 7 | core promoter element around -30 of transcription start |
| *Solyc08g077450* | TATA-box | TATACA | 1511 | 6 | core promoter element around -30 of transcription start |
| *Solyc08g077450* | TATA-box | TATA | 1513 | 4 | core promoter element around -30 of transcription start |
| *Solyc08g077450* | TATA-box | TATA | 1548 | 4 | core promoter element around -30 of transcription start |
| *Solyc08g077450* | TATA-box | TATA | 1749 | 4 | core promoter element around -30 of transcription start |
| *Solyc08g077450* | TATA-box | ATATAA | 1767 | 6 | core promoter element around -30 of transcription start |
| *Solyc08g077450* | TATA-box | TATA | 1768 | 4 | core promoter element around -30 of transcription start |
| *Solyc08g077450* | TATA-box | TATA | 1787 | 4 | core promoter element around -30 of transcription start |
| *Solyc08g077450* | TATA-box | ATATAA | 1799 | 6 | core promoter element around -30 of transcription start |
| *Solyc08g077450* | TATA-box | TATA | 1800 | 4 | core promoter element around -30 of transcription start |
| *Solyc08g077450* | TATA-box | TATAA | 1822 | 5 | core promoter element around -30 of transcription start |
| *Solyc08g077450* | TATA-box | TATA | 1823 | 4 | core promoter element around -30 of transcription start |
| *Solyc10g054070* | TATA-box | TATAAAA | 21 | 7 | core promoter element around -30 of transcription start |
| *Solyc10g054070* | TATA-box | TATAAA | 22 | 6 | core promoter element around -30 of transcription start |
| *Solyc10g054070* | TATA-box | TATAA | 23 | 5 | core promoter element around -30 of transcription start |
| *Solyc10g054070* | TATA-box | TATA | 24 | 4 | core promoter element around -30 of transcription start |
| *Solyc10g054070* | TATA-box | TATA | 62 | 4 | core promoter element around -30 of transcription start |
| *Solyc10g054070* | TATA-box | ATATAA | 84 | 6 | core promoter element around -30 of transcription start |
| *Solyc10g054070* | TATA-box | TATA | 85 | 4 | core promoter element around -30 of transcription start |
| *Solyc10g054070* | TATA-box | ATATAT | 162 | 6 | core promoter element around -30 of transcription start |
| *Solyc10g054070* | TATA-box | TATA | 163 | 4 | core promoter element around -30 of transcription start |
| *Solyc10g054070* | TATA-box | ATTATA | 194 | 6 | core promoter element around -30 of transcription start |
| *Solyc10g054070* | TATA-box | TATAA | 195 | 5 | core promoter element around -30 of transcription start |
| *Solyc10g054070* | TATA-box | TATA | 196 | 4 | core promoter element around -30 of transcription start |
| *Solyc10g054070* | TATA-box | taTATAAAtc | 231 | 9 | core promoter element around -30 of transcription start |
| *Solyc10g054070* | TATA-box | TATACA | 235 | 6 | core promoter element around -30 of transcription start |
| *Solyc10g054070* | TATA-box | TATA | 237 | 4 | core promoter element around -30 of transcription start |
| *Solyc10g054070* | TATA-box | ATTATA | 512 | 6 | core promoter element around -30 of transcription start |
| *Solyc10g054070* | TATA-box | TATAA | 513 | 5 | core promoter element around -30 of transcription start |
| *Solyc10g054070* | TATA-box | TATA | 514 | 4 | core promoter element around -30 of transcription start |
| *Solyc10g054070* | TATA-box | TATAAAT | 518 | 7 | core promoter element around -30 of transcription start |
| *Solyc10g054070* | TATA-box | TATAAA | 519 | 6 | core promoter element around -30 of transcription start |
| *Solyc10g054070* | TATA-box | TATAA | 520 | 5 | core promoter element around -30 of transcription start |
| *Solyc10g054070* | TATA-box | TATA | 521 | 4 | core promoter element around -30 of transcription start |
| *Solyc10g054070* | TATA-box | ATTATA | 636 | 6 | core promoter element around -30 of transcription start |
| *Solyc10g054070* | TATA-box | TATAA | 637 | 5 | core promoter element around -30 of transcription start |
| *Solyc10g054070* | TATA-box | TATA | 638 | 4 | core promoter element around -30 of transcription start |
| *Solyc10g054070* | TATA-box | ATTATA | 701 | 6 | core promoter element around -30 of transcription start |
| *Solyc10g054070* | TATA-box | TATAA | 702 | 5 | core promoter element around -30 of transcription start |
| *Solyc10g054070* | TATA-box | TATA | 703 | 4 | core promoter element around -30 of transcription start |
| *Solyc10g054070* | TATA-box | TATAA | 762 | 5 | core promoter element around -30 of transcription start |
| *Solyc10g054070* | TATA-box | TATA | 763 | 4 | core promoter element around -30 of transcription start |
| *Solyc10g054070* | TATA-box | TATA | 869 | 4 | core promoter element around -30 of transcription start |
| *Solyc10g054070* | TATA-box | ATATAT | 907 | 6 | core promoter element around -30 of transcription start |
| *Solyc10g054070* | TATA-box | TATATA | 908 | 6 | core promoter element around -30 of transcription start |
| *Solyc10g054070* | TATA-box | TATA | 910 | 4 | core promoter element around -30 of transcription start |
| *Solyc10g054070* | TATA-box | TATTTAAA | 926 | 8 | core promoter element around -30 of transcription start |
| *Solyc10g054070* | TATA-box | taTATAAAtc | 928 | 9 | core promoter element around -30 of transcription start |
| *Solyc10g054070* | TATA-box | TAAAGATT | 934 | 8 | core promoter element around -30 of transcription start |
| *Solyc10g054070* | TATA-box | TATTTAAA | 938 | 8 | core promoter element around -30 of transcription start |
| *Solyc10g054070* | TATA-box | TATATTTATATTT | 941 | 12 | core promoter element around -30 of transcription start |
| *Solyc10g054070* | TATA-box | ATATAA | 943 | 6 | core promoter element around -30 of transcription start |
| *Solyc10g054070* | TATA-box | TATA | 944 | 4 | core promoter element around -30 of transcription start |
| *Solyc10g054070* | TATA-box | ATATAA | 974 | 6 | core promoter element around -30 of transcription start |
| *Solyc10g054070* | TATA-box | TATA | 975 | 4 | core promoter element around -30 of transcription start |
| *Solyc10g054070* | TATA-box | ATTATA | 979 | 6 | core promoter element around -30 of transcription start |
| *Solyc10g054070* | TATA-box | TATAA | 980 | 5 | core promoter element around -30 of transcription start |
| *Solyc10g054070* | TATA-box | TATA | 981 | 4 | core promoter element around -30 of transcription start |
| *Solyc10g054070* | TATA-box | taTATAAAg | 1094 | 9 | core promoter element around -30 of transcription start |
| *Solyc10g054070* | TATA-box | TATAAA | 1095 | 6 | core promoter element around -30 of transcription start |
| *Solyc10g054070* | TATA-box | TATATAA | 1096 | 7 | core promoter element around -30 of transcription start |
| *Solyc10g054070* | TATA-box | TATATA | 1097 | 6 | core promoter element around -30 of transcription start |
| *Solyc10g054070* | TATA-box | TATA | 1099 | 4 | core promoter element around -30 of transcription start |
| *Solyc10g054070* | TATA-box | TATA | 1152 | 4 | core promoter element around -30 of transcription start |
| *Solyc10g054070* | TATA-box | TATAAAA | 1178 | 7 | core promoter element around -30 of transcription start |
| *Solyc10g054070* | TATA-box | TATAAA | 1179 | 6 | core promoter element around -30 of transcription start |
| *Solyc10g054070* | TATA-box | TATATAA | 1180 | 7 | core promoter element around -30 of transcription start |
| *Solyc10g054070* | TATA-box | TATATA | 1181 | 6 | core promoter element around -30 of transcription start |
| *Solyc10g054070* | TATA-box | ATATAA | 1182 | 6 | core promoter element around -30 of transcription start |
| *Solyc10g054070* | TATA-box | TATA | 1183 | 4 | core promoter element around -30 of transcription start |
| *Solyc10g054070* | TATA-box | TATACA | 1198 | 6 | core promoter element around -30 of transcription start |
| *Solyc10g054070* | TATA-box | TATATA | 1200 | 6 | core promoter element around -30 of transcription start |
| *Solyc10g054070* | TATA-box | TATA | 1202 | 4 | core promoter element around -30 of transcription start |
| *Solyc10g054070* | TATA-box | ATATAA | 1232 | 6 | core promoter element around -30 of transcription start |
| *Solyc10g054070* | TATA-box | TATA | 1233 | 4 | core promoter element around -30 of transcription start |
| *Solyc10g054070* | TATA-box | ATATAT | 1273 | 6 | core promoter element around -30 of transcription start |
| *Solyc10g054070* | TATA-box | TATATA | 1274 | 6 | core promoter element around -30 of transcription start |
| *Solyc10g054070* | TATA-box | ATATAT | 1275 | 6 | core promoter element around -30 of transcription start |
| *Solyc10g054070* | TATA-box | TATA | 1276 | 4 | core promoter element around -30 of transcription start |
| *Solyc10g054070* | TATA-box | ATTATA | 1279 | 6 | core promoter element around -30 of transcription start |
| *Solyc10g054070* | TATA-box | TATAA | 1280 | 5 | core promoter element around -30 of transcription start |
| *Solyc10g054070* | TATA-box | TATA | 1281 | 4 | core promoter element around -30 of transcription start |
| *Solyc10g054070* | TATA-box | ATATAA | 1297 | 6 | core promoter element around -30 of transcription start |
| *Solyc10g054070* | TATA-box | TATA | 1298 | 4 | core promoter element around -30 of transcription start |
| *Solyc10g054070* | TATA-box | TATA | 1319 | 4 | core promoter element around -30 of transcription start |
| *Solyc10g054070* | TATA-box | TATAAAA | 1368 | 7 | core promoter element around -30 of transcription start |
| *Solyc10g054070* | TATA-box | TATAAA | 1369 | 6 | core promoter element around -30 of transcription start |
| *Solyc10g054070* | TATA-box | TATAA | 1370 | 5 | core promoter element around -30 of transcription start |
| *Solyc10g054070* | TATA-box | TATA | 1371 | 4 | core promoter element around -30 of transcription start |
| *Solyc10g054070* | TATA-box | TATACA | 1465 | 6 | core promoter element around -30 of transcription start |
| *Solyc10g054070* | TATA-box | TATA | 1467 | 4 | core promoter element around -30 of transcription start |
| *Solyc10g054070* | TATA-box | TATAAAA | 1539 | 7 | core promoter element around -30 of transcription start |
| *Solyc10g054070* | TATA-box | TATAAA | 1540 | 6 | core promoter element around -30 of transcription start |
| *Solyc10g054070* | TATA-box | TATAA | 1541 | 5 | core promoter element around -30 of transcription start |
| *Solyc10g054070* | TATA-box | TATA | 1542 | 4 | core promoter element around -30 of transcription start |
| *Solyc10g054070* | TATA-box | TATA | 1659 | 4 | core promoter element around -30 of transcription start |
| *Solyc10g054070* | TATA-box | TATAAAT | 1683 | 7 | core promoter element around -30 of transcription start |
| *Solyc10g054070* | TATA-box | TATAAA | 1684 | 6 | core promoter element around -30 of transcription start |
| *Solyc10g054070* | TATA-box | TATAA | 1685 | 5 | core promoter element around -30 of transcription start |
| *Solyc10g054070* | TATA-box | TATA | 1686 | 4 | core promoter element around -30 of transcription start |
| *Solyc10g054070* | TATA-box | ATTATA | 1716 | 6 | core promoter element around -30 of transcription start |
| *Solyc10g054070* | TATA-box | TATAA | 1717 | 5 | core promoter element around -30 of transcription start |
| *Solyc10g054070* | TATA-box | TATA | 1718 | 4 | core promoter element around -30 of transcription start |
| *Solyc10g054070* | TATA-box | TATA | 1869 | 4 | core promoter element around -30 of transcription start |
| *Solyc10g054070* | TATA-box | TATAA | 1970 | 5 | core promoter element around -30 of transcription start |
| *Solyc10g054070* | TATA-box | TATA | 1971 | 4 | core promoter element around -30 of transcription start |
| *Solyc10g054070* | ARE | AAACCA | 245 | 6 | cis-acting regulatory element essential for the anaerobic induction |
| *Solyc10g054070* | ARE | AAACCA | 411 | 6 | cis-acting regulatory element essential for the anaerobic induction |
| *Solyc10g054070* | ARE | AAACCA | 1433 | 6 | cis-acting regulatory element essential for the anaerobic induction |
| *Solyc10g054070* | Box 4 | ATTAAT | 1305 | 6 | part of a conserved DNA module involved in light responsiveness |
| *Solyc10g054070* | CAAT-box | CAAAT | 232 | 5 | common cis-acting element in promoter and enhancer regions |
| *Solyc10g054070* | CAAT-box | CCAAT | 243 | 5 | common cis-acting element in promoter and enhancer regions |
| *Solyc10g054070* | CAAT-box | CAAAT | 257 | 5 | common cis-acting element in promoter and enhancer regions |
| *Solyc10g054070* | CAAT-box | CAAAT | 307 | 5 | common cis-acting element in promoter and enhancer regions |
| *Solyc10g054070* | CAAT-box | CAAAT | 563 | 5 | common cis-acting element in promoter and enhancer regions |
| *Solyc10g054070* | CAAT-box | CAAAT | 1018 | 5 | common cis-acting element in promoter and enhancer regions |
| *Solyc10g054070* | CAAT-box | CCCAATTT | 1058 | 8 | common cis-acting element in promoter and enhancer regions |
| *Solyc10g054070* | CAAT-box | CCAAT | 1060 | 5 | common cis-acting element in promoter and enhancer regions |
| *Solyc10g054070* | CAAT-box | CAAAT | 1085 | 5 | common cis-acting element in promoter and enhancer regions |
| *Solyc10g054070* | CAAT-box | CAAAT | 1144 | 5 | common cis-acting element in promoter and enhancer regions |
| *Solyc10g054070* | CAAT-box | CAAAT | 1287 | 5 | common cis-acting element in promoter and enhancer regions |
| *Solyc10g054070* | CAAT-box | CAAAT | 1356 | 5 | common cis-acting element in promoter and enhancer regions |
| *Solyc10g054070* | CAAT-box | CCAAT | 1458 | 5 | common cis-acting element in promoter and enhancer regions |
| *Solyc10g054070* | CAAT-box | CCAAT | 1510 | 5 | common cis-acting element in promoter and enhancer regions |
| *Solyc10g054070* | CAAT-box | CCCAATTT | 1806 | 8 | common cis-acting element in promoter and enhancer regions |
| *Solyc10g054070* | CAAT-box | CCAAT | 1808 | 5 | common cis-acting element in promoter and enhancer regions |
| *Solyc10g054070* | CAAT-box | CCAAT | 1943 | 5 | common cis-acting element in promoter and enhancer regions |
| *Solyc10g054070* | GT1-motif | GGTTAAT | 953 | 7 | light responsive element |
| *Solyc10g054070* | GT1-motif | GGTTAA | 954 | 6 | light responsive element |
| *Solyc10g054070* | LAMP-element | CTTTATCA | 1840 | 8 | part of a light responsive element |
| *Solyc10g054070* | TCT-motif | TCTTAC | 1375 | 6 | part of a light responsive element |
| *Solyc10g054070* | TGACG-motif | TGACG | 278 | 5 | cis-acting regulatory element involved in the MeJA-responsiveness |
| *Solyc10g054070* | TGACG-motif | TGACG | 422 | 5 | cis-acting regulatory element involved in the MeJA-responsiveness |
| *Solyc10g054070* | chs-CMA1a | TTACTTAA | 1520 | 8 | part of a light responsive element |
| *Solyc10g054070* | CGTCA-motif | CGTCA | 278 | 5 | cis-acting regulatory element involved in the MeJA-responsiveness |
| *Solyc10g054070* | CGTCA-motif | CGTCA | 422 | 5 | cis-acting regulatory element involved in the MeJA-responsiveness |
| *Solyc10g054070* | TCA-element | TCAGAAGAGG | 725 | 9 | cis-acting element involved in salicylic acid responsiveness |
| *Solyc11g064800* | chs-CMA1a | TTACTTAA | 1099 | 8 | part of a light responsive element |
| *Solyc11g064800* | CGTCA-motif | CGTCA | 407 | 5 | cis-acting regulatory element involved in the MeJA-responsiveness |
| *Solyc11g064800* | ABRE | ACGTG | 409 | 5 | cis-acting element involved in the abscisic acid responsiveness |
| *Solyc11g064800* | ABRE | ACGTG | 909 | 5 | cis-acting element involved in the abscisic acid responsiveness |
| *Solyc11g064800* | TGA-element | AACGAC | 58 | 6 | auxin-responsive element |
| *Solyc11g064800* | TGA-element | AACGAC | 1647 | 6 | auxin-responsive element |
| *Solyc11g064800* | TGACG-motif | TGACG | 407 | 5 | cis-acting regulatory element involved in the MeJA-responsiveness |
| *Solyc11g064800* | TCT-motif | TCTTAC | 293 | 6 | part of a light responsive element |
| *Solyc11g064800* | MBS | CAACTG | 1634 | 6 | MYB binding site involved in drought-inducibility |
| *Solyc11g064800* | TATA-box | TATA | 122 | 4 | core promoter element around -30 of transcription start |
| *Solyc11g064800* | TATA-box | ATTATA | 244 | 6 | core promoter element around -30 of transcription start |
| *Solyc11g064800* | TATA-box | TATAA | 245 | 5 | core promoter element around -30 of transcription start |
| *Solyc11g064800* | TATA-box | TATA | 246 | 4 | core promoter element around -30 of transcription start |
| *Solyc11g064800* | TATA-box | TAAAGATT | 442 | 8 | core promoter element around -30 of transcription start |
| *Solyc11g064800* | TATA-box | ATTATA | 651 | 6 | core promoter element around -30 of transcription start |
| *Solyc11g064800* | TATA-box | TATAA | 652 | 5 | core promoter element around -30 of transcription start |
| *Solyc11g064800* | TATA-box | TATA | 653 | 4 | core promoter element around -30 of transcription start |
| *Solyc11g064800* | TATA-box | TATA | 763 | 4 | core promoter element around -30 of transcription start |
| *Solyc11g064800* | TATA-box | ATATAT | 782 | 6 | core promoter element around -30 of transcription start |
| *Solyc11g064800* | TATA-box | TATA | 783 | 4 | core promoter element around -30 of transcription start |
| *Solyc11g064800* | TATA-box | TATA | 799 | 4 | core promoter element around -30 of transcription start |
| *Solyc11g064800* | TATA-box | TACAAAA | 834 | 7 | core promoter element around -30 of transcription start |
| *Solyc11g064800* | TATA-box | ATTATA | 933 | 6 | core promoter element around -30 of transcription start |
| *Solyc11g064800* | TATA-box | TATAA | 934 | 5 | core promoter element around -30 of transcription start |
| *Solyc11g064800* | TATA-box | TATA | 935 | 4 | core promoter element around -30 of transcription start |
| *Solyc11g064800* | TATA-box | ATATAA | 1093 | 6 | core promoter element around -30 of transcription start |
| *Solyc11g064800* | TATA-box | TATA | 1094 | 4 | core promoter element around -30 of transcription start |
| *Solyc11g064800* | TATA-box | ATATAT | 1109 | 6 | core promoter element around -30 of transcription start |
| *Solyc11g064800* | TATA-box | TATA | 1110 | 4 | core promoter element around -30 of transcription start |
| *Solyc11g064800* | TATA-box | TATAA | 1201 | 5 | core promoter element around -30 of transcription start |
| *Solyc11g064800* | TATA-box | TATA | 1202 | 4 | core promoter element around -30 of transcription start |
| *Solyc11g064800* | TATA-box | ATATAA | 1207 | 6 | core promoter element around -30 of transcription start |
| *Solyc11g064800* | TATA-box | TATA | 1208 | 4 | core promoter element around -30 of transcription start |
| *Solyc11g064800* | TATA-box | TATA | 1252 | 4 | core promoter element around -30 of transcription start |
| *Solyc11g064800* | TATA-box | TACAAAA | 1267 | 7 | core promoter element around -30 of transcription start |
| *Solyc11g064800* | TATA-box | TATATA | 1490 | 6 | core promoter element around -30 of transcription start |
| *Solyc11g064800* | TATA-box | TATA | 1492 | 4 | core promoter element around -30 of transcription start |
| *Solyc11g064800* | TATA-box | TATATA | 1512 | 6 | core promoter element around -30 of transcription start |
| *Solyc11g064800* | TATA-box | ATATAT | 1513 | 6 | core promoter element around -30 of transcription start |
| *Solyc11g064800* | TATA-box | TATATA | 1514 | 6 | core promoter element around -30 of transcription start |
| *Solyc11g064800* | TATA-box | ATATAA | 1515 | 6 | core promoter element around -30 of transcription start |
| *Solyc11g064800* | TATA-box | TATA | 1516 | 4 | core promoter element around -30 of transcription start |
| *Solyc11g064800* | TATA-box | ATTATA | 1588 | 6 | core promoter element around -30 of transcription start |
| *Solyc11g064800* | TATA-box | TATAA | 1589 | 5 | core promoter element around -30 of transcription start |
| *Solyc11g064800* | TATA-box | TATA | 1590 | 4 | core promoter element around -30 of transcription start |
| *Solyc11g064800* | TATA-box | TATA | 1773 | 4 | core promoter element around -30 of transcription start |
| *Solyc11g064800* | TATA-box | TATA | 1881 | 4 | core promoter element around -30 of transcription start |
| *Solyc11g064800* | TATA-box | TATA | 1911 | 4 | core promoter element around -30 of transcription start |
| *Solyc11g064800* | TC-rich repeats | GTTTTCTTAC | 293 | 9 | cis-acting element involved in defense and stress responsiveness |
| *Solyc11g064800* | Box 4 | ATTAAT | 85 | 6 | part of a conserved DNA module involved in light responsiveness |
| *Solyc11g064800* | Box 4 | ATTAAT | 1730 | 6 | part of a conserved DNA module involved in light responsiveness |
| *Solyc11g064800* | G-box | CACGTC | 408 | 6 | cis-acting regulatory element involved in light responsiveness |
| *Solyc11g064800* | G-box | CCACGTAA | 908 | 8 | cis-acting regulatory element involved in light responsiveness |
| *Solyc11g064800* | G-box | TACGTG | 909 | 6 | cis-acting regulatory element involved in light responsiveness |
| *Solyc11g064800* | CAAT-box | CAAAT | 4 | 5 | common cis-acting element in promoter and enhancer regions |
| *Solyc11g064800* | CAAT-box | CAAAT | 141 | 5 | common cis-acting element in promoter and enhancer regions |
| *Solyc11g064800* | CAAT-box | CAAAT | 561 | 5 | common cis-acting element in promoter and enhancer regions |
| *Solyc11g064800* | CAAT-box | CAAAT | 593 | 5 | common cis-acting element in promoter and enhancer regions |
| *Solyc11g064800* | CAAT-box | CAAAT | 728 | 5 | common cis-acting element in promoter and enhancer regions |
| *Solyc11g064800* | CAAT-box | CCAAT | 733 | 5 | common cis-acting element in promoter and enhancer regions |
| *Solyc11g064800* | CAAT-box | CAAAT | 899 | 5 | common cis-acting element in promoter and enhancer regions |
| *Solyc11g064800* | CAAT-box | CCAAT | 1237 | 5 | common cis-acting element in promoter and enhancer regions |
| *Solyc11g064800* | CAAT-box | CAAAT | 1386 | 5 | common cis-acting element in promoter and enhancer regions |
| *Solyc11g064800* | CAAT-box | CAAAT | 1410 | 5 | common cis-acting element in promoter and enhancer regions |
| *Solyc11g064800* | CAAT-box | CCAAT | 1467 | 5 | common cis-acting element in promoter and enhancer regions |
| *Solyc11g064800* | P-box | CCTTTTG | 285 | 7 | gibberellin-responsive element |
| *Solyc11g064800* | P-box | CCTTTTG | 843 | 7 | gibberellin-responsive element |

**Table S2.** Primers used in this study

| **Gene** | **Primer** | **Sequence (5’-3’)** | **Purpose** |
| --- | --- | --- | --- |
| *Solyc01g110140* | qSlSRS1-F | GCAGCTTCAAAATGGGCCAA | qRT-PCR |
|  | qSlSRS1-R | GCTGGAGAACTCACTTCCCC |  |
| *Solyc02g062400* | qSlSRS2-F | AGCAGCAGCCAAGCATTAGT |  |
|  | qSlSRS2-R | TGGTGTTGGTGTTGTGGAGT |  |
| *Solyc02g084680* | qSlSRS3-F | GCAGCAGGAGACCACCAATA |  |
|  | qSlSRS3-R | TCGGCCCGATACCTAATCCA |  |
| *Solyc03g033680* | qSlSRS4-F | GAATGACGTGGCTGTAGGGG |  |
|  | qSlSRS4-R | AAACCTGCCACGAAAGTGTTG |  |
| *Solyc04g080970* | qSlSRS5-F | CAACGAGCACATCCTCCTCA |  |
|  | qSlSRS5-R | CATCAGTACCACCACCGACA |  |
| *Solyc08g077450* | qSlSRS6-F | TCTGGGTCTCCTGTGGTTGA |  |
|  | qSlSRS6-R | ATCCTTTCTGCTCAGTGGCC |  |
| *Solyc10g054070* | qSlSRS7-F | TACCCATCTCTACCAGGCGT |  |
|  | qSlSRS7-R | CCACCTGTTTGTAATAGCGCG |  |
| *Solyc11g064800* | qSlSRS8-F | CTTGGAGGTGGGGGTTCAAT |  |
|  | qSlSRS8-R | TAGTTGCCTCTCACGCCTTC |  |
| *Solyc01g110140* | SlSRS1-pGreen-F | CATTCTACAACTACATCTAGAATGATGAGGAGGGAAGAAGAAGAA | Subcellular localization assay |
|  | SlSRS1-pGreen-R | GACCGGCCGGTGGATCCCGGGAGATGATTTTGGGTATTGAAAAA |  |
| *Solyc02g062400* | SlSRS2-pGreen-F | CATTCTACAACTACATCTAGAATGGCTAATTTCTTTTCATTAGGTG |  |
|  | SlSRS2-pGreen-R | GACCGGCCGGTGGATCCCGGGAGGAGATCTTGAAGGTGGAAAAAA |  |
| *Solyc02g084680* | SlSRS3-pGreen-F | CATTCTACAACTACATCTAGAATGGCTGGCTTCTTTTCACTAGGTG |  |
|  | SlSRS3-pGreen-R | GACCGGCCGGTGGATCCCGGGAGATCTTGGAGGTGGAAAGAATTG |  |
| *Solyc03g033680* | SlSRS4-pGreen-F | CATTCTACAACTACATCTAGAATGGCCGAATTCTTTTCACTAGGA |  |
|  | SlSRS4-pGreen-R | GACCGGCCGGTGGATCCCGGGAGATCTTGAAGGTGGAAAAAATTGA |  |
| *Solyc04g080970* | SlSRS5-pGreen-F | CATTCTACAACTACATCTAGAATGTCTGGATTCTTCACACTAGGA |  |
|  | SlSRS5-pGreen-R | GACCGGCCGGTGGATCCCGGGAGGCCGGGGAGGTGGAAAGAATTGC |  |
| *Solyc08g077450* | SlSRS6-pGreen-F | CATTCTACAACTACATCTAGAATGGCTGCGTCGTCGCCTGCCCTA |  |
|  | SlSRS6-pGreen-R | GACCGGCCGGTGGATCCCGGGCAGTTCTTCTATATCCTCAAGTGA |  |
| *Solyc10g054070* | SlSRS7-pGreen-F | CATTCTACAACTACATCTAGAATGATGATGAGAGAAGATGAAGGA |  |
|  | SlSRS7-pGreen-R | GACCGGCCGGTGGATCCCGGGGGATGATTTTGGGTATTGGAATAA |  |
| *Solyc11g064800* | SlSRS8-pGreen-F | CATTCTACAACTACATCTAGAATGTGGTCTGCATCAGCATCGTCTA |  |
|  | SlSRS8-pGreen-R | GACCGGCCGGTGGATCCCGGGATTTATTTGATTACCATAATTTGAT |  |
| *Solyc01g110140* | SlSRS1-pGBKT7-F | ATGGCCATGGAGGCCGAATTCATGATGAGGAGGGAAGAAGAAGAA | Transcriptional activation activity assay |
|  | SlSRS1-pGBKT7-R | CCGCTGCAGGTCGACGGATCCTCAAGATGATTTTGGGTATTGAAA |  |
| *Solyc02g062400* | SlSRS2-pGBKT7-F | ATGGCCATGGAGGCCGAATTCATGGCTAATTTCTTTTCATTAGGTG |  |
|  | SlSRS2-pGBKT7-R | CCGCTGCAGGTCGACGGATCCTCAAGGAGATCTTGAAGGTGGAAAA |  |
| *Solyc02g084680* | SlSRS3-pGBKT7-F | ATGGCCATGGAGGCCGAATTCATGGCTGGCTTCTTTTCACTAGGTG |  |
|  | SlSRS3-pGBKT7-R | CCGCTGCAGGTCGACGGATCCTCAAGATCTTGGAGGTGGAAAGAAT |  |
| *Solyc03g033680* | SlSRS4-pGBKT7-F | ATGGCCATGGAGGCCGAATTCATGGCCGAATTCTTTTCACTAGGA |  |
|  | SlSRS4-pGBKT7-R | CCGCTGCAGGTCGACGGATCCTCAAGATCTTGAAGGTGGAAAAAAT |  |
| *Solyc04g080970* | SlSRS5-pGBKT7-F | ATGGCCATGGAGGCCGAATTCATGTCTGGATTCTTCACACTAGGA |  |
|  | SlSRS5-pGBKT7-R | CCGCTGCAGGTCGACGGATCCTTAAGGCCGGGGAGGTGGAAAGAAT |  |
| *Solyc08g077450* | SlSRS6-pGBKT7-F | ATGGCCATGGAGGCCGAATTCATGGCTGCGTCGTCGCCTGCCCTA |  |
|  | SlSRS6-pGBKT7-R | CCGCTGCAGGTCGACGGATCCTTACAGTTCTTCTATATCCTCAAG |  |
| *Solyc10g054070* | SlSRS7-pGBKT7-F | ATGGCCATGGAGGCCGAATTCATGATGATGAGAGAAGATGAAGGA |  |
|  | SlSRS7-pGBKT7-R | CCGCTGCAGGTCGACGGATCCTTAGGATGATTTTGGGTATTGGAA |  |
| *Solyc11g064800* | SlSRS8-pGBKT7-F | ATGGCCATGGAGGCCGAATTCATGTGGTCTGCATCAGCATCGTCTA |  |
|  | SlSRS8-pGBKT7-R | CCGCTGCAGGTCGACGGATCCTCAATTTATTTGATTACCATAATTT |  |
| *Solyc01g110140* | SlSRS1-pEAQ-F | AGTTGACTGTATCGCCGACCGGTATGATGAGGAGGGAAGAAGAAGAA | Dual-luciferase assay |
|  | SlSRS1-pEAQ-R | ATGAAACCAGAGTTAAAGGCCTTTATCAAGATGATTTTGGGTATTGAAA |  |
| *Solyc04g080970* | SlSRS5-pEAQ-F | AGTTGACTGTATCGCCGACCGGTATGTCTGGATTCTTCACACTAGGA |  |
|  | SlSRS5-pEAQ-R | ATGAAACCAGAGTTAAAGGCCTTTATTAAGGCCGGGGAGGTGGAAAGAAT |  |
| *Solyc10g054070* | SlSRS7-pEAQ-F | AGTTGACTGTATCGCCGACCGGTATGATGATGAGAGAAGATGAAGGA |  |
|  | SlSRS7-pEAQ-R | ATGAAACCAGAGTTAAAGGCCTTTATTAGGATGATTTTGGGTATTGGAA |  |
| *Solyc11g064800* | SlSRS8-pEAQ-F | AGTTGACTGTATCGCCGACCGGTATGTGGTCTGCATCAGCATCGTCTA |  |
|  | SlSRS8-pEAQ-R | ATGAAACCAGAGTTAAAGGCCTTTATCAATTTATTTGATTACCATAATTT |  |
